# Modified internucleoside linkages for nuclease-resistant oligonucleotides

**DOI:** 10.1039/d0cb00136h

**Published:** 2020-12-08

**Authors:** Guillaume Clavé, Maeva Reverte, Jean-Jacques Vasseur, Michael Smietana

**Affiliations:** IBMM, Univ. Montpellier, CNRS, ENSCM Montpellier France jean-jacques.vasseur@umontpellier.fr michael.smietana@umontpellier.fr

## Abstract

In the past few years, several drugs derived from nucleic acids have been approved for commercialization and many more are in clinical trials. The sensitivity of these molecules to nuclease digestion *in vivo* implies the need to exploit resistant non-natural nucleotides. Among all the possible modifications, the one concerning the internucleoside linkage is of particular interest. Indeed minor changes to the natural phosphodiester may result in major modifications of the physico-chemical properties of nucleic acids. As this linkage is a key element of nucleic acids’ chemical structures, its alteration can strongly modulate the plasma stability, binding properties, solubility, cell penetration and ultimately biological activity of nucleic acids. Over the past few decades, many research groups have provided knowledge about non-natural internucleoside linkage properties and participated in building biologically active nucleic acid derivatives. The recent renewing interest in nucleic acids as drugs, demonstrated by the emergence of new antisense, siRNA, aptamer and cyclic dinucleotide molecules, justifies the review of all these studies in order to provide new perspectives in this field. Thus, in this review we aim at providing the reader insights into modified internucleoside linkages that have been described over the years whose impact on annealing properties and resistance to nucleases have been evaluated in order to assess their potential for biological applications. The syntheses of modified nucleotides as well as the protocols developed for their incorporation within oligonucleotides are described. Given the intended biological applications, the modifications described in the literature that have not been tested for their resistance to nucleases are not reported.

## Abstract 2

This review covers comprehensively the chemical synthesis and hybridization properties of modified internucleoside linkages with reported nuclease resistances and discusses their assets for potential biological applications.

## Graphical abstract

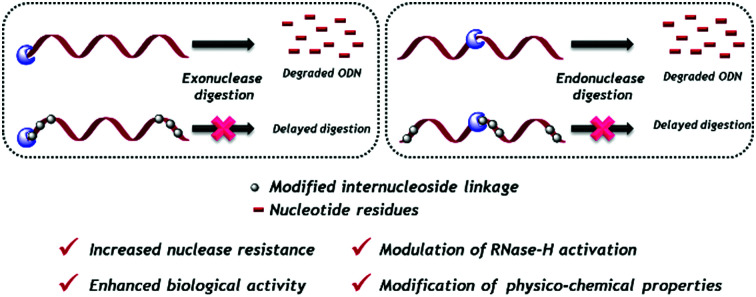

## 1. Introduction

Since the discovery of their structures and their roles as carriers of genetic information, the biological understanding of deoxyribonucleic acid (DNA) and ribonucleic acid (RNA) has evolved to versatile bioscaffolds with applications in many areas related to biology. Important discoveries include their therapeutic use as antisense (AS) agents,^1,2^ small interfering RNAs (siRNAs),^3,4^ CRISPR (Clustered Regularly Interspaced Short Palindromic Repeats) associated protein 9 (CRISPR-Cas9),^5,6^ antigen (triplex-forming oligonucleotide)^7,8^ molecules, aptamers,^9–11^ and primers for gene amplification through polymerase chain reaction (PCR)^12,13^ or gene sequencing,^14–17^ and many other applications related to biotechnology such as the elaboration of DNA microarrays,^18,19^ site-specific mutagenesis,^20^ Southern blotting and Northern blotting.^21^

In the specific field of DNA-based *in vivo* gene regulation therapies, nuclease resistance is a prerequisite for oligodeoxynucleotides (ODN) to allow them to reach their target and have observable therapeutic effects in the presence of a plethora of nucleases in serum and cells.^22–25^ In order to improve their resistance to nuclease digestion, numerous chemical modifications have been developed over the years.^26–28^ Each component of the DNA structure has been envisioned to be modified and can be categorized by modification of (1) the internucleoside linkage, (2) the deoxyribose/ribose, (3) the nucleobase, and (4) the derivatization or bioconjugation of the ODN.^29–36^ However, it is essential that the nuclease resistance of any newly synthesised backbone-, ribose-, or base-modification has to be evaluated before considering therapeutic or biotechnological applications.^37–40^

The discovery that an ODN is able to inhibit *in cellulo* viral replication dates back to 1978.^41^ After 20 years of research the first antisense oligonucleotide (ODN-AS) was commercialized in 1998 against cytomegalovirus retinitis (Fomivirsen, commercialized as Vitravene®).^42^ Since then several ODN based drugs carrying different modifications at the internucleoside linkage as will be illustrated herein have been approved by the Food and Drug Administration (FDA). In 2019 Waylivra® was the eighth antisense drug to gain approval for commercialization^43^ and dozens are currently in clinical trials. This demonstrates that after several decades of efforts the pharmaceutical industry has managed to exploit the exceptional therapeutic properties of modified ODN, allowing considering their applications in numerous pathologies in the future. Besides, in 2019 the first patient-customized ODN-AS therapy was reported.^44^ Indeed fourteen months after the diagnosis of Batten disease in a 6-year-old child, the patient was treated with a custom-designed ODN-AS (named Milasen after the patient, Mila Makovec) after identifying the genetic mutation responsible for her pathology. It should be further mentioned that in 2018 the first siRNA was approved by the FDA: Patisiran (Onpattro®).^45^ The double stranded ORN possesses a natural phosphodiester (PO) backbone and a few 2′-OMe modified ribose units but it is formulated and protected from digestion by nucleases in the form of lipid nanoparticles, which enables it to reach its biological target. Moreover, in 2019 the second siRNA was approved by the FDA: Givosiran (Givlaari®).^46^ This siRNA is administered for adults with acute hepatic porphyria. The double stranded siRNA is covalently linked to a ligand containing three *N*-acetylgalactosamine residues to enable delivery of the siRNA to the targeted hepatocytes.

Aptamers are nucleic acid molecules that can be compared to antibodies. Indeed they are able to fold into complex 3D structures that bind to specific targets. Although a few aptamers exist naturally as the ligand-binding elements of riboswitches,^47^ aptamers are generally obtained by *in vitro* selection for a specific target (systematic evolution of ligands by exponential enrichment, SELEX).^48^ More recently, SELEX technology was developed *in cellulo*.^49^ Aptamers can be used for therapeutics, sensing, environmental screening, drug delivery, allosteric modulation and natural product synthesis applications.^10^ Pegaptanib sodium (Macugen®), a 28-mer RNA covalently linked to two branched 20 kDa polyethylene glycol (PEG) chains, was the first aptamer drug approved for the treatment of wet AMD (age-related macular degeneration) but numerous other aptamers are currently in clinical trials.^50^

In addition to these sequences of nucleic acid derivatives, cyclic dinucleotides (CDN) are also emerging through the targeting of STING (stimulator of interferon genes) as new nucleic acid based therapeutics. STING is a key element in the functioning of the innate immune response by stimulating the production of type I interferons that limit the infection of neighboring cells. Several recent studies have recently pointed out the interest of STING stimulation by synthetic CDN for the treatment of autoinflammatory disease and cancer.^51–55^ However, CDN carrying natural PO linkages suffer from the same drawback as ODN-AS concerning their degradation by nucleases. The synthesis of CDN modified with non-natural nuclease resistant internucleoside linkages could expand their use as therapeutic agents.

At this point it is important to note that the backbone modification of therapeutic oligonucleotides is absolutely essential and more important than ribose and nucleobase modifications. Indeed although the latter are also of great importance for many physico-chemical parameters, the internucleoside linkage is the recognition site for nucleases. Consequentially, the choice of the backbone used is of prime importance. Moreover, the negative charges carried by the natural linkage limit the cellular penetration of ODN. Consequentially, the site-specific replacements of natural PO with alternative structural motifs can enhance the ODN cellular penetration. For instance, neutral or even positively charged alternative linkages have been envisaged. At physiological pH, chimeric PO/positively charged moiety-ODN may result in zwitterionic or cationic backbone structures.^56–59^ Different research groups have developed isoelectronic structures to replace the PO linkage assuming that the annealing properties would be conserved or even enhanced while achieving significant resistance to nucleases. Many publications concern the total replacement of the PO linkage in order to introduce a non-phosphorus derived internucleoside linkage. As will be illustrated, it generally achieves a high or total resistance to nuclease digestion.

In this review, we aim to focus on non-natural internucleoside linkages whose nuclease resistance has been evaluated. Our efforts aim to provide to the community of scientists working on the biological applications of nucleic acids a powerful toolbox allowing them to either quickly compare their work to the literature or choose wisely a modified backbone for specific uses. Thus, we have exhaustively identified the modified internucleoside linkages whose resistances to nucleases have been evaluated with at least one commercially available nuclease or serum containing nucleases. We have largely focused on the aspects concerning the chemical synthesis of the modified linkages. Thus, the first synthesis of dimers has been described, as well as the progress made thereafter to incorporate these modifications within ODN by supported synthesis for biological studies. Although this review does not discuss ribose alteration, a few examples of the double modification of both the internucleoside linkage and the ribose moiety are reported. It is important to note that publications describing modifications of the internucleoside linkage without any nuclease resistance evaluation are not reviewed. Moreover, peptide nucleic acids (PNA), which were first described by Nielsen *et al.* in 1991^60^ and are an important class of nucleic acid analogues, are outside the scope of this review. Indeed, the entire backbone has been replaced with neutral *N*-(2-aminoethyl)glycine units. Consequentially, PNA are chemically stable and totally resistant to the hydrolytic activity of nucleases. PNA are able to recognise specific sequences of DNA and RNA and the resulting duplexes exhibit high thermal stability. Therefore, PNA found major applications in the diagnostic and therapeutic fields which have been previously reviewed.^61–66^

## 2. Nucleases

**Scheme 1:**
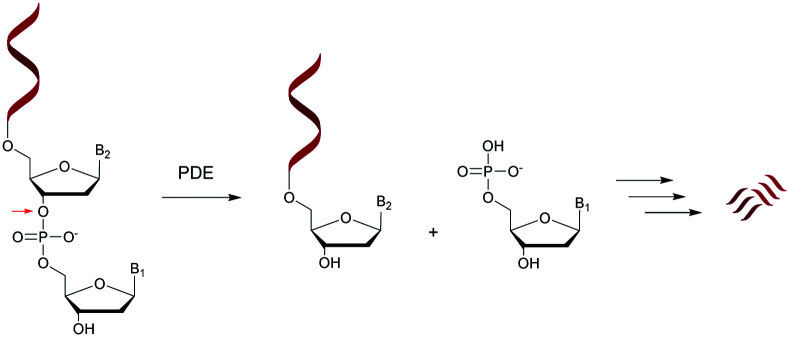
General representation of the hydrolysis of an ODN by a 5′-PDE.

Nucleases are part of the hydrolase family that act on nucleic acids (DNA and RNA) and their derivatives.^67,68^ Specifically, they are phosphodiesterases (usually referred to as cyclic nucleotide phosphodiesterases (PDE)) that hydrolyse one of the two bridging P–O bonds, 3′ or 5′ in a nucleic acid derivative.^69^ Their mechanism of action involves the 3′-phosphate hydrolysis of an intracellular messenger from an active (cyclic AMP or cyclic GMP) to an inactive form. Endonucleases are composed of DNases and RNases whose substrates are deoxyribonucleic and ribonucleic acids respectively. Endonucleases can be nonspecific and are able to hydrolyse all nucleic acid sequences, or can be very specific and are only capable of hydrolysing precise internucleoside linkages from a specific recognition sequence (restriction enzymes). Exonucleases are capable of hydrolysing a nucleotide from the 3′ or 5′ ends of a nucleic acid. While intracellular PDE are involved in a broad range of important cellular functions by regulating the concentrations of cyclic nucleotides,^70^ extracellular PDE exist in snake venoms^67,71^ and act as exonucleases by removing mononucleotide monophosphate units from polynucleotide chains in a stepwise fashion (Scheme 1).

PDE are classified into 11 families (PDE 1 to PDE 11) according to their affinities for AMPc or GMPc, their localizations and their biological functions.^70^ It should be noted that all natural ODN are systematically degraded *in vivo* by PDE within minutes and that DNA can have a half-life of up to several hours for 1–2 kbp. As mentioned above, PDE that hydrolyse phosphodiester bonds of polynucleotide chains are categorized depending on their abilities to cleave phosphodiester bonds at either the 3′ or the 5′ end (exonucleases) or at the center (endonucleases) of DNA or RNA sequences. Finally, PDE have different substrate specificities: DNA and/or RNA; 3′ to 5′ or 5′ to 3′ exonuclease activity; single strand (ss) and/or double strand (ds) and terminal OH or terminal phosphate processing (Table 1). Among all these nucleases, RNase-H is of particular importance for the antisense strategy in order to silence a specific gene *via* the catalytic destruction of its mRNA through the formation of an ODN/mRNA duplex.^72^ Accordingly, the targeted protein biosynthesis will be limited or even extinguished. Unfortunately, only a few modifications induce RNase-H activity. When designing an ODN-AS, it is essential to choose a structure that allows the induction of RNase-H to degrade its complementary RNA target, while providing for itself high resistance to other nucleases.

**Table 1:** Names and activities of the main known phosphodiesterases

Phosphodiesterase	Hydrolytic activities
	Exonuclease activity
Phosphodiesterase I from snake venom phosphodiesterase (SVPDE)	5′ exonuclease targeting ss or ds DNA or RNA. 3′ → 5′ activity
Calf spleen phosphodiesterase (CSPDE)	3′ exonuclease targeting 5′-OH ss or ds DNA or RNA. 3′ → 5′ activity
Phosphodiesterase II from bovine spleen (spleen phosphodiesterase)	3′ exonuclease targeting 5′-OH ss or ds DNA or RNA. 5′ → 3′ activity
Exonuclease III	5′ exonuclease targeting ds DNA/DNA or DNA/RNA. 3′ → 5′ activity
Calf intestinal alkaline phosphatase (CIAP)	Catalyses non-specific dephosphorylation at the 3′ and 5′ ends of a DNA/RNA strand
T4 polymerase digestion	5′ exonuclease targeting ds DNA. 3′ → 5′ activity
	Endonuclease activity
DNase I	Hydrolyses ss or ds DNA producing 3′-OH and 5′-P (preferably takes place at a position adjacent to a pyrimidine)
Nuclease P1	3′ → 5′ activity targeting ss DNA or RNA
Nuclease S1	Hydrolyses ss or ds DNA or RNA
RNase-H	Hydrolyses the RNA strand of a hybrid DNA/RNA duplex
RNase-A	Hydrolyses ss RNA
	Digestion activity
EcoRi 1	Specifically recognizes the palindromic G/AATTC sequence of a DNA duplex
Endonuclease Nsi1	Specifically recognizes the palindromic ATGCA/T sequence of a DNA duplex

## 3. Modified internucleoside linkages

### 3.1 Phosphorus derived internucleoside linkages

**Fig. 1:**
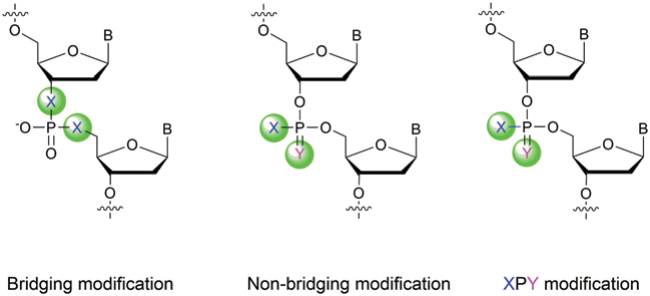
Bridging and non-bridging modifications of the internucleoside phosphodiester linkage. Nomenclature used for the replacements of non-bridging oxygen atoms in modified internucleoside linkages.

Many modifications of the internucleoside linkage have involved the substitution of one or two oxygen atoms of the phosphodiester moiety. The objective is to improve the properties of the resulting ODN strand (*i.e.* annealing properties, nuclease resistance, chemical stability…). Thus, all the modifications that will be described in this section imply the replacement of at least one of the oxygen atoms of the phosphodiester linkage with another atom. This substitution can be carried out not only at the bridging oxygen atoms (3′ and 5′), but also on one or two of the non-bridging oxygen atoms (Fig. 1). In the interest of not overloading the structure of the section, the separation of the modified ODN with bridging or non-bridging modifications will not be made.

When available or introduced by the authors, we tried to use original abbreviations. Otherwise, we choose to designate the *σ* bonded modification (X) before the phosphorous atom and the Π bonded modification (Y) after such as XPY (Fig. 1).

It is important to mention that upon replacement of a single non-bridging oxygen atom the dinucleotide analogues described in this section are synthetized as a mixture of two diastereoisomers.

#### 3.1.1 Phosphorothioate (PS) and thiophosphate (SP) linkages

Phosphorothioate ODN (PS-ODN) belong to the first generation of antisense agents in which one of the non-bridging phosphate oxygen atoms is replaced with a sulphur atom.

**Scheme 2:**
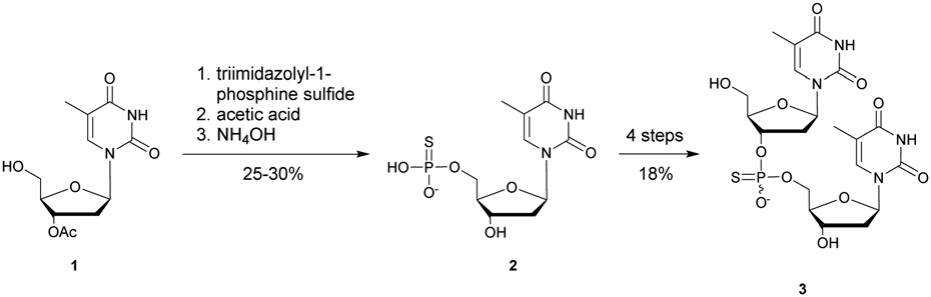
Eckstein synthesis of 5′-phosphorothioate thymidine (**2**)^73^ and dithymidine phosphorothioate (**3**).^74^

In 1966, Eckstein developed the first synthesis of thymidine 5′-phosphorothioate, **2** (Scheme 2).^73^ The synthesis begins with 3′-*O*-acetyl-thymidine, **1**, which reacts with an excess of triimidazolyl-1-phosphine sulfide to phosphorylate the 5′-hydroxyl group. The resulting product is then treated with hot acetic acid and aqueous ammonia to lead to the desired compound **2**.

Eckstein then progressed to the synthesis of a dinucleotide phosphorothioate (Scheme 2).^74^ This compound was obtained in 18% overall yield in 4 steps. Compared to the natural dinucleotide, the phosphorothioate **3** was found to be totally resistant to SVPDE and spleen phosphodiesterase.

After this work, several syntheses of PS-dinucleotides were performed.^75,76^ However, it was only in 1984 that the group of Stec published the first automated synthesis of PS-ODN using elemental sulphur (S_8_) in the P(iii) oxidation step of classical phosphoramidite chemistry.^77^ Numerous research groups have exploited this procedure for years but an interesting alternative to the sulfurizing agent S_8_ was published in 1989 by the group of Beaucage – 3*H*-1,2-benzodithiole-3-one 1,1-dioxide (also known as Beaucage reagent).^78^ The authors demonstrated the superior efficiency of the Beaucage reagent as a sulfurization agent (30 s *versus* 7.5 min for elemental sulphur) thanks to its good solubility in common organic solvents.

In 1983 Eckstein published an important review concerning the PS analogues of nucleotides as tools for the study of biochemical processes. He notably referenced all the enzymes tested at the time on PS analogues (∼40) and pointed out their eventual *P* stereoselectivity. All these results prompted many research groups to synthesize and exploit the phosphorothioate modification for biomedical applications. Given the plethoric number of publications demonstrating the therapeutic potential of PS-ODN in numerous applications,^40^ only a few representative examples will be reviewed herein with a focus on nuclease resistance.

The group of Agarwal published in 1993 an article concerning the exploitation of “self-stabilized” ODN having a hairpin loop structure at their 3′-end to increase their 3′-exonuclease resistance.^79^ They studied both PO and PS versions of the different ODN. The aim of this work was to improve the RNase cleavage of the gag sequence of HIV-1 to inhibit its replication *via* an antisense strategy. Numerous ODN have been studied but only representative examples are detailed (Table 2). Thermal denaturation studies were performed with the 39-mer gag RNA sequence of HIV-1. Results indicate that the presence of the hairpin induces only a slight destabilization of the duplexes in both the PO and PS series. The PS-ODN duplexes are significantly less stable than their PO counterparts, although this does not interfere with their ability to activate RNase-H. Nuclease digestion experiments against SVPDE clearly show the potential of the hairpin structure at the 3′-end of the ODN in preventing 3′-exonuclease activity (*t*_1/2_ > 1000 s for hairpin-ODN compared to 88 s). The effect of the PS linkage was evaluated against the Pol I enzyme (polymerase having exonuclease activity). Results indicate that PS-ODN are more resistant than their PO counterparts and that the hairpin also contributes to the slowdown of the Pol I activity. Similar results were obtained when the different ODN were incubated in FCS. Finally, RNase-H cleavage experiments were performed *in vitro* along with *in cellulo* and *in vivo* studies. The authors were able to demonstrate that the hairpin loop structure does not interfere with RNase-H activity while achieving a significant increase in activity thanks to their better stability *in cellulo* and *in vivo*. Indeed, they observed that 80% of the linear all-PO-ODN was degraded in the liver after 24 h of *in vivo* experiments, but less than 20% of the all-PS hairpin loop structure was degraded. The comparative studies reported in this publication demonstrate the increase in resistance induced by the hairpin loop structure and the PS linkage, opening the way to an improvement of their potential as pharmaceutical agents.

**Table 2:** Thermal denaturation studies (*T*_m_ values) of PS-ODN with complementary RNA and their half-life evaluations against SVPDE, Pol I and fetal calf serum (FCS)^79^

ODN (5′ → 3′)^a^	*T*_m_ with RNA (°C)	*t*_1/2_ b			RNase-H activation
		SVPDE (s)	Pol I (min)	FCS (h)	
[d(CTCTCGCACCCATCTCTCTCCTTCT)]-all-PO	73.1	88	30	—	✓
Hairpin-all-PO^c^	72	>1000	>120	—	✓
[d(CTCTCGCACCCATCTCTCTCCTTCT)]-all-PS	65	—	120	≫16	✓
Hairpin-all-PS^c^	63	—	>240	∼4	✓

a PS and PO refer to the phosphorothioate and phosphodiester internucleoside linkages respectively.

b ODN not tested.

c Hairpin loop structure of the ODN studied.

In 1996 Monia *et al.* published an important study concerning a 17-mer ODN sequence targeting the human Ha-*ras*.^80^ This gene is involved in regulating cell division in response to growth factor stimulation. Its deregulation is involved in many types of cancer growth. First, they studied the effect of replacing an increasing number of PO linkages with PS linkages, from the points of view of both nuclease resistance and antisense activity *in cellulo*. A few representative examples of the ODN studied are listed in Table 3.

**Table 3:** Half-life evaluations of PS-ODN against *Bal*31 endonuclease and antisense activity evaluation in cultured T24 cells^80^

ODN (5′ → 3′)^a^	*t*_1/2_ (min)	AS activity^b^ (%)
d(CCACACCGACGGCGCCC)	5	0
d(C_PS_C_PS_A_PS_C_PS_A_PS_C_PS_C-GACG_PS_G_PS_C_PS_G_PS_C_PS_C_PS_C)	8	35
d(C_PS_C_PS_A_PS_C_PS_A_PS_C_PS_C_PS_-GAC_PS_G_PS_G_PS_C_PS_G_PS_C_PS_C_PS_C)	50	78
[d(CCACACC-GACGGCGCCC)]-all-PS	>50	82

a PS refers to the phosphorothioate internucleoside linkage.

b Percentage of inhibition of Ha-*ras* mRNA expression by activation of RNase-H within the cells by the ODN tested at 0.1 μM.

The sensitivity of the ODN to the increased presence of PO-linkages is clearly demonstrated against *Bal*31 endonuclease. While the all-PS-ODN is totally stable during the course of the experiment, the higher the number of PO linkages, the lower the half-life of the ODN. The consequences of this nuclease sensitivity are observed during the *in cellulo* tests to inhibit Ha-*ras* mRNA expression. The loss in activity is directly correlated with the AS-ODN degradation.

Thereafter, the authors studied the influence of 2′-alkoxy and 2′-fluoro ribose modifications on ODN sequences. These modifications were analyzed for both resistance to nuclease digestion (SVPDE) and AS activity against Ha-*ras* in intact cells. These modifications were reported to be unable to activate RNase-H *in vitro* although this limitation was overcome through the use of chimeric ODN bearing the modified nucleotides only at the extremities of the strands.^81^ Consequentially, the authors synthetized chimeric ODN gapmers flanked with 2′ modified riboses containing sufficient unmodified nucleotides at the center of the strands to ensure the activation of RNase-H. This modification does not directly concern the topic of this review; thus the results will not be detailed. However, such gapmers have then been studied by many research groups and pharmaceutical companies, leading years after to approved drugs (Table 4). These modifications achieved increased resistance to SVPDE (2′-pentoxy > propoxy > methoxy > fluoro = deoxy) and consequentially afforded very good results as antisense molecules *in cellulo*. These results among others have paved the way for the use of chimeric ODN with different types of modifications to increase their resistance to nucleases and hence their effectiveness in therapeutic applications depending on their specific target. Since then, many firms or research groups have developed therapeutic ODN.

**Table 4:** Examples of PS or chimeric antisense oligonucleotides approved or in clinical trials

Compound	Chemical structure^a^	Disease	Status (clinical phase)	Company
Fomivirsen (Vitravene®, ISIS-2922)^82^	PS	CMV retinitis	Approved	Ionis Pharmaceuticals
Mipomersen, (Kynamro®, ISIS-301012)^99^	2′-OMoE chimera	Homozygous familial hypercholesterolemia (HoFH)	Approved	Ionis Pharmaceuticals
Nusinersen (Spinraza®)^100^	2′-OMoE chimera	Spinal muscular atrophy (SMA)	Approved	Biogen/Ionis Pharmaceuticals
Inotersen (Tegsedi®)^101^	2′-OMoE chimera	Hereditary transthyretin amyloidosis (hATTR)	Approved	Akcea Therapeutics/Ionis Pharmaceuticals
Milasen	PS	Batten disease	Approved	Boston Hospital (crowdfunding)
Volanesorsen (Waylivra®)^102^	2′-OMoE chimera	Hypertriglycidemia, familial chylomicronemia syndrome and familial partial lipodystrophy	Approved	Ionis Pharmaceuticals
Oblimersen (Genasense, Augmerosen, G-3139)^103^	PS	Chronic lymphocytic leukemia, malignant melanoma, multiple myeloma, non-small cell lung cancer, acute myeloid leukemia	III	Genta Inc. & Aventis Pharma
Trabedersen (AP-12009)^104^	PS	Oncology-glioblastoma	III	Antisense Pharma
Aganirsen (GS-101)^105^	PS	Corneal neovascularization	III	Gene Signal
Affinitak (ISIS-3521, LY-900003, aprinocarsen)^106^	PS	Non-small cell lung cancer	III	Ionis Pharmaceuticals & Eli Lilly
Custirsen (OGX-011, ISIS-112989, TV-1011)^59^	2′-OMoE chimera	Non-small cell lung cancer, prostate and breast cancer	III	OncoGeneX 42
Drisapersen (PRO-051, GSK-2402968)	2′-OMoE chimera	Duchenne muscular dystrophy	III	Prosensa Therapeutics & GlaxoSmithKline
ProMune46 (CPG-7909, PF-3512676)^107^	PS	Non-small cell lung cancer	III	Pfizer
1018-ISS^108^	PS	Ragweed allergy, hepatitis B, non-Hodgkin's lymphoma and colorectal neoplasms	III	Dynavax Technologies

a PS and OMoE refer to the phosphorothioate internucleoside linkage and 2′-*O*-(2-methoxyethyl) modification of the deoxyribose respectively.

**Scheme 3:**
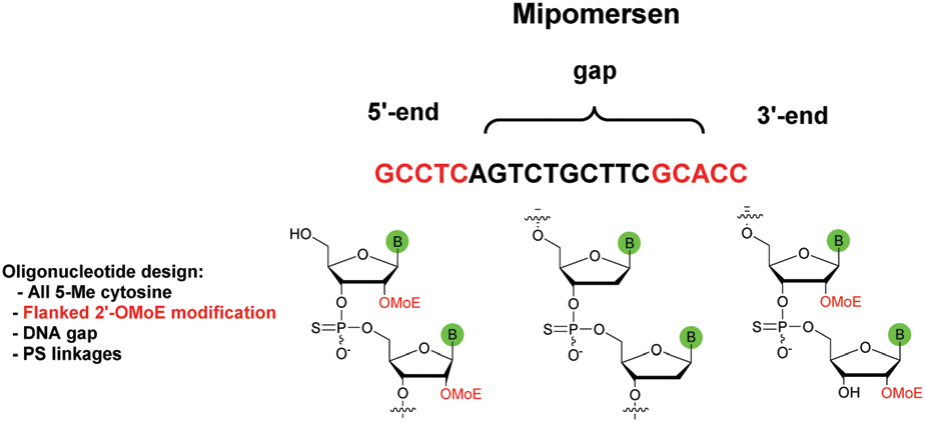
Sequence and chemical structure of Mipomersen.

The first PS-ODN to be placed on the market was Fomivirsen (Vitravene®) marketed by the company Ionis Pharmaceuticals in 1998.^82^ This 21-mer PS-ODN was used in the treatment of cytomegalovirus (CMV) retinitis in immunocompromised patients, especially those with acquired immunodeficiency syndrome (AIDS). Another PS-ODN was approved by the FDA in 2013: Mipomersen (Kynamro®, Scheme 3) developed by Ionis Pharmaceuticals and Genzyme.^83^ This gapmer ODN can inhibit the translation of the messenger coding for apolipoprotein B and consequently decrease the quantity of LDL-cholesterol in patients with homozygous familial hypercholesterolemia. As Monia *et al.* described previously,^80^ it is a chimeric 2′-*O*-(2-methoxyethyl) and 2′-deoxyribonucleotide with phosphorothioate linkages (2′-OMoE-PS-ODN) composed of all-5-Me cytosine residues (Scheme 3).

This modification is generally used as it was demonstrated that 5-Me cytosine enhances the thermal stability of duplexes by ∼+0.5 °C per modification.^36^ Many PS-ODN (or chimeric gapmers) are undergoing clinical trials. This topic has already been extensively reviewed.^1,84,85^ Thus we have reported in Table 4 only a few significant examples of PS or chimeric ODN which are approved or advanced in clinical trials.

**Scheme 4:**
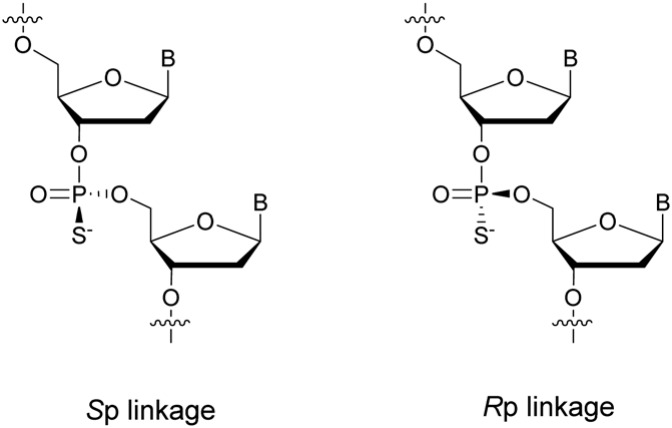
Chemical structures of *S*_p_ and *R*_p_ phosphorothioate chiral linkages.

PS-ODN have *P* chiral centers (*R*_p_/*S*_p_, Scheme 4), and despite considerable research efforts, conventional solid-phase synthesis of PS oligonucleotides produces a mixture of diastereoisomers.

Several studies performed by the group of Stec were devoted to the effect of the *P*-chirality of PS-ODN on their resistance to nucleases compared to natural ODN.^86–88^ This has been possible thanks to the use of diastereomerically pure 5′-*O*-DMTr-3′-*O*-(2-thio-1,3,2-oxathiaphospholane)-nucleosides.^89^ Since then, many methods for the stereocontrolled synthesis of PS-ODN have been developed.^90–98^

In 1995 the group of Stec described the difference in activity of RNase-H during the hydrolysis of a hybridized 15-mer oligoribonucleotide (ORN) to its complementary PO, mix-PS, all-*R*_p_-PS or all-*S*_p_-PS-ODN.^86^ The experiments were conducted with either 1 or 3 equivalents of the ODN compared to the ORN at 28 or 37 °C for 45 min before analysis (Table 5). The results showed that the enzyme is more efficient in degrading the ORN involved in a heteroduplex with the all-*R*_p_-PS-ODN than with the all-*S*_p_-PS-ODN. Logically, the diastereoisomeric mixture is hydrolysed in an intermediate period of time. Interestingly, the introduction of a large excess of ODN relative to the ORN (1 : 3 ratio) limits the stereodependence of the efficiency of RNase-H. The stereodependence is recovered by working at a lower temperature. Years later, the same group published results concerning the resistance of their diastereoisomeric pure PS-ODN against 3′-exonucleases present in human plasma.^88^ The half-lives of the different PS-ODN studied were determined during an experiment consisting of incubating them for 8 h at 37 °C in a 50% human plasma solution. The results showed (in comparable sequence) that the all-*R*_p_-PS-ODN had an increased resistance to 3′-exonucleases.

**Table 5:** Percentages of ORN degradation catalyzed by RNase-H^86^

ORN component (ORN : ODN molar ratio)^a^	Incubation temperature (°C)	ODN component^b^			
		All-PO	Mix-PS	All-*R*_p_-PS	All-*S*_p_-PS
ORN-all-PO (1 : 1)	37	87	53	89	52
ORN-all-PO (1 : 3)	37	96	83	96	75
ORN-all-PO (1 : 3)	28	80	65	86	35

a PS and PO refer to the phosphorothioate and phosphodiester internucleoside linkages respectively.

b ODN sequence d(AGATGTTTGAGCTCT).

At the same time, the all-*S*_p_-PS-ODN analogues were perfectly stable during the course of the experiment. This demonstrates that the 3′-exonucleases are only able to recognize *R*_p_ configuration linkages while being less efficient due to the substitution of the oxygen atom with a sulfur atom. In addition, working with a diastereoisomeric mixture of PS-ODN appears to slow down the overall enzymatic activity of the 3′-exonucleases. Finally, the authors also demonstrated that total resistance to 3′-exonucleases could be obtained thanks to the presence of a single internucleoside linkage of the *S*_p_ configuration at the 3′ end. Noteworthily, the most resistant isomer to exonucleases is the least able to allow activation of RNase-H and *vice versa*.

**Scheme 5:**
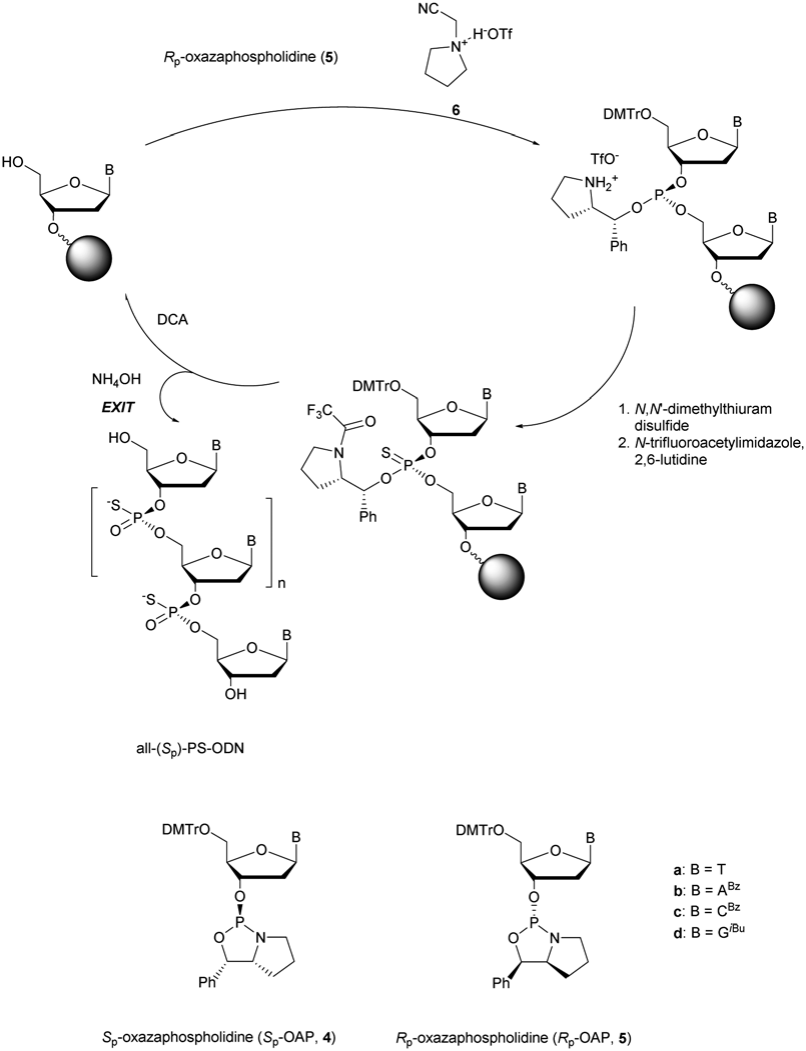
Automated synthesis cycle for stereoregular PS-ODN. Chemical structures of *S*_p_ (**4a–d**) and *R*_p_ (**5a–d**) OAP monomers.

More recently Wan *et al.*^96^ developed original bicyclic oxazaphospholidine (OAP) monomers **4a–d** and **5a–d** (Scheme 5) in order to prepare a series of AS-ODN gapmers modified with chiral phosphorothioate linkages. The objective was to study how the *P*-chirality influences the biophysical and biological properties of these PS-ODN (*T*_m_, enzymatic resistance, *in vitro* and *in vivo* activities, RNase-H activation…). Their results demonstrated unambiguously how the *P*-chirality modulates the therapeutic properties of the isomers, their role in terms of interaction with the target, their activity and their metabolization. The results confirm those obtained by Stec's group concerning the resistance of all-*S*_p_-PS-ODN compared to all-*R*_p_-PS-ODN but the reverse capacity of these stereoisomers PS-ODN to activate RNase-H, leading to catalytic RNA hydrolysis, was reversed. As a conclusion, the best *in vivo* result was obtained with a PS-ODN comprising a mixture of *R*_p_ and *S*_p_ in order to achieve the best compromise between activity and nuclease resistance. The work of Wada's group has recently been exploited to synthesize PS-ODN gapmers of controlled chirality.^109^ The objective was to determine the effect of controlling the PS chirality in the gap region in order to enhance the potency and therapeutic profile of the ODN. The authors determined that the sequences and the chemical structures are the main factors that determine the pharmacological and toxicological properties of PS-ODN gapmers. The conclusion of this study was that stereorandom PS internucleoside linkages offer the best compromise between activity and stability. However, this result did not prevent the scientific community from continuing to be interested in the stereospecific synthesis of PS-ODN.

In 2015 Hall's group published very interesting results concerning the use of 5-benzylthio-1-*H*-tetrazole as an activator instead of classic 1*H*-tetrazole.^110^ Specific interactions during the coupling step allowed the enhancement of the *R*_p_ configuration after sulfurization.

Wada's group also largely contributed to the development of stereocontrolled PS-ODN and PS-ORN synthesis. They used the bicyclic OAP developed by Wan^96^ along with [*N*-(cyanomethyl)pyrrolidinium triflate (**6**) (CMPT) as an acidic activator for the solid phase synthesis of PS-ODN (Scheme 5).^93^

The method is efficient with excellent yields and diastereoselectivities (96–99% yields, d.r. ≥ 99 : 1).

At the same time they developed stereodefined PS-ORN based on the same strategy using 2′-*O*-TBDMS protected nucleosides A^Ac^, T, C^Ac^, G^CE,PAC^ and U.^94^ The ORN synthetized were subjected to thermal denaturation experiments. It was observed that all-*S*_p_-PS-ORN (as well as stereorandom PS) induced a destabilizing effect on a PS-ORN/ORN duplex, whereas a backbone consisting of all-*R*_p_-PS-ORN slightly stabilized the duplex. The solid phase synthesis protocol was improved a few years later by the use of 2′-*O*-2-cyanoethoxymethyl protective groups.^95^

As we have seen in this section, the stereochemistry of phosphorus is of great importance from the point of view of the biological properties of PS-ODN, in particular because of the variable sensitivity to enzymatic digestion by nucleases. Thus, the future of PS-ODN will likely pass through the easy to implement synthesis of stereocontrolled PS-ODN at every phosphorus atom. This would allow chemists to modulate the physico-chemical properties of the ODN according to the intended application. This need is real as shown by the work published on this topic over the past few years.^97,98,111^

**Scheme 6:**
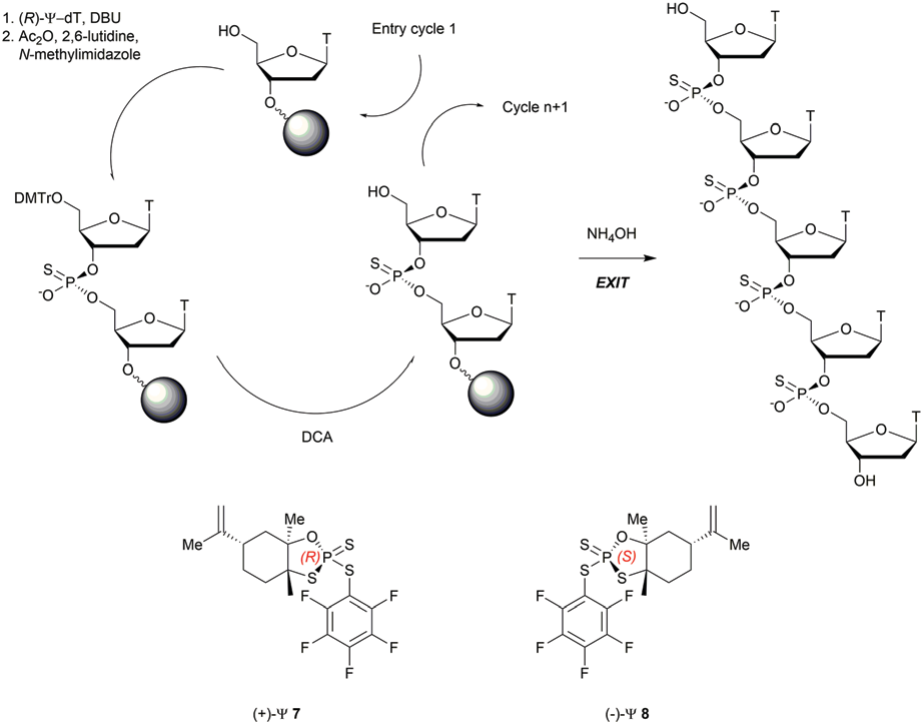
Synthesis cycle of stereoregular all-*S*_P_-PS-ODN using ψ reagent. Structures of (+/−)-ψ reagents **7** and **8**.

Recently, the group of Baran successfully developed an original stereocontrolled synthesis of PS-ODN using a fundamentally different approach through P(v) chemistry.^111^ First, they developed what they called ψ reagents **7** and **8** based on the inexpensive chiral backbone of (+/−)-limonene oxide (Scheme 6). *R*_p_ and *S*_p_-PS-ODN can be easily synthesised using, respectively, (+)-ψ **7** or (−)-ψ **8** in good to excellent yield (76–96%) and with total stereocontrol in MeCN with DBU as an activator. The next nucleoside is readily coupled using the same conditions (70–91% yield). An all-*S*_P_-PS-ODN 5-mer was synthesised using a simple procedure on a solid support as a single diastereoisomer in 23% overall yield with an unoptimized procedure. The advantage of using nonsensitive P(v) intermediates allowed the authors to perform the synthesis without rigorous exclusion of air and water (Scheme 6).

The method has proven to be efficient, inexpensive and easy to implement. More than 50 years after the discovery of phosphorothioates, significant efforts are still devoted to their synthesis. This illustrates all the potential they still present today.

**Scheme 7:**
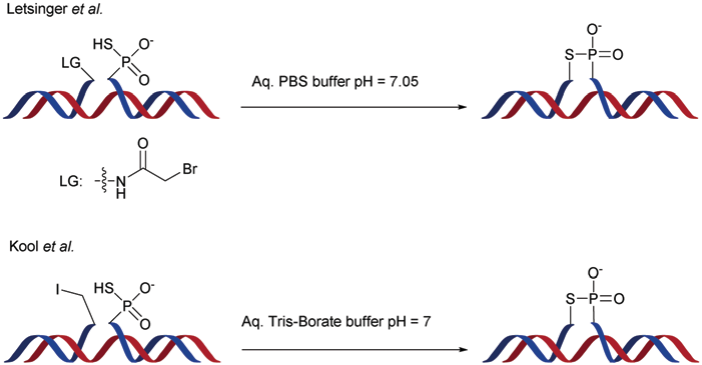
Letsinger^129^ and Kool^130^ templated formation of thiophosphate internucleoside linkages.

Replacing one of the bridging oxygen atoms with a sulphur atom leading to thiophosphates (SP) as phosphorothioate isomers has also been studied.^112–124^ Their main advantage is to avoid the generation of diastereoisomers. However, only a few studies evaluated the resistance to nucleases to determine the potential of the thiophosphate linkage for biological applications. The synthesis strategy is very different from the conventional synthesis strategy for PS obtained by sulfurization of the P(iii) to P(v) during the oxidation step of phosphoramidite chemistry. An example using templated chemical ligation will be detailed in the following. Nucleic acid templated chemical ligation reactions are based on the hybridization of complementary nucleic acid strands, which force the spatial proximity of reactive groups of modified ODN in order to dramatically accelerate a given reaction. Since the pioneering work of Gilham and Orgel,^125,126^ who used a complementary strand to form a phosphodiester linkage under carbodiimide activation of a phosphate group, numerous methods have been described in order to covalently link ODN in aqueous media. Exploiting templated chemical ligation, the group of Letsinger^127^ developed a synthetic method using 5′-phosphorothioate ODN^128^ and another ODN having a bromoacetyl moiety at its 3′ end introduced by reaction of the free alcohol on *N*-succinimidyl bromoacetate^129^ (Scheme 7). The conjugation reaction spontaneously takes place in aqueous media in the presence of the complementary strand. A few years later, Kool^130^ devised a simple method to obtain this modified linkage by employing also two modified half-strands: the first one is modified at the 3′-end with a phosphorothioate obtained during the oxidation step with Beaucage reagent, whereas the second half-strand carries an iodine atom at its 5′ extremity, introduced by treatment of the 5′-free hydroxyl with Moffatt's reagent.^131^ The presence of a template complementary to both half-sequences brings the two functions in close proximity, allowing spontaneous conjugation through the nucleophilic substitution of the halogenated carbon with the sulphur atom, leading to a thiophosphate linkage (Scheme 7).

**Scheme 8:**
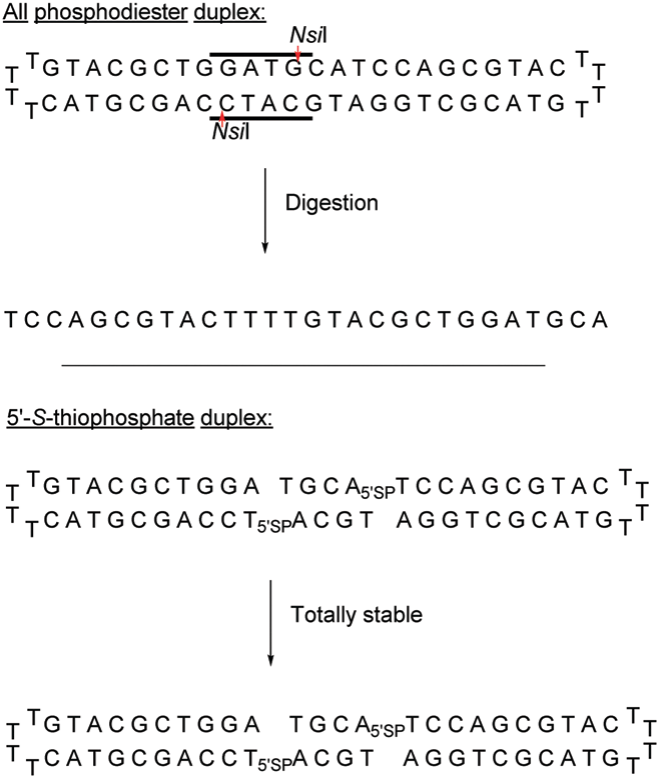
Enzymatic resistance of the 5′-SP linkage to *Nsi*I endonuclease.

This ligation took place between positions 8 and 9 of a 20-mer ODN. The increase in resistance achieved by the SP linkage was evaluated against the T4 DNA polymerase and the SVPDE and CSPDE exonucleases.^130^ With T4 DNA polymerase, the SP connection was five to ten times more resistant than that for the unmodified ODN. By contrast, no resistance was observed against SVPDE, highlighting the high efficiency of this particular nuclease. The modified ODN was then evaluated in the presence of CSPDE. In this case, the kinetics of the degradation of the modified sequence was slower than that of the natural sequence, with a significant “pause” that occurred. Indeed the 3′ hydrolysis took place until the enzyme reached the SP linkage whose hydrolysis was slowed down. The authors hypothesized that the replacement of the oxygen atom with a sulphur atom (which also implies a modification of the binding lengths of about 0.4 Å) deeply modifies the electrostatic interactions within the active site of the enzyme. This interaction reduces significantly the enzymatic kinetics. Indeed, after prolonged incubation time the ODN was completely degraded. Finally, to probe the endonuclease resistance of the SP linkage a modified cyclic ODN was synthesised by double templated self-ligation (Scheme 8).

Both modifications were placed within the 6 bp palindromic sequence ATGCAT, a substrate of the restriction enzyme *Nsi*I. While the natural sequence was totally degraded by the *Nsi*I enzyme after 1.5 hours, the thiophosphate cyclic ODN remained intact. This synthetic strategy was later used to develop a series of ODN bearing a thiophosphate linkage which were inhibitors of the human hepatitis C virus (HCV).^132,133^

Obika's group was interested in the 5′-SP linkage and carried out a complete study of this modification in 2016.^134^ The authors investigated its hybridization properties, its stability against phosphodiesterase I, and the activation of RNase-H and performed an *in vivo* study. The synthesis of SP-ODN relied on the functionalization of 5′-S-DMTr-thymidine^135^ for the implementation of phosphoramidite chemistry. The influence of the 5′-SP linkage on annealing properties was evaluated by hybridization with complementary DNA or RNA strands (Table 6).

**Table 6:** Thermal denaturation studies (*T*_m_ values) of different ODN with complementary DNA or RNA and their half-life evaluations against phosphodiesterase I^134^

ODN (5′ → 3′)^a^	*T*_m_ with DNA^b^ (°C)	*T*_m_ with RNA^b^ (°C)	*t*_1/2_ (min)
d(GCGTTTTTTGCT)	50	45	—
d(GCGTTT_5′SP_TTTGCT)	48	45	—
d(GCGTT_5′SP_T_5′SP_T_5′SP_TTGCT)	44	42	—
d(TTTTTTTTTT)	—	—	2
d(TTTTTTTTT_PS_T)	—	—	>40
d(TTTTTTTTT_5′SP_T)	—	—	8
d(TTTTTTTTT^Me^C)	—	—	7
d(TTTTTTTTT_5′SP_^Me^C)	—	—	35

a PS and 5′SP refer to the phosphorothioate and 5′-SP internucleoside linkages respectively.

b ODN not tested.

The incorporation of the 5′-SP linkage at different positions of the ODN showed acceptable differences in binding with complementary DNA and RNA strands (Δ*T*_m_ ∼ −2 °C per modification with complementary DNA and −1 °C with complementary RNA). Thereafter, the authors studied the resistance to nucleases using phosphodiesterase I. Under the conditions used, the ODN containing 5′-*S*-5-methycytidine was more stable than the ODN containing 5′-*S*-thymidine. As expected, the 5′-SP modified ODN exhibited higher nuclease resistance compared to the unmodified one. However, the PS-ODN tested had better stability than the 5′-SP analogue, demonstrating the lower protection achieved by a thiophosphate linkage compared to a PS one. Finally, different AS-ODN gapmers targeting mouse *Pten* mRNA were synthetized (sequence: 5′TCATGGCTGCAGCT3′). The latter consist of two locked nucleic acid (LNA) nucleosides at each extremity and PS or 5′-SP linkages at the center. *In vitro* studies demonstrated the ability of the 5′-SP linkage to activate RNase-H. Indeed, similar activity was observed for AS-ODN comprising either PS or 5′-PS linkages. However, *in vivo* studies surprisingly gave very different results. Whereas the PS-gapmer induced high activity, the 5′-SP analogue was not active.

Two years later, the same group published a similar study concerning the synthesis of 5′-*S*-thiophosphate-LNA nucleoside analogues of thymidine and 5-methylcytosine.^136^ The aim of this work was to exploit both the enhanced stability in serum and the better binding affinity of LNA nucleoside analogues. The authors studied the annealing properties of the 5′-SP-LNA-ODN having the same sequence as the one previously studied.^134^ The stabilizing effect induced by the LNA modification was observed. Indeed, only the 5′-SP-LNA-ODN bearing three consecutive modifications exhibited the formation of less stable duplexes with its complementary DNA strand (Table 7). Nuclease stability experiments were conducted against SVPDE (Table 7). The half-life of the natural homothymidylate is about 8 min, whereas all the 3′ modified ODN exhibited high stability with half-lives superior to 40 min. The data showed that the 5′-SP-^Me^C-LNA modification provided the best protection against SVPDE hydrolysis but all the ODN tested exhibited stabilities of the same order of magnitude. Further experiments are required to determine the potential of this modification for biological applications.

**Table 7:** Thermal denaturation studies (*T*_m_ values) of different ODN with complementary DNA or RNA and their half-life evaluations against SVPDE^136^

ODN (5′ → 3′)^a^	*T*_m_ with DNA^b^ (°C)	*T*_m_ with RNA^b^ (°C)	*t*_1/2_ (min)
d(GCGTTTTTTGCT)	50	45	—
d(GCGTTT_5′SP_T^L^TTGCT)	52	53	—
d(GCGTT_5′SP_T^L^_5′SP_T^L^_5′SP_T^L^TGCT)	43	50	—
d(GCGT_5′SP_T^L^T_5′SP_T^L^T_5′SP_T^L^GCT)	53	60	—
d(TTTTTTTTT_5′SP_T^L^)	—	—	>40
d(TTTTTTTTT_5′SP_^Me^C^L^)	—	—	>40
d(TTTTTTTTTT)	—	—	8
d(TTTTTTTTT^Me^C)	—	—	>40
d(TTTTTTTTT_PS_T)	—	—	>40

a PS and 5′SP refer to the phosphorothioate and 5′-SP internucleoside linkages respectively. L refers to LNA residues.

b ODN not tested.

**Scheme 9:**
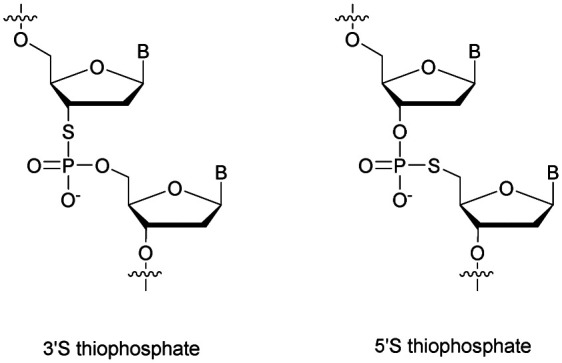
Chemical structures of 3′-SP and 5′-SP linkages.

Recently Duschmalé *et al.* published the chemical synthesis of two series of ODN bearing either a bridging 3′ or a 5′ sulphur atom (Scheme 9).^137^ The authors designed several synthetic pathways to obtain 3′-*S* and 5′-*S*-thiophosphate phosphoramidite building blocks of the four nucleosides in the deoxyribonucleoside series. The synthesis of 3′-*S* nucleoside analogues exploits either the formation of anhydro-pyrimidines (T and C) or the Mitsunobu reaction for purines (A and G).

The 5′-*S*-thiophosphate was obtained from thymidine. After mesylation at the 5′ position, the sulphur atom was introduced upon treatment with DMTrSAc in the presence of NaOMe. Thereafter, 3′-SP and 5′-SP linkages were incorporated within LNA-ODN gapmers at different positions using standard phosphoramidite solid phase oligonucleotide synthesis. The stability of the duplexes formed with their complementary RNA strand was evaluated (Table 8).

**Table 8:** Thermal denaturation studies (*T*_m_ values) of different ODN with complementary RNA and their degradation against mouse liver homogenates^137^

ODN (5′ → 3′)^a^	*T*_m_ with RNA (°C)	Mouse liver homogenates^b^	RNase-H activity^c^	IC_50_ (nM)
d(G^LMe^C^L^ATTGGTATT^LMe^C^L^A^L^)	59.4	—d	5.8	93
d(G^LMe^C^L^A_3′SP_TTGGTATT^LMe^C^L^A^L^)	60.0	68	17.3	8833
d(G^LMe^C^L^A_5′SP_TTGGTATT^LMe^C^L^A^L^)	58.5	56	9.9	410
d(G^LMe^C^L^AT_3′SP_TGGTATT^LMe^C^L^A^L^)	62.5	34	13.6	936
d(G^LMe^C^L^AT_5′SP_TGGTATT^LMe^C^L^A^L^)	57.4	56	11.4	340
d(G^LMe^C^L^ATTGG_3′SP_TATT^LMe^C^L^A^L^)	62.5	1.2	10.0	1345
d(G^LMe^C^L^ATTGG_5′SP_TATT^LMe^C^L^A^L^)	58.5	41	11.3	—d
d(G^LMe^C^L^ATTGGTA_3′SP_TT^LMe^C^L^A^L^)	61.9	1.5	9.8	1129
d(G^LMe^C^L^ATTGGTA_5′SP_TT^LMe^C^L^A^L^)	59.0	31	56.9	—d

a 3′SP and 5′SP refer to the 3′-SP and the 5′-SP linkages respectively. L refers to LNA residues.

b % intact gapmer at the end of the experiment.

c % full length target RNA after 48 h of incubation.

d Gapmer ODN not tested.

A destabilization of 0.5–2.5 °C was observed for the 3′-SP linkage depending on its position. Single 5′-SP modifications turned out to have either no destabilizing effect for some designs or a destabilizing effect of up to −3 °C against the complementary RNA strand. The nuclease resistances of the ODN were evaluated by incubation in diluted mouse liver homogenates for 48 h. The half-lives were not precisely determined; only the relative amount of intact ODN remaining was given (Table 8). Within the 3′-SP linkage series, the best resistance was observed when the modification was placed at the ends of the gap region. Lower resistance was observed when the modification was placed at the center of the gap. Similarly, 5′-SP linkage modifications at the 5′ end of the gap resulted in the best relative stability compared to any of the other 5′-SP modifications. The sequence studied was designed to target Malat1 (metastasis associated lung adenocarcinoma transcript 1),^138^ which is a target for antigen therapies against human lung carcinoma cells. Thus, the authors studied the influence of the thiophosphate linkage on the activity of the RNase-H. *In vitro* studies demonstrated that all the modified 3′-SP and 5′-SP-gapmers were able to successfully and efficiently recruit RNase-H although the non-modified gapmer exhibited the best activity (Table 8). Encouraged by this interesting result, the authors performed *in cellulo* experiments using lung carcinoma cells and determined the IC_50_ values of the different gapmers. Compared to the *in vitro* experiment, the lower activity of the gapmers bearing a SP linkage is surprisingly more pronounced. Finally, *in vivo* experiments demonstrated that the thiophosphate gapmers exhibited only little activity in the kidneys and no activity in the liver, the target organ of this specific sequence. This result may be due to their very different pharmacokinetic properties that could explain the differences between the *in vitro* and *in vivo* experiments. Note that these results confirm the observations made previously by the group of Obika.^134^

As we have seen in this section, the SP linkage exhibits some interesting properties but *in vivo* experiments have not been conclusive so far. In contrast, the PS linkage represents the most exploited modification, including several therapeutic molecules on the market. The easy access to this modification, simply by modifying the oxidation step during the supported synthesis, reinforces the interest of the scientific community. Although the description of this modification dates back to the 1960s, many groups have continued their research efforts, in particular because it tolerates RNase-H activity *in vivo*, an essential property for therapeutic AS applications, while providing increased resistance to nucleases.

The recent use of this modification for the synthesis of a nuclease resistant CDN analogue of GMPc, which has shown very interesting antitumor activity in many models, further highlights the importance of the PS linkage. Indeed, this molecule is today in clinical trial.^139,140^

Finally, it should be mentioned that the discovery of phosphorothioate modifications in bacterial DNA has challenged the current understanding of the phosphodiester backbone of cellular DNA.^141–145^

#### 3.1.2 Phosphoroselenoate (PSe) and selenophosphate (SeP) linkages

By analogy with PS linkages, phosphoroselenoate (PSe) derivatives have also been reported in the literature. Although short dimers or trimers have been described earlier,^77,146^ the first PSe-ODN was synthesised in 1989 by the group of Stein.^147^ They used *H*-phosphonate chemistry with a modified oxidation step to convert the P(iii) into P(v) using potassium selenocyanate as an oxidizing reagent. They were able to study the physico-chemical properties of several PSe-homothymidylates. However, the exchange of selenium by oxygen atoms from PSe-ODN was quantified with a half-life of 30 days in aqueous solutions. This observation greatly reduced their potential for biological applications.

**Fig. 2:**
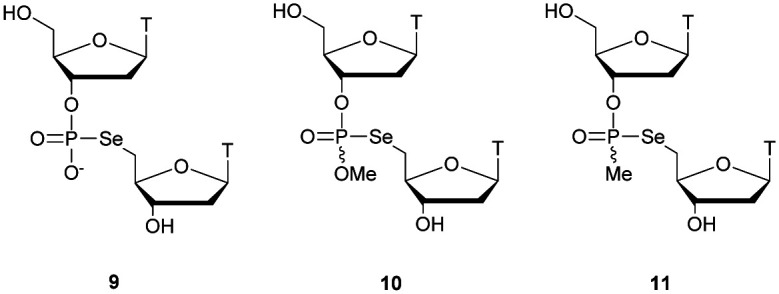
Chemical structures of the first dinucleotide analogues bearing a selenium atom.^148^

Regarding the selenophosphate (SeP) isomer in which a bridging oxygen atom is replaced with a selenium atom (3′ or 5′), only a few studies are available in the literature. The group of Stec described in 1994 the synthesis of *P*-achiral dithymidine selenophosphate **9**, *O*-methyl-phosphoroselenoate **10** and methanephosphonoselenoate **11** (Fig. 2).^148^

Only the dithymidine selenophosphate (**9**) was subjected to nuclease resistance experiments because the dithymidines **10** and **11** decomposed in solution at pH 7.5 within days. The synthesis used as a key step direct oxidation of P(iii) with elemental selenium. The selenophosphate dithymidine (**9**) was incubated with a large excess of SVPDE or nuclease P1 compared to standard protocols in order to achieve digestion. The hydrolysis was performed qualitatively and the authors described a significant increase in resistance compared to the PS dithymidine analogue. During the experiment, the formation of diselenide thymidine (SedT)_2_ was observed as a highly hydrophobic compound. This modification has not been further studied for years with the exception of the use of 3′-SeP-ODN by Kool and co-workers for templated-directed chemical ligation^149^ and a new method developed by Vyle for the synthesis of nucleoside selenophosphates *via* the efficient Michaelis–Arbuzov reaction of selenocyanates.^150^ The authors were able to synthetize a selenophosphate dimer with high efficiency.

**Scheme 10:**
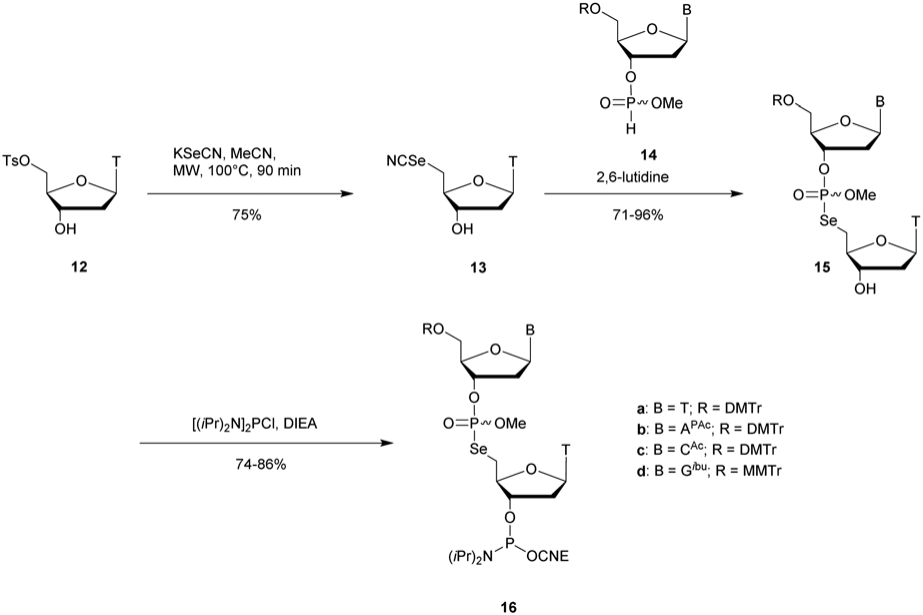
Synthesis of phosphoroselenoate building blocks **16a–d**.^151^

Recently Conlon *et al.* described the first solid-phase synthesis of phosphoroselenoate-ODN.^151^ They exploited the work of Vyle to synthetize dinucleoside phosphoroselenoate triesters and upon subsequent phosphitylation introduced them into ODN. First, 5′-tosylthymidine **12** was converted into the corresponding 5′-selenocyanate **13** within 90 min under microwave irradiation. 3′-*H*-Phosphonate derivatives **14** were prepared from the corresponding phosphoramidites using previously described conditions,^152^ and then coupled with the 5′-selenocyanate **13** in MeCN in the presence of 2,6-lutidine. Finally, dimers **15a–d** were converted into the corresponding phosphoramidite building blocks **16a–d** (Scheme 10).

The phosphoramidite dimers **16a–d** obtained allowed the use of classical supported synthesis protocols. The authors synthetized various ODN designed to adopt an A-form conformation, comprising a single SeP linkage at their 5′ end. Thermal denaturation studies were performed and showed a sequence-dependent destabilization of the duplexes formed with their complementary DNA strand (Δ*T*_m_ from −0.7 to −6.2 °C per modification). The decreases in melting temperatures were all the more significant as the native ODN were stable (presence of a C_SeP_G or G_SeP_C base pair at the 5′ extremity with Δ*T*_m_ of −6.2 °C or −4.9 °C respectively). By contrast, a minor effect was observed for a 5′ terminal A_SeP_T base pair (Δ*T*_m_ −0.7 °C) although the effect was more important for a T_SeP_A base pair (Δ*T*_m_ −4 °C). Qualitative enzymatic digestion was performed with SVPDE on the ODN d(T_SeP_TCCCGGGAA) and the formation of diselenide thymidine (SedT)_2_ was observed as the group of Stec did.^148^ The authors assumed that the low nuclease activity was due to the distortion in the phosphoryl moiety of the SeP linkage that limits nuclease recognition. The increased resistance observed for SeP–ODN offers potential for *in vivo* applications. Concerning AS therapy, RNase-H activation study remains to be done.

#### 3.1.3 Phosphoramidate (NP) linkage

A phosphoramidate (NP) linkage is synthesized by replacing an oxygen atom with a nitrogen atom. It was described for the first time by Jastorff *et al.* in 1969.^153^ The authors performed the synthesis of dinucleotides having a bridging nitrogen atom at the 5′ position. They observed that the linkage was sensitive to acidic pH which causes rapid hydrolysis.

**Fig. 3:**
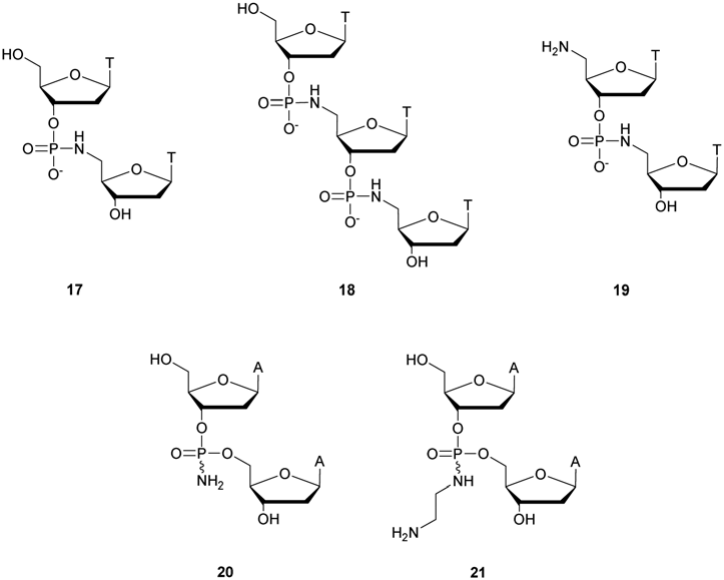
Chemical structures of phosphoramidates **17**, **18** and **19** and NP modified diadenosines **20** and **21** studied by Letsinger *et al.*^154,155^

Thereafter, Letsinger *et al.*^154^ relied on this work and went further by synthesizing di- and trinucleotides (Fig. 3) in order to evaluate the resistance to nucleases of the NP linkage. The authors tested SVPDE and CSPDE on **17** and **18** at first. Both nuclease activities were reduced on phosphoramidate substrates compared to natural ones. This decrease in activity has not been precisely quantified, but is about 10–20% based on the raw data. However, in the case of CSPDE dimer **17** was converted to thymidine and 5′-phosphoramidate thymidine. In the case of **18**, a large amount of dinucleotide **19** was obtained, suggesting that the presence of the 5′-NH_2_ group significantly inhibits the CSPDE activity. In order to confirm this assumption, compound **19** was synthesised. The enzymatic tests performed on dimer **19** confirmed the hypothesis that the CSPDE, unlike the SVPDE, is particularly sensitive to this modification.

Letsinger *et al.* also studied the behavior of non-bridging phosphoramidates.^155^ They synthesised two dinucleotides d(A_NP_A) **20** and **21** (Fig. 3) and evaluated their resistance to SVPDE and CSPDE.

The half-lives of the dinucleotides were not determined. However, nuclease resistance studies were performed by incubation in the presence of SVPDE or CSPDE for 16 h. While the natural dinucleotide is fully hydrolysed, amino-NP dinucleotide **20** is hydrolysed only up to 14% by SVPDE and 8% by CSPDE. Aminoethyl-NP dinucleotide **21** is completely stable during the experiment.

**Scheme 11:**
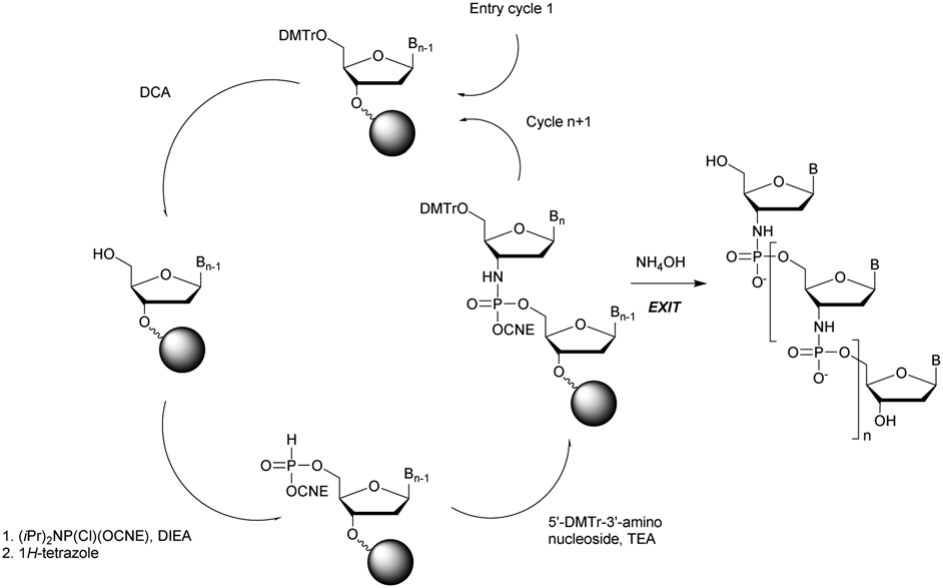
Synthesis cycle of N3′ → P5′ PN-ODN.

In 1994 Gryaznov *et al.* published a method to synthesize on a solid support N3′ → P5′ NP-ODN using a standard controlled pore glass (CPG) support and modified *H*-phosphonate chemistry (Scheme 11).^156^

After removal of the DMTr protective group, the solid support is treated with 2-cyanoethyl *N*,*N*-diisopropylchlorophosphoramidite followed by 1*H*-tetrazole and water to generate a 5′-*H*-phosphonate function. An Atherton–Todd type reaction is then performed with a 3′-amino nucleoside to obtain the corresponding N3′ → P5′ phosphoramidate. This cycle can be repeated for NP-ODN elongation with an average yield of 94–96% per cycle. The synthesis ends with a classical aqueous ammonia final deprotection. The authors then described the hybridization properties of their NP-ODN. The conclusions obtained are that the NP-ODN/ODN duplexes are more stable than their natural counterparts. In addition, NP-ODN are capable of forming very stable triplexes with complementary ORN. This is probably due to the substitution of the 3′ oxygen with a nitrogen atom, which changes the conformation of the ORN 2′-hydroxyl and may favour inter-base hydrogen binding. Additional stabilization is obtained because the phosphoramidate is relatively more rigid than the phosphodiester linkage.

The RNase-H activation of N3′ → P5′ induced by phosphoramidate ODN was assessed by the groups of Gryaznov^157^ and Nerenberg^158^ in order to evaluate their potential for antisense applications. The latter were tested to target the mRNA coding for the Tax protein, a major transcription factor of leukemia type I virus targeting human T cells. All experiments were performed with four ODN 15-mers with the same sequence (as well as several control ODN) with PO, NP, chimeric PO/NP and PS linkages. The first step was to evaluate the resistance of NP-ODN to nucleases to validate the use of NP-ODN for antisense applications. Thus, they were exposed to extracts of cell nuclei. Results showed a total degradation of the PO-ODN within 5 min. In contrast, NP-ODN remained stable after 1 hour of incubation and chimeric PA/PO-ODN were still present, although partially degraded. Finally, the PS-ODN still underwent partial degradation, probably due to 3′-exonuclease activity. These experiments confirmed the high resistance of PS-ODN, which has been demonstrated several times in the literature, but also highlighted the potential of NP-ODN for the antisense strategy thanks to their significant resistance to nucleases.

The authors then performed *in cellulo* experiments to inhibit the translation of Tax protein. Unexpected results were obtained with sequence-dependent inhibition by a different mechanism than activation of RNase-H. Indeed, under similar conditions, whereas no significant inhibition was observed with the PO-ODN, PS-ODN or PN/PO-ODN, 70% reduction of the amount of Tax protein was observed with PN-ODN. A surprising reduction was also obtained with PN-ODN comprising a mismatch. This seems to indicate a mechanism of inhibition by disruption with the RNA at the level of its production or its transport or during its translation by steric blocking. Steric blocking ODN block access of cellular machinery to mRNA, preventing the translation process from occurring, without degrading the RNA. Indeed, it has previously been shown that the *T*_m_ values of NP-ODN increased by about 1.2 °C per residue compared to PO-ODN.^159^

**Fig. 4:**
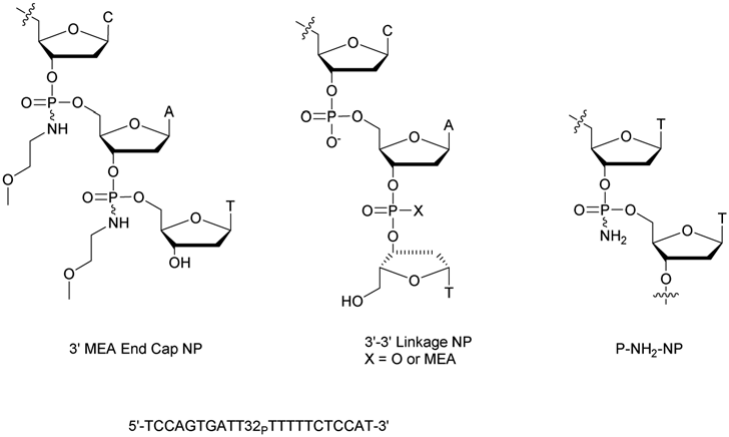
Oligonucleotides studied by Shaw *et al.*^160^ and chemical structure of the P-NH_2_-NP linkage.^161^

Shaw *et al.* published a study concerning the determination of the deoxyribonuclease profile for FCS and human serum.^160^ For this purpose they used different ^32^P-labelled 21-mer ODN for analytical monitoring. The structures studied are PO-ODN, PS-ODN and two chimeric NP/PO-ODN possessing at either the 3′- or 5′-end one or two phosphoramidate linkages having a non-bridging methoxyethylamino (MEA). Finally, two ODN having a PO or NP 3′–3′ terminal linkage were also studied (Fig. 4).

The half-lives of the different ODN were determined both in FCS and in human serum (Table 9).

**Table 9:** Half-life evaluations of different ODN in FCS and human serum^160^

ODN (5′ → 3′)^a^	*t*_1/2_	
	FCS	Human serum
d(TCCAGTGATTTTTTTCTCCAT)	<5 min	∼3 h
d(T_PS_C_PS_C_PS_A_PS_G_PS_T_PS_G_PS_A_PS_T_PS_T_PS_-T_PS_T_PS_T_PS_T_PS_T_PS_C_PS_T_PS_C_PS_C_PS_A_PS_T)	∼4 h	>7 d
d(TCCAGTGATTTTTTTCTCC_NP_A_NP_T)	∼4 h	>7 d
d(T_NP_C_NP_CAGTGATTTTTTTCTCCAT)	<5 min	∼3 h
d(TCCAGTGATTTTTTTCTCCA_3′-3′PO_T	∼4 h	>7 d
d(TCCAGTGATTTTTTTCTCCA_3′-3′NP_T	∼4 h	>7 d

a PS and NP refer to the phosphorothioate and MEA phosphoramidate internucleoside linkages respectively.

Clearly, the nuclease digestion was faster in FCS than in human serum. This difference was constant for all the ODN studied. The PO-ODN and 5′-NP-ODN were both rapidly degraded, indicating a similar pattern of degradation over time. Unlike 5′-NP-ODN, the 3′-NP-ODN had a much better resistance. When the 3′ terminal linkage was reversed, resulting in a 3′–3′-dinucleotide (whether a PO or an NP bond), the stabilization obtained in the two sera was similar to the one obtained with the 3′-NP. The conclusion was that the predominant nuclease activity in the two sera tested was 3′-exonuclease.

In an effort to understand the role played by *N*-alkyl chains in phosphoramidates, our group synthesised P-NH_2_ derivatives (Fig. 4) using either *H*-phosphonate or phosphoramidite chemistry.^161^ Both mixed and uniformly modified phosphoramidate/phosphodiester dodecamers were synthesised on solid supports using a procedure previously described to oxidize the phosphorus atom of an *H*-phosphonate diester linkage with an amine using a saturated solution of ammonia in a mixture of dioxane and CCl_4_.^162^ Various homothymidylate ODN differing by the number and the positioning of the modifications were produced. Two particular sequences were evaluated against degradation with nuclease S1, CSPDE and SVPDE by comparison with the PO-ODN (Table 10).

**Table 10:** Half-life evaluations of NP-ODN against nuclease S1, CSPDE and SVPDE^161^

ODN (5′ → 3′)^a^	*t*_1/2_			RNase-H activation
	Nuclease S1	CSPDE	SVPDE	
d[(T)_11_T]	7 min	22 min	14 min	✓
d[T_NP_(T)_5_T_NP_T]	20.7 h	26 h	9 h	✗
d[(T_NP_)_3_(T)_5_-(T_NP_)_3_T]	7 min	12 d	8.5 h	✓

a NP refers to the P-NH_2_ phosphoramidate internucleoside linkage.

The presence of phosphoramidate linkages drastically increased the resistance of the ODN to the three nucleases tested. The only exception was the rapid hydrolysis of the ODN consisting of five PO units flanked with three NP units. Indeed nuclease S1 is an endonuclease and the five natural nucleotides present at the center of the sequence allow its activation. In addition, these ODN were also tested for their ability to activate RNase-H for antisense applications. Only the chimeric ODN with a central phosphodiester section was able to activate RNase-H hydrolysis (Table 10) in agreement with previous work concerning chimeric methylphosphonate (MP)/PO-ODN (MP modification will be reviewed in the next Section 2.1.4.1).^163,164^ It should be noted that these non-bridging NP bonds form less stable duplexes with DNA targets (Δ*T*_m_ ∼ −1.2 °C per modification) than the corresponding phosphodiesters.

**Fig. 5:**
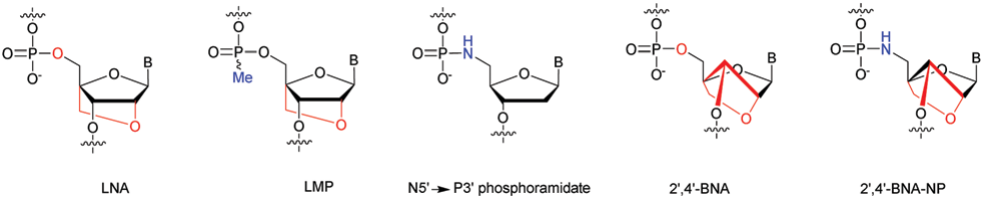
Chemical structures of modified ODN: LNA, LMP, N5′ → P3′ PN, 2′,4′-BNA and 2′,4′-BNA-NP.

In 2001, Imanishi and co-workers exploited the physico-chemical properties of LNA modified carbohydrates^165^ in the context of the NP internucleoside linkage. They developed the synthesis of N5′ → P3′ 5′-amino-2′-*O*,4′-*C*-methylene bridged nucleic acid (2′,4′-BNA-NP, Fig. 5).^166,167^

The synthesis of the 5′-DMTr-amino-2′-*O*,4′-*C*-methylene bridged phosphoramidite building block derived from thymidine was carried out with 60% overall yield in 9 steps. The hybridization studies of 2′,4′-BNA and 2′,4′-BNA-NP modified ODN showed a significant increase in stability. Indeed, duplexes formed with their complementary ODN or ORN strand showed stabilizations between +3 and +7 °C per modification. Concerning the formation of triplexes with double stranded ODN, the stabilization is increased up to 10 °C per modification. Thereafter, the resistance of 3′ modified ODN was evaluated against the SVPDE. The degradation was followed by HPLC analysis. The natural PO-ODN was fully hydrolysed within 5 min. The resistance to SVPDE of 2′,4′-BNA and 2′,4′-BNA-NP modified ODN is greatly improved with respective *t*_1/2_ of 15 and 40 respectively.

In the context of double modification, our group developed in 1990 the synthesis of α-anomeric-ODN.^168^ We demonstrated that an α-r(U_6_) was totally resistant to CSPDE, nuclease S1 and ribonuclease A. Moreover, a significantly enhanced resistance was observed to SVPDE. Years later, we demonstrated that α-PN-ODN hybridized to their complementary RNA strand were unable to activate RNase-H.^169^

Noteworthily, the study of the NP linkage also opened the way to the elaboration of useful doubly modified linkages that are reviewed in another section of this review (see Section 2.1.8).

#### 3.1.4 Carbophosphonate linkage

##### 3.1.4.1 Methyl (MP) and phenyl phosphonate (PhP) functionalization

**Scheme 12:**
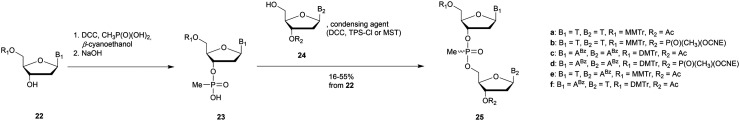
General route for the synthesis of MP-dinucleotides **25a–f**.

In 1977 Miller *et al.* presented for the first time at a meeting the synthesis of several methyl phosphonate (MP) modified dinucleotides in moderate yields (16–38%).^170^ The corresponding publication was available two years later.^171^ Although the synthesis of 3′-methylphosphonate cyanoethyl is satisfactory using dicyclohexylcarbodiimide (DCC) as an activating agent, the condensation leading to the dimers required the use of mesitylenesulfonyl tetrazole (MST) to achieve better results than DCC or triisopropylbenzenesulfonyl chloride (TPS-Cl, Scheme 12). Note that the synthesis of these dimers led to the formation of diastereoisomeric mixtures **25a–f**.

In 1979, Agarwal *et al.* improved the condensation step yield to 60–70% through the use of benzenesulfonyl tetrazole as a condensing agent.^172,173^ The authors studied the nuclease resistance of dithymidine methyl- and phenyl-phosphonate (PhP) to spleen phosphodiesterase and SVPDE (Table 11). MP and PhP linkages were totally resistant to spleen phosphodiesterase.

**Table 11:** Half-life evaluations of MP- and PhP-ODN against SVPDE^172^

ODN (5′ → 3′)^a^	*t*_1/2_
d(TT)	10 min
d(T_MP_T)	24 h
d(T_PhP_T)	>24 h
d(TT_PhP_TT)	10 min
d(T_PhP_TT)	24 h

a MP and PhP refer to the methyl- and phenyl-phosphonate internucleoside linkages respectively.

The MP linkage is very resistant compared to the natural one (*t*_1/2_ = 24 h *versus* 10 min). The PhP bond is even stronger, probably due to its larger steric hindrance. It was observed that only 50% of the starting modified dimer was hydrolysed, even after further addition of the enzyme. This indicates that the nuclease can only hydrolyse one of the two diastereoisomers present. Interestingly, the hydrolysis of the PO dimer was slowed down by the presence of the MP analogue, probably due to the slow dissociation of the latter from the active site of the enzyme due to its neutral charge. Finally, it was shown that the hydrolysis of the first nucleotide of the tetramer d(TT_MP_TT) is very fast, while for the second (the MP linkage) the rate of hydrolysis is drastically reduced. These data indicate that the SVPDE is not affected by the presence of a MP linkage next to its PO target.

In 1987 Agrawal *et al.* developed a solid supported synthesis of MP-ODN using nucleoside dithymidine methylphosphonamidite as starting materials.^174^ The latter were obtained by reacting 5′-*O*-DMTr-thymidine with methylchloro-*N*,*N*-diisopropylaminophosphine. Note that this strategy was also applied to obtain protected adenosine, cytidine and guanosine derivatives. Different modified MP-ODN 7-mers were then synthesised using classical solid supported ODN chemistry and incubated with SVPDE.

The results obtained showed that the d(TTTT_MP_TTT) ODN gave a T_MP_T fragment as the product. This result indicates that the SVPDE is able to “jump” over a single MP linkage to continue its activity. It is important to emphasize that this ability is rare among the nucleases. This illustrates that the SVPDE is one of the most effective nucleases and explains why it is often used to evaluate the resistance of modified internucleoside linkages. Thus, an increase of the number of consecutive MP linkages induced a 200 fold resistance increase compared to the unmodified ODN. Similar results have been obtained with CSPDE. Thus, a good protection against both enzymes is obtained by introducing at the extremities of the ODN two consecutive MP linkages.

The nuclease resistance of MP-ODN was also evaluated by the group of Wetmur,^175^ which confirmed the high resistance of the MP linkage to both SVPDE and CSPDE exonucleases. In addition, the MP-ODN studied were also found to be resistant to DNase I and DNase II endonucleases (Table 12). Resistance to CSPDE and SVPDE of MP-ODN is very important with half-lives multiplied by factors of 200 and 500, respectively, by comparison with the control ODN. Concerning endonucleases, the half-lives of the control ODN are very different (10 min and 10 h for DNases I and II, respectively). The observed increase in resistance to digestion logically depends on the presence of a PO span within the ODN sequence. The first ODN has indeed a continuity of five PO linkages, whereas the second has alternating PO and MP linkages. Thus, the increase in resistance of the second ODN is more important. RNase-H activation tests were also performed to evaluate these MP-ODN in an antisense strategy. Too many MP linkages prevent recognition of the duplexes by RNase-H. In order to have an effective hydrolytic activity, it is necessary to incorporate at the center of the ODN at least 3 consecutive PO linkages. However, even without activation of RNase-H, MP-ODN are able to block the ribosomal machinery thanks to the formation of stable duplexes with the target.

**Table 12:** Half-life evaluations of MP-ODN against CSPDE and SVPDE exonucleases, and DNase I and DNase II endonucleases^175^

ODN (5′ → 3′)^a^	*t*_1/2_			
	CSPDE	SVPDE	DNase I	DNase II (h)
d(TGACTTAGCTGCAT)	30 min	12 min	10 min	10
d(TGAC_MP_TTAGC_MP_TGC_MP_AT)	>100 h	>100 h	60 min	20
d(TG_MP_AC_MP_TT_MP_AG_MP_CT_MP_GC_MP_AT)	>100 h	>100 h	>100 h	>50

a MP refers to a methylphosphonate internucleoside linkage.

In 2003, Wengel decided to study doubly-modified ODN comprising a LNA modification^165^ at the deoxyribose ring and a MP linkage to create LMP-ODN (Fig. 5).^176^ The objective was to obtain an additive effect and increase both the binding and the nuclease resistance of the resulting LMP-ODN. Phosphoramidite building blocks were synthesised by phosphitylation of LNA nucleosides using bis(diisopropylamino)methyl phosphine in the presence of 1*H*-tetrazole. Three different ODN were synthesised and subjected to SVPDE hydrolysis (PO-ODN, LNA- and LMP-modified ODN, Table 13). The unmodified oligonucleotide was rapidly and fully degraded with a half-life of less than 2 min. Only mononucleotides were observed after 10 min. The same experiment reproduced on a LNA-ODN having only one LNA modification showed a moderate increase in resistance as soon as the enzyme reached the modified nucleoside (5 min compared to a few seconds). However, complete hydrolysis was achieved rapidly within 10 min. In the case of LMP-ODN, SVPDE was unable to hydrolyse the modified nucleoside and a total resistance was observed when the enzyme reached the modified linkage. An additional experiment was performed with 25 times more SVPDE, and after 120 min, no further degradation was observed, demonstrating the total resistance of LMP against SVPDE.

**Table 13:** Thermal denaturation studies (*T*_m_ values) of chimeric ODN with combination of LNA and MP linkages with complementary DNA or RNA and their half-lives towards induced SVPDE hydrolysis (once the modified nucleoside was reached)^177^

ODN (5′ → 3′)^a^	*T*_m_ with DNA (°C)	*T*_m_ with RNA (°C)	*t*_1/2_ (min)
d(GTGATATGC)	29	27	<2
d(GT^L^GAT^L^AT^L^GC)	44	53	<5
d(GTG_MP_ATA_MP_TGC)	27	22	30
d(GT^L^G_MP_AT^L^A_MP_T^L^GC)	39	47	>60

a L and MP refer to LNA residues and methylphosphonate internucleoside linkages respectively.

Wengel *et al.* continued their work with the synthesis of heteropolymeric sequences comprising mixed MP and PO linkages as well as the use of LNA nucleosides.^177^ The objective was to study the potential of such modified ODN for antisense applications. The thermal denaturation studies of the duplexes formed with their complementary strand (DNA or RNA) showed a destabilization of the duplexes due to the MP linkages. However, modified LNA nucleosides significantly increased the affinity of the ODN studied. Regarding the chimeric MP/PO/LNA-ODN, the deleterious effect of the MP bond on hybridization was largely compensated for by LNA residues (Table 13). Then, the resistance of these ODN against SVPDE activity was studied. Surprisingly, in this specific work the LNA-ODN were rapidly degraded although the resistance of LNA-ODN to the 3′-exonucleases was reported in the literature.^178,179^ The MP linkage provides a significant increase in resistance to SVPDE compared to the natural PO with a half-life of 30 min. The chimeric MP/PO/LNA-ODN demonstrated remarkable stability with a *t*_1/2_ of more than 60 min, demonstrating the potential of these ODN for antisense applications (Table 13).

No studies concerning the use of these modified ODN as therapeutic tools have been performed so far.

In 1989 Tidd *et al.* published results concerning the protection of antisense ODN against degradation using terminal MP linkages.^180^ They worked on numerous sequences targeting the human oncogene N-*ras* sequence. The resistance of these ODN to SVPDE, CIAP and FCS was assessed using HPLC analysis monitoring (Table 14).

**Table 14:** Half-life evaluations of MP-ODN against SVPDE^180^

ODN (5′ → 3′)^a^	*t*_1/2_	
	SVPDE + CIAP (min)	FCS
d(CAGTTTGT-ACTCAGTC)	<5	30 min
d(CAGTTTGT-ACTCAGTC_MP_A_MP_T)	>180	12 h
d(A_MP_C_MP_CAGTTTGT-ACTCAGTC_MP_A_MP_T)	>180	4 h

a MP refers to a methylphosphonate internucleoside linkage.

**Fig. 6:**
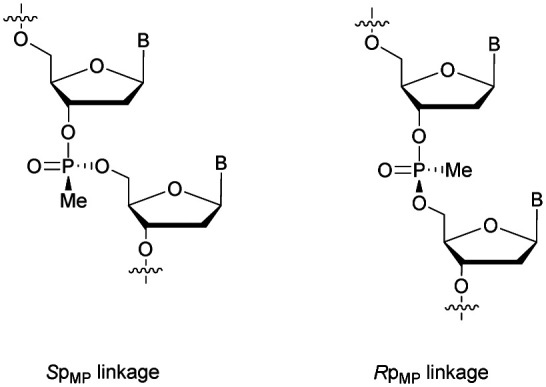
Chemical structures of *S*_p_ and *R*_p_ methylphosphonate chiral linkages.

Modified ODN with 3′-MP internucleoside linkages were found to be stable against SVPDE and CIAP during the course of the experiment. Experiments with FCS showed that MP-ODN have a higher resistance to hydrolysis than PO-ODN. Interestingly, the ODN with MP linkages at its 5′- and 3′-ends is less resistant than the corresponding 3′-MP analogue. The authors mentioned the possibility for the two chimeric ODN to adopt different conformations in solution, slowing down the activity of nucleases in the case of the 3′ modified ODN. As PS-ODN, MP-ODN have an asymmetric phosphorus atom. Several synthesis methods of MP-ODN with controlled stereochemistry have been published and reviewed.^181^ Different strategies have been implemented, such as the separation of the diastereoisomers formed^182,183^ or the stereocontrolled synthesis of the internucleoside linkage (Fig. 6).^184,185^ Thermal denaturation studies have shown that systematically the *R*_p_ stereochemistry of MP internucleoside linkages allows the formation of much more stable duplexes than their *S*p counterparts.

**Fig. 7:**
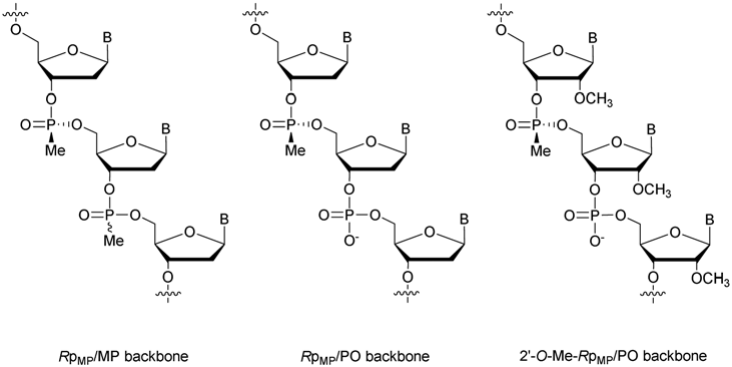
Chemical structures of backbone-modified oligonucleotides containing chiral *R*_pMP_ linkages: *R*_pMP_/MP, *R*_pMP_/PO and 2′-*O*-methyl-*R*_pMP_/PO backbones.

Concerning the resistance to nucleases, Reynolds *et al.* studied the behaviour of the 15-mer (CT)_7_A having different modified structures: alternating *R*_pMP_/MP, *R*_pMP_/PO and 2′-*O*-methyl-*R*_pMP_/PO backbones (Fig. 7). They observed that the presence of the 2′-*O*-Me group also increases the stability of the duplexes formed with their complementary RNA. Thus, four of the ODN studied were tested against five different biological media containing nucleases (Table 15).^186^

**Table 15:** Half-life evaluations of (CT)_7_A ODN or [(CU)_7_A)]-all-2′-*O*-Me ORN against five different media^186^

Medium	*t*_1/2_			
	PO^a^ (min)	2′-*O*-Me RNA^a^ (min)	2′-Deoxy alternating MP-PO^a^ (h)	2′-*O*-Me alternating MP-PO^a^ (h)
10% FBS	12	40	5	>5
COS-7 cell lysate, pH 6.0	<10	300	25	>24
COS-7 cell lysate, pH 7.4	<5	300	20	>24
*E. coli* cell lysate	13	72	65	>24
*S. aureus* cell lysate	15	1200	75	>24

a MP and PO refer to methylphosphonate and phosphodiester internucleoside linkages respectively. 2′-*O*-Me refers to the 2′-*O*-methyl modification of the deoxyribose.

The half-lives of the natural PO-ODN were about ten minutes in all sera. The introduction of the 2′-*O*-Me group significantly increased the resistance of ODN, with their half-lives ranging from one to several hours. In addition, the skeleton constituted by alternating MP and PO linkages presents half-lives of several tens of hours. Finally, the combinations of these two modifications have led to ODN totally stable against nucleases for days. These results demonstrate the potential of this particular modification for biological use due to the high nuclease resistance and low destabilization of the duplexes formed with their complementary strands.

Recently, the group of Holliger published a very interesting study exploiting methyl and ethyl functionalization.^187^ They described the DNA-templated synthesis of methyl and ethyl phosphonodiester polymers using engineered polymerases able to assemble *P*-alkyl-dNTP. However, due to their hydrophobicity, MP-ODN have low water solubility and are likely to be trapped within an endosomal/lysosomal compartment and consequently unavailable for biological activity in the cytoplasm as mentioned by Shoji *et al.*^188^

##### 3.1.4.2 Pyridylphosphonate (PyrP) functionalization

**Scheme 13:**
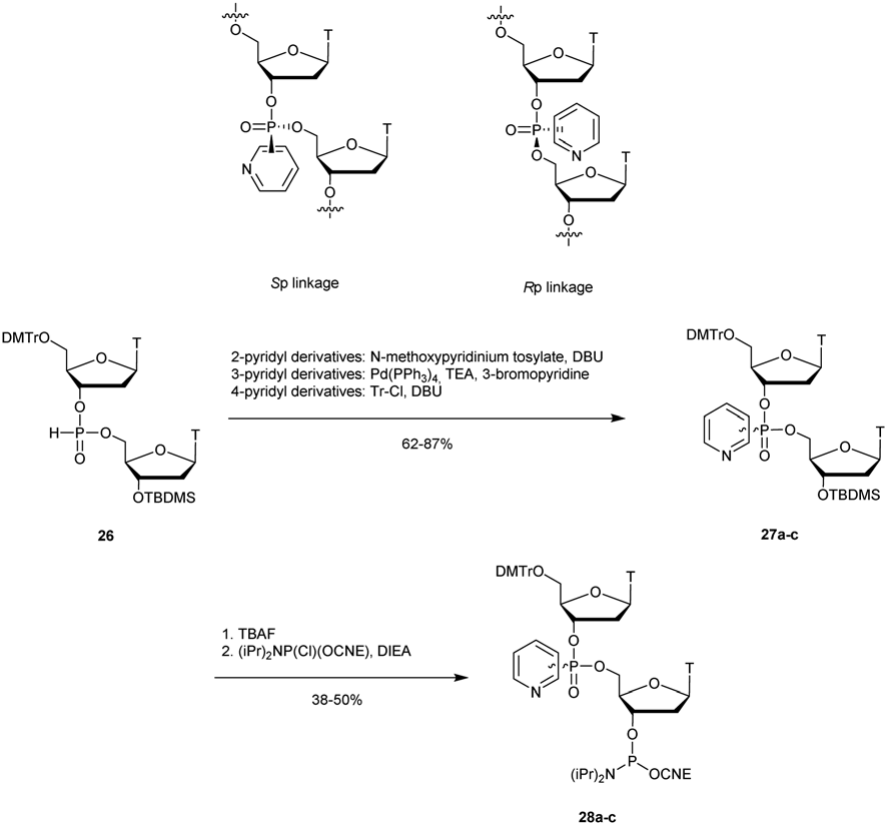
Chemical structures of *S*_p_ and *R*_p_ PyrP chiral linkages. Synthesis of PyrP phosphoramidite building blocks **28a–c**.

In 2003, Zmudzka *et al.* published the synthesis of 2-pyridyl-, 3-pyridyl- and 4-pyridylphosphonate (PyrP) linkages using *H*-phosphonate chemistry.^189^ Dimers **27a–c** ware synthesised conventionally in solution with an intermediate internucleoside *H*-phosphonate linkage. The mixture of the two diastereoisomers generated can be separated by chromatography on silica gel and then functionalized to give the phosphoramidites **28a–c** (Scheme 13).

The stabilities of the duplexes formed between the modified ODN and their complementary DNA or RNA strands were evaluated. The first observation made by the authors was that the replacement of the native phosphodiester with the *P*-chiral 2-, 3- or 4-pyridylphosphonodiester linkage within ODN sequences did not induce pronounced geometric alterations of the resulting duplexes in the case of the *R*_p_ isomer. However, *S*_p_-pyridylphosphonate significantly destabilized double-helical structures (up to −4.9 °C per modification). In order to evaluate the resistance that a pyridylphosphonate linkage provides compared to the natural one, a 2-*R*_p_-pyridylphosphonate ODN modified between residues 10 and 11 was incubated in human plasma or in aqueous buffer in the presence of SVPDE or CSPDE. Since the modification was located at the center of the modified ODN, the enzymatic hydrolysis initially progressed for PyrP-ODN in a similar manner to that for PO-ODN. However, total resistance of the 2-*R*p-pyridylphosphonate linkage was observed regardless of the tested exonuclease over 8 h. Although the properties of pyridylphosphonate internucleoside linkage appeared to be interesting for antisense applications, their ability to elicit RNase H activity has still to be evaluated.

##### 3.1.4.3 Aminomethyl (AMP) and aminoethyl phosphonate (AEP) functionalization

**Scheme 14:**
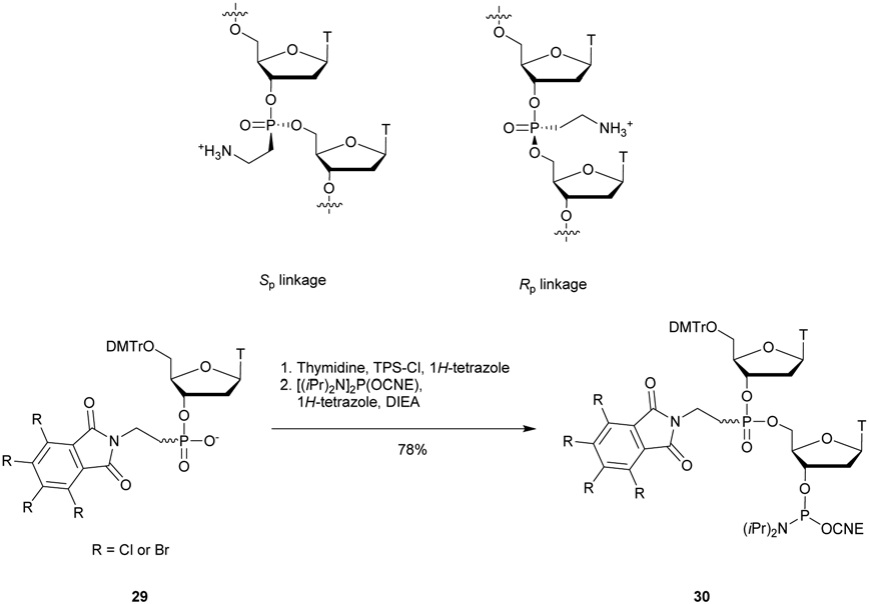
Chemical structures of *S*_p_ and *R*_p_ 2-AEP chiral linkages. Synthesis of the protected 2-(3,4,5,6-tetrabromophthalimido) or 2-(3,4,5,6-tetrachlorophthalimido) phosphoramidite building block **30**.

In 1993, the group of Cook published results concerning the synthesis and the characterization of cationic modified (2-aminomethyl)phosphonate ODN (2-AMP-ODN). One of the objectives was to develop positively charged ODN in order to increase their ability to penetrate cells. 2-AMP-ODN exhibited interesting properties such as nuclease resistance or the ability to form stable duplexes with their complementary strand (for the *R*_p_ isomer). The main drawback of these modified ODN was their spontaneous hydrolysis in aqueous media, preventing their use for biological applications.^190^ A year later, the same group published further results concerning stable (2-aminoethyl)phosphonates ODN (2-AEP-ODN).^191^ The dimers were synthesised in solution with 2-(3,4,5,6-tetrabromophthalimido) or 2-(3,4,5,6-tetrachlorophthalimido)ethylphosphonate internucleoside linkages as mixtures of two diastereoisomers, **29**, which were separated by chromatography on silica gel and then functionalized to obtain phosphoramidites **30** (Scheme 14).

Modified ODN were then synthesised using classical phosphoramidite chemistry with the exception of an additional treatment of the solid supported ODN with ethylene diamine at 55 °C for 30 min in order to remove the phthaloyl group. Then, modified chimeric homothymidylate 13-mers having 6 alternate stereochemically pure (aminoethyl)phosphonate linkages were evaluated for their thermal stabilities in the presence of complementary DNA or RNA strands. Thereafter, their nuclease resistances were quantified against nuclease S1 (Table 16).

**Table 16:** Thermal denaturation studies (*T*_m_ values) of the chimeric *R*_p_ and *S*_p_-AEP/PO-ODN used with complementary single-stranded DNA or RNA and their half-life evaluations against nuclease S1^191^

ODN (5′ → 3′)^a^	*T*_m_ with DNA (°C)	*T*_m_ with RNA (°C)	*t*_1/2_	RNase-H activation
			Nuclease S1	
d(T_13_)	34	30	4 min	✓
[d(T_AEP_TT_AEP_TT_AEP_-TT_AEP_TT_AEP_TT_AEP_TT)]-all-*R*_p_	51	35	>24 h	✗
[d(T_AEP_TT_AEP_TT_AEP_TT_AEP_TT_AEP_TT_AEP_TT)]-all-*S*_p_	12	—	>24 h	✗

a AEP refers to a (2-aminomethyl)phosphonate internucleoside linkage.

The thermal stability of chimeric all-*R*_p_-AEP/PO-ODN with complementary DNA was higher than that of its natural counterpart, whereas the all-*S*_p_-AEP/PO-ODN did not form a stable duplex under physiological conditions. The results obtained with the complementary RNA followed the same destabilization as that observed with a DNA strand. The duplex with the all-*S*_p_-AEP/PO-ODN could not be observed and the one with the all-*R*_p_-AEP/PO-ODN was less stable. Both AEP/PO-ODN are resistant to nuclease S1. While the natural ODN has a *t*_1/2_ of 4 min, the all-*S*_p_-AEP/PO-ODN is totally stable and the all-*R*_p_-AEP/PO-ODN shows only 6% degradation after 24 h of incubation. Despite their interesting hybridization properties and S1 nuclease resistance results, the antisense therapeutic potential of AEP-ODN is limited due to the inability of the AEP modification to activate RNase-H.

##### 3.1.4.4 Methylene functionalization [deoxy-3′-*C*-(hydroxymethyl)thymidine (DHMT) and base-phosphorus-carbon-base (BpcB)]

**Fig. 8:**
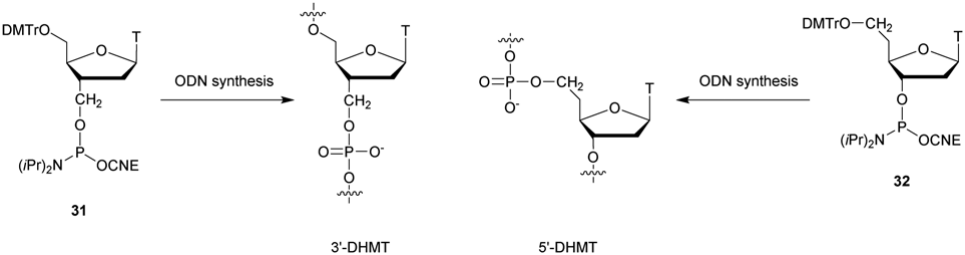
Chemical structures of 3′- and 5′-DHMT linkages obtained from 3′-DHMT **31** and 5′-DHMT **32** phosphoramidite building blocks.^193^

Several groups have reported the replacement of the phosphodiester linkage with a methylene phosphonate.^192–196^ However, only a few publications concerned resistance tests against nucleases. In 1997, the group of Pedersen described the synthesis of modified thymidine having an additional bridging methylene at the 3′ or 5′ extremities, which implies an elongation of the internucleoside linkage (Fig. 8).^193^

The synthesis of the phosphoramidite **31** was performed in 10 steps with 10% overall yield from thymidine using a 3′-cyano nucleoside^197^ reduced to a hydroxymethyl substituent as a key step. Considering a bridging methylene at the 5′ end of the building block **32**, 7 steps are required from thymidine. The introduction of the methylene was done by oxidation of the 5′ alcohol under Swern conditions^198^ followed by the homologation of the aldehyde under standard Wittig reaction conditions with methyltriphenylphosphonium bromide.^199^ An hydroboration/oxidation sequence led to the desired intermediate. These two building blocks (**31** and **32**, Fig. 8) were then used to synthesize different modified ODN using the phosphoramidite methodology. Thermal denaturation studies showed that the presence of 3′-DHMT or 5′-DHMT induces significant decreases in stability of the duplexes formed with their complementary DNA strand. Thereafter, the authors decided to study the resistance of these ODN against Exo III nuclease. The experiments performed demonstrated that the 3′-DHMT linkage allowed a complete inhibition of Exo III. However, the 5′-DHMT linkage achieves no resistance to the nuclease activity.

**Fig. 9:**
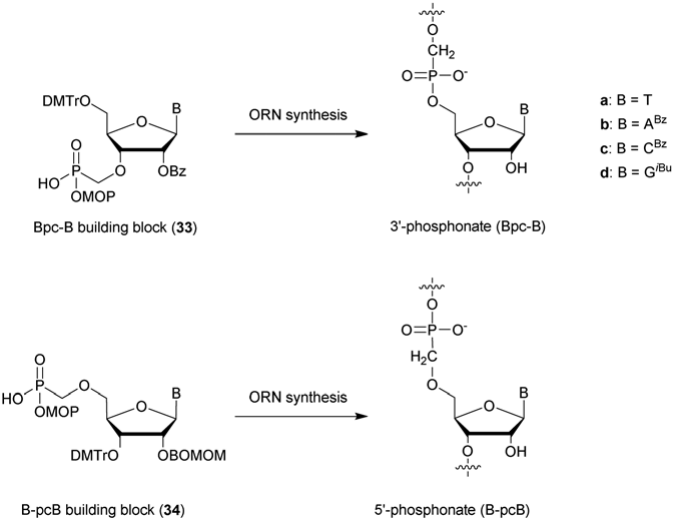
Chemical structures of regioisomeric 3′-phosphonate (Bpc-B) and 5′-phosphonate (B-pcB) linkages obtained from Bpc-B **33a–d** and B-pcB **34a–d** building blocks.

In 2011, Pav *et al.* reported results working on RNA analogues by inserting the carbon atom into the P–O instead of the C–O bond, leading to 3′-phosphonate (Bpc-B) and 5′-phosphonate (B-pcB) linkages (Fig. 9).^196^

They described the synthesis of phosphonate synthons **33** and **34** in order to incorporate the modified nucleosides using the phosphotriester methodology (Fig. 9). The key step was the nucleoside functionalization with diisopropyl tosyloxymethylphosphonate in the presence of sodium hydride. Various ORN were synthesised containing either Bpc-B or B-pcB linkages. In all cases, the presence of these modifications destabilizes the duplexes formed with a complementary ORN. The decrease of the *T*_m_ values is more pronounced with the Bpc-B to the point that a 9-mer ORN possessing only Bpc-B linkages cannot hybridize with its complementary strand under physiological conditions. The authors then studied the resistance of their modified ORN to RNase-A and PDE I and II. RNase-A is unable to cleave the modified Bpc-B or B-pcB linkages, although hydrolysis of the ORN occurs with normal kinetics until a modified linkage is reached. Regarding PDE, it was observed that they were able to ignore a modified link and “jump” to the next internucleoside linkage in order to cleave it. At the end of the 2 h experiment, dimers possessing a modified linkage are observed. It would have been interesting to examine if two or three successive modified internucleoside linkages could lead to the inhibition of the PDE but these experiments were not conducted. The possibility for Bpc-B or B-pcB modified ODN to activate RNase-H for antisense applications was evaluated by Rejman *et al.*^200^ They demonstrated that these modifications achieved total resistance to nucleases of L1210 cell free extracts and prevented the activation of RNase-H. A study concerning the use of methylene functionalization for ODN capping may be of interest to inhibit exonucleases. However, the strong destabilization of the duplexes induced by this modification limits its potential for biological applications.

##### 3.1.4.5 5′-Alkylphosphonate linkages: ethyl (EtP), vinyl (VP) and ethynyl phosphonate (EP) functionalization

In 1993, Caruthers and co-workers described the synthesis of ethylphosphonate (EtP) linked thymidine dinucleotides along with their incorporation within ODN *via* a phosphoramidite derivative.^201^ The authors did not perform annealing or nuclease resistance experiments but this work paved the way for 5′-alkylphosphonate modification of the internucleoside linkage.

**Fig. 10:**
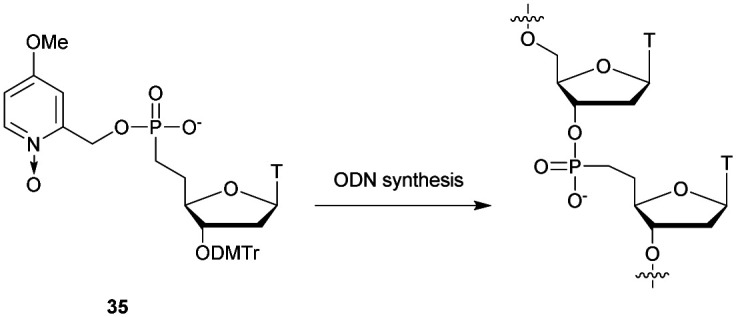
Chemical structures of the 5′-ethylphosphonate linkage obtained from *C*-phosphonate building block **35**.

Years later, the group of Stawinski synthetized several homothymidylates from 5 to 20-mers bearing only the EtP linkage.^202^ They used a different approach. Indeed they exploited the 4-methoxy-1-oxido-2-picolyl as a phosphonate protecting group, able to enhance the rate of the esterification of the *C*-phosphonate function (Fig. 10).

Indeed, a neighbouring group participation was observed during the coupling with 2,4,6-triisopropylbenzenesulfonyl chloride (TPS-Cl) as a condensing reagent. ODN elongation *via* solid supported synthesis was manually performed by successive coupling of **35** (5′ → 3′ elongation) using TPS-Cl as a condensing agent and deprotection of the DMTr protecting group. Finally, cleavage of the phosphonate protecting group was done using thiophenol-triethylamine and the ODN was released from the solid support by aqueous ammonia treatment. Nuclease stability experiments were performed against SVPDE (which cleaves P-*O*-C3′ linkages) and BSPDE (which cleaves P-*O*-C5′ linkages). As expected, the EtP linkages were totally stable against BSPDE but showed no resistance increase to SVPDE.

Duplex stability experiments were performed later by Hutter *et al.* that described the synthesis of ODN containing EtP linkages through the use of phosphoramidite dimers.^203^ The *T*_m_ values were determined with the complementary DNA strand of fifteen ODN of different lengths comprising one or two modifications at different positions. The general result obtained was a destabilization of about −3 °C per modification. This significant decrease in stability along with the sensitivity of the EtP internucleoside linkage to SVPDE prevented further biological application of this modification.

**Scheme 15:**
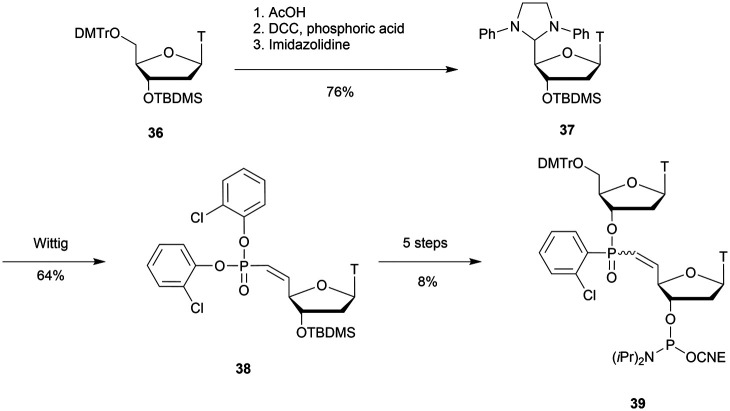
Synthesis of VP-dithymidine phosphoramidite **39**.

Homothymidylate ODN having 5′-vinylphosphonate (VP) internucleoside linkages were synthesised in 1996 by the group of Caruthers.^204^ The synthetic strategy envisaged was to develop a 3′-thymidine phosphoramidate dimer, **39**, protected with a 5′-*O*-DMTr group in order to use it in supported synthesis. The latter was synthesised from 5′-*O*-DMTr-3′-*O*-TBDMS-thymidine (**36**) which was functionalized with a 5′ masked aldehyde in 3 steps: deprotection of DMTr, oxidation of alcohol using Pfitzner–Moffatt oxidation conditions^205^ and finally *in situ* protection of the aldehyde as an imidazolidine, **37**. In addition, a Wittig ylide reagent was prepared and then treated with the freshly released aldehyde, leading to the thymidine precursor having a 5′ vinylphosphonate linkage, **38**. Conventional coupling/protection/functionalization reactions lead to the phosphoramidite dimer **39** (Scheme 15).

Several 14-mer homothymidylates were then synthesised, comprising 0, 1 or 6 VP internucleoside linkages. Thermal stability experiments (*T*_m_ evaluations) with complementary ODN were conducted along with resistance evaluation against SVPDE. The presence of VP linkages destabilized the duplexes formed with the complementary (d(A_14_)) by −3 °C per modification (comparable to the EtP linkage^203^). Nuclease digestion experiments showed that the VP linkage, as the EtP one,^202^ is totally resistant to SVPDE over 20 min. The ODN comprising a single modified linkage only exhibits slightly increased stability. These first studies demonstrated that the VP linkage offers excellent nuclease resistance. However, the strong destabilization of the duplexes formed limits its potential for antisense applications. Activation tests for RNase-H have not been reported so far.

**Fig. 11:**
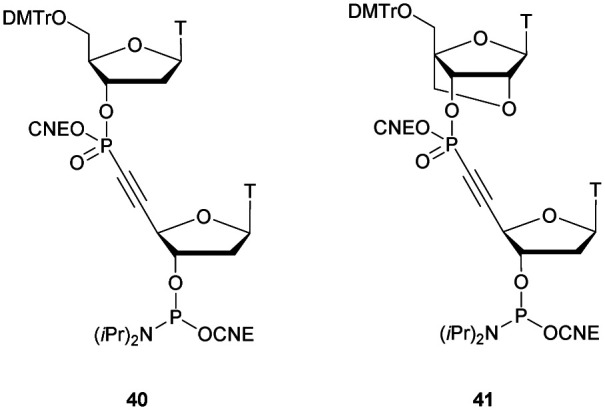
Chemical structures of EP-linked phosphoramidite thymidine dimers **40** and **41**.

Recently, the group of Obika published the synthesis of thymidine dimers having an ethynylphosphonate (EP) linkage.^206^ The goal of this work was to incorporate a 5′ alkylphosphonate linkage within ODN able to hybridize stably with their complementary strand and study their biological properties. Indeed previous studies concerning 5′ alkylphosphonate modification have shown its weak duplex forming ability. The repulsion between the nucleobase and the hydrogen atom(s) at the C6′-position induces deleterious effects on the duplex formation as demonstrated on EtP and VP internucleoside linkages.^203,204^ The authors synthetized EP linked thymidine dinucleotides having either two natural riboses or one LNA modified nucleoside (Fig. 11).

**Scheme 16:**
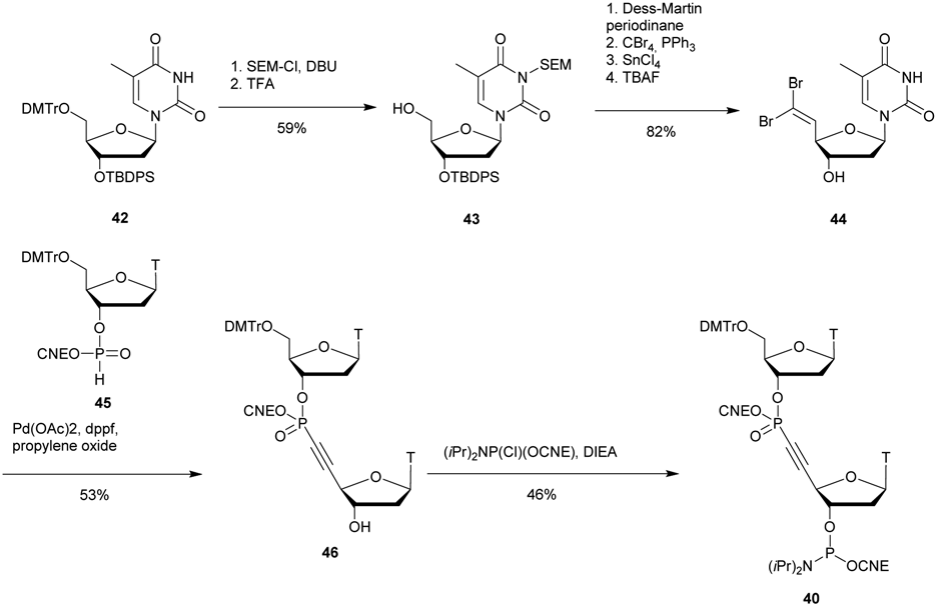
Synthesis of the phosphoramidite thymidine dimer **40**.

The synthesis of dimer **40** started with the protection of the base of **42** with a 2-(trimethylsilyl)ethoxymethyl (SEM) protecting group, followed by the removal of the DMTr one by acidic treatment, leading to derivative **43**. The free 5′ hydroxyl was oxidized using Dess–Martin periodinane and the resulting aldehyde was converted into 1,1-dibromoalkene by the Corey–Fuchs reaction. The SEM and TBDPS groups were cleaved with tin(iv) tetrachloride and TBAF, respectively, to give nucleoside **44**. The latter was coupled to the *H*-phosphonate derived from 5′-*O*-DMTr-thymidine **45** using Pd(OAc)_2_ under microwave irradiation. Dimer **46** obtained was finally subjected to a classical phosphitylation, leading to the desired building block **40** (Scheme 16). Dimer **41** was prepared using a similar strategy.

The building blocks **40** and **41** were incorporated within ODN using classical phosphoramidite chemistry with prolonged coupling time. However, despite several attempts to optimize them, low yields were obtained during ODN synthesis (0.2–2.2%). Melting experiments were then performed with different ODN comprising the EP internucleoside linkage with the complementary DNA or RNA strand. The main results are that a single EP linkage implied a strong destabilization with the complementary DNA (Δ*T*_m_ ∼ −5 °C per modification) or RNA (Δ*T*_m_ ∼ −4 °C per modification) strand, which are of the same order as EtP or VP internucleoside linkages.^208,209^ Thus, the authors decided to study the effect of the dimer whose one of the nucleosides was replaced with a LNA, known to allow better stability of the duplexes. As expected, the presence of the LNA bearing the EP linkage at its 3′ position showed a lower destabilization with the complementary DNA strand and no destabilization with the RNA one. Thereafter, the resistances of several ODN bearing the EP internucleoside linkage at either their 3′ or 5′ extremities were evaluated against the SVPDE or BSPDE (Table 17).

**Table 17:** Resistance evaluations of PS- and EP–ODN against SVPDE and BSPDE^206^

ODN (5′ → 3′)^a^	Remaining intact ODN after 80 min of incubation^b^ (%)	
	SVPDE	BSPDE
d(TTTTTTTTTT)	0	—
d(TTTTTTTTT_PS_T)	83	—
d(TTTTTTTTT_EP_T)	7	—
d(TTTACGCAGTTT)	—	0
d(T_PS_TTACGCAGTTT)	—	79
d(T_EP_TTACGCAGTTT)	—	88

a EP refers to the ethynylphosphonate linkage.

b ODN not tested.

The percentage of intact ODN was quantified after 80 min of incubation. Concerning the SVPDE, while the natural ODN was completely digested and 83% of its analogue bearing a PS internucleoside linkage at its 3′ extremity was intact, the EP analogue was still detectable (7%), demonstrating a slight resistance increase compared to the natural linkage. To the BSPDE, the EP internucleoside linkage provided a superior resistance to the PS one. Thus, the authors highlighted a difference concerning the resistance achieved by the EP internucleoside linkage towards the SVPDE 3′-exonuclease (moderate) and the BSPDE 5′-exonuclease (significant). Finally, the RNase-H recruiting ability of EP modified ODN was evaluated. Several 14-mer gapmer ODN comprising a single EP linkage at different positions in the gap region were synthetized. The latter were flanked with three 2′-OMe modified nucleosides. The natural and PS-modified ODN induced the recruitment of RNase-H as expected. Interestingly, the ODN bearing an EP linkage drastically changed the cleavage site of the enzyme (demonstrated by gel electrophoresis). Indeed RNase-H is able to recognize several residues on a sequence and the inclusion of one EP internucleoside linkage into the RNase-H recognition region implied a decrease of the enzyme activity at the main cleavage site. Thus, the cleavage site can be controlled by the use of the EP linkage.

The 5′-alkylphosphonate linkage described in this section induced a strong destabilization of the duplexes formed with both the complementary DNA and RNA strands. The use of the LNA modified nucleoside by the group of Obika counterbalanced this negative effect. Significant nuclease resistance was observed in the case of enzymes that hydrolyse P-*O*-C5′ linkages. Finally, an interesting application was described using the EP modification in order to control the cleavage site of the RNase-H which can allow allele selective gene regulation^207^ and enhance therapeutic applications.^208^

##### 3.1.4.6 Phosphonoacetate (AcPO) and thiophosphonoacetate (AcPS) functionalization

**Fig. 12:**
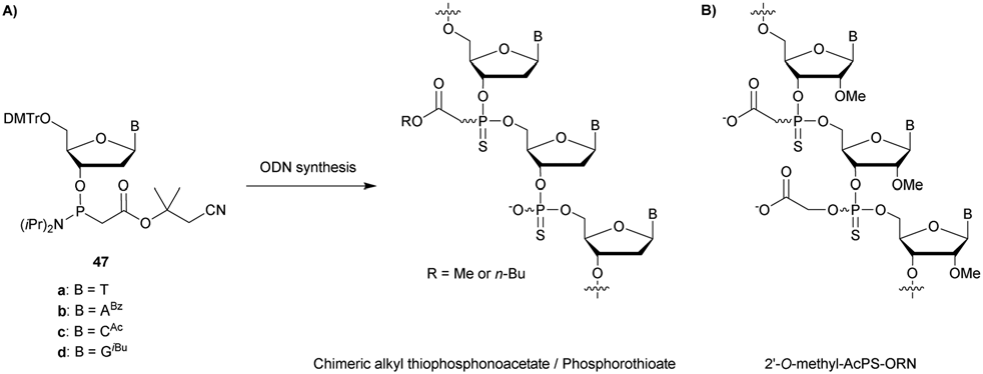
(A) Chemical structures of the chimeric ODN used for *in cellulo* penetration experiments^210^ obtained from the protected phosphoramidite building blocks **47a–d**; and (B) Chemical structures of the 2′-*O*-methyl-AcPS-ORN used for *in cellulo* penetration experiments as inhibitors of HTT expression.^211^

In 2003, the group of Caruthers described the solid phase synthesis of phosphonoacetate (AcPO) and thiophosphonoacetate (AcPS) ODN.^209^ A major preliminary study allowed them to obtain a suitable phosphinylacetic ester derivative *via* the Reformatsky reaction. This synthon allowed them to prepare different phosphoramidite building blocks **47a–d** used thereafter for AcPO- and AcPS-ODN synthesis (Fig. 12A). The ODN elongation required adjustments compared to the conventional phosphoramidite chemistry.

Indeed the electron-withdrawing acetyl ester deactivates the phosphorus atom during tetrazole activation. Thus, longer coupling times were necessary in order to obtain high yields. Fully modified 18-mer phosphonoacetate and thiophosphonoacetate were successfully synthesised. Resistance to SVPDE degradation was then evaluated (Table 18). Both analogues displayed total resistance during the course of the experiment contrary to their PO and PS counterparts. Finally, the ability of these ODN to activate RNase-H was investigated to determine their potential for antisense applications. The authors used *E. coli* RNase-H and monitored its activity by gel electrophoresis. All negative controls were performed and were consistent, including the inactivation of RNase-H with a 2′-*O*-Me-ORN. PO- and PS-ODN induced the enzyme activity as expected. The AcPO and AcPS analogues were also able to activate RNase-H. However, a significant difference in efficiency was observed between the two analogues. The reduced activity with the sulfurized AcPS might be explained by steric and hydrophobic effects within the enzyme active site. A year later, the same laboratory group completed their first study with biochemical characterization.^210^ They highlighted the significant destabilization caused by the AcPO or AcPS linkages on duplexes formed with their complementary ODN strand (Δ*T*_m_ ∼ −1.5 °C per modification). Resistance to other nucleases was also evaluated (DNase I and HeLa cell nuclear extracts) with total stability of AcPO- and AcPS-ODN. Afterwards, the authors took a close look at their antisense potential by quantifying the stimulation of *E. coli* RNase-H activity according to the chemical structure of the ODN (Table 18).

**Table 18:** Half-life evaluations of AcPO- and AcPS-ODN against SVPDE^209^ and initial rates for RNase-H^210^

ODN (5′ → 3′)^a^	*t*_1/2_ b	Initial rate (min^−1^)
d(CTCAAGTGGGCTGGTGAC)-all-PO	<15 min	0.577
d(CTCAAGTGGGCTGGTGAC)-all-PS	>18 h	0.244
d(CTCAAGTGGGCTGGTGAC)-all-AcPO	>18 h	0.000522
d(CTCAAGTGGGCTGGTGAC)-all-AcPS	>18 h	0.0134
d(C_AcPS_T_AcPO_C_AcPO_A_AcPO_AGTGG	—	1.421
GCTGG_AcPO_T_AcPO_G_AcPO_A_AcPO_C)		
d(C_AcPS_T_AcPS_C_AcPS_A_AcPS_AGTGG	—	1.266
GCTGG_AcPS_T_AcPS_G_AcPS_A_AcPS_C)		

a PO, PS, AcPO and AcPS refer to the phosphodiester, phosphorothioate, phosphonoacetate and thiophosphonoacetate internucleoside linkages respectively.

b ODN not tested.

Surprisingly, both chimeric AcPO/PO and AcPS/PO significantly accelerated the kinetics of RNase-H compared to the natural PO-ODN or PS-ODN. The authors assumed that the reduced *T*_m_ for the AcPS–RNA duplex accelerated product release and consequentially enhanced the turnover of the RNase-H. These encouraging results prompted the authors to study the cellular penetration of esterified (with either methyl or *n*-butyl groups) chimeric AcPS/PS-ODN (Fig. 12A). The strategy was to increase the cellular penetration of the ODN by masking a part of the negative charges owned by the AcPO and AcPS linkages in the form of esters. The carboxylates were regenerated *in cellulo* by the esterases present. The first results obtained were encouraging with a significant amount of ODN accumulated in the nucleus after passive cell penetration. Based on these results, AcPO and AcPS modified ODN may be useful for controlling gene expression *via* an antisense mechanism. This was partially demonstrated in a recent publication concerning the inhibition of human huntingtin (HTT) protein expression in cells.^211^ Huntington's disease is a currently incurable genetic disease caused by an expansion of the trinucleotide CAG within the HTT gene. The authors synthesised AcPS modified 2′-*O*-methyl-ORN as inhibitors of human huntingtin (HTT) expression (Fig. 12B). The thermal stability of these ORN was studied with their complementary RNA strand along with *in cellulo* experiments in order to decrease the biosynthesis of HTT protein. AcPS modified 2′-*O*-methyl-ORN exhibited a significant stabilization of the duplex formed with its complementary RNA strand thanks to the presence of the 2′-*O*-methyl substituent which counterbalanced the deleterious effect of the AcPS linkage. *In cellulo* experiments demonstrated that the 2′-*O*-methyl-AcPS-ORN was able to inhibit protein expression by steric blocking up to 60%, opening the way to further investigations concerning the AcPS linkage in the field of AS therapies.

##### 3.1.4.7 Phosphonoformate (FP) functionalization

**Scheme 17:**
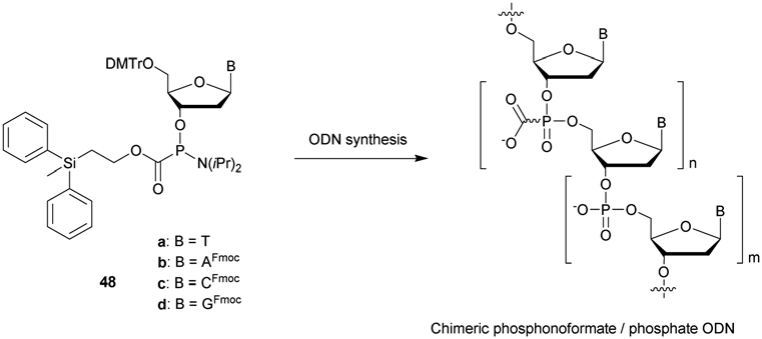
Chemical structures of phosphonoformate linkages obtained from phosphoramidite building blocks **48a–d**.

Being active in the development of P–C internucleoside linkages, Caruthers and co-workers also reported a solid phase synthesis strategy for the preparation of phosphonoformate (FP) ODN.^212^ The appropriate building blocks were obtained with a similar strategy to the one developed for AcPO presented above. Diphenylmethylsilylethyl chloroformate reacted with bis(*N*,*N*-diisopropylamino)phosphine in the presence of sodium metal to yield [bis(diisopropylamino)phosphino]-β-(diphenylmethylsilyl)ethyl ester. This reagent was then condensed with suitably protected 2′-deoxynucleosides in the presence of 4,5-dicyanoimidazole to give the desired phosphoramidite monomers **48a–d** (Scheme 17).

Several ODN have been synthesised using Q-linkers,^213^ some all-FP-ODN but also chimeric FP/PO-ODN. The homothymidylate bearing FP linkages surprisingly formed a more stable duplex with its complementary ORN strand with a *T*_m_ value increased by 11 °C compared to the unmodified one (Table 19). This thermal stabilization decreased with the number of FP modifications. However, no stabilization was observed when the FP modifications were non-adjacent. This result seems to indicate a stabilizing effect in solution due to multivalent ion chelations by adjacent acetate function. The resistance of two homothymidylate 14-mers either fully modified or bearing a FP linkage every third position was evaluated in the presence of DNase I, SVPDE and HeLa cell extracts. Both FP-ODN were completely resistant to enzymatic hydrolysis during the course of the experiments (Table 19). The activation of RNase-H induced by these FP- and FP/PO-ODN was then tested (as well as FP/PO-ODN having FP linkages at the extremities, Table 19). The results showed that all these ODN activated RNase-H. In particular, sequences having FP linkages in a regular manner slowed down the activity of RNase-H, while the ODN flanked with FP linkages accelerated RNase-H activity (similar to AcPO).^210^ These results combining the formation of stable duplexes and activation of RNase-H make the FP modification very interesting. However, these modified ODN have never been studied in the context of biological application despite their strong potential.

**Table 19:** Thermal denaturation studies (*T*_m_ values) of FP-ODN with complementary DNA and their enzymatic stabilities towards digestion with SVPDE^212^

ODN (5′ → 3′)^a^	*T*_m_ with DNA (°C)	*t*_1/2_ b			RNase-H activation
		DNase I (min)	SVPDE (min)	HeLa cell extracts (min)	
d(T_14_)	36	<2	<2	<2	✓
d(T_FP_T)_7_	47	>180	>180	>180	✓ (slow)
d(T_FP_T_FP_T_FP_T_8_T_FP_T_FP_T)	42	—	—	—	✓ (fast)
d(T(TT_FP_T)_4_T)	35	>180	>180	>180	✓ (slow)

a FP refers to the phosphonoformate linkage.

b ODN not tested.

##### 3.1.4.8 1,2,3-Triazolylphosphonate (TP) functionalization

**Scheme 18:**
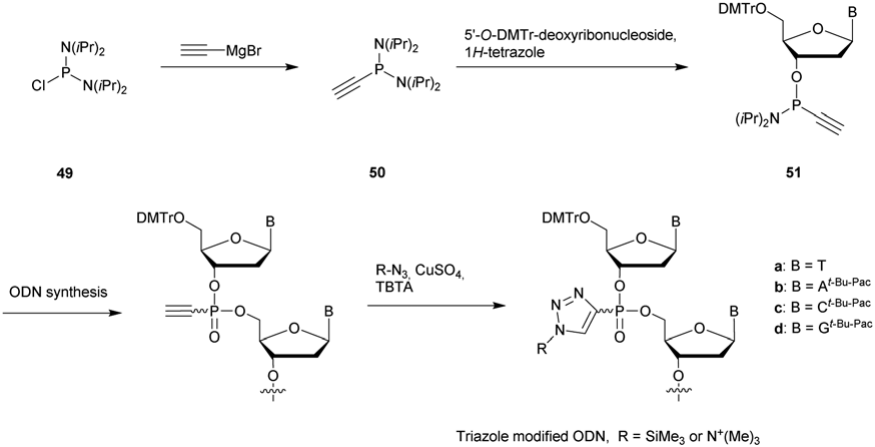
Synthesis of the alkyne phosphoramidite building blocks **51a–d** used for triazolylphosphonate ODN synthesis.^214^

In 2012 Caruthers and co-workers were interested in another modification of the PO linkage by developing a “clickable” alkyne backbone.^214^ The objective was to exploit the copper(i)-catalyzed alkyne–azide cycloaddition (CuAAC) developed in 2002 by Sharpless and Meldal^215,216^ to modify the ODN at the level of the PO linkage to 1,2,3-triazolylphosphonate (TP). The phosphitylating reagent bis(*N*,*N*-diisopropylamino)ethynyl phosphine (**50**) was synthesised *via* the reaction of a Grignard reagent (ethynylmagnesium bromide) on bis(*N*,*N*-diisopropylamino)chlorophosphine, **49**. The latter reacted then with the appropriate 5′-*O*-DMTr-deoxyribonucleoside, leading to the phosphoramidite building blocks **51a–d**. CuAAC reaction was performed after elongation by suspending the CPG in a H_2_O/MeOH/THF solution containing the azide, CuSO_4_ and tris[(1-benzyl-1*H*-1,2,3-triazol-4-yl) methyl]-amine (TBTA) in order to introduce trimethylsilyl or trimethylammonium groups (Scheme 18).

A wide variety of chimeric ODN have been synthesised using these synthons. The ODN consisted of 1,2,3-triazolylphosphonate as well as phosphate or thiophosphate internucleoside linkages. In addition, 2′-*O*Me and LNA modified ribonucleosides were used. Noteworthily, despite the neutral charge of the TP linkage the aqueous solubility of the ODN synthetized was maintained. Enzymatic studies concerned two 23-mer homothymidylates having one or two triazolylphosphonate linkages at both extremities (Table 20).

**Table 20:** Half-life evaluations of TP-ODN against SVPDE and CSPDE^214^

ODN (5′ → 3′)^a^	*t*_1/2_	
	SVPDE	CSPDE
d(T_23_)	<5 min	<10 min
d(T_TP_T_TP_T_18_T_TP_T_TP_T)	3.5 h	>12 h
d(T_TP_T_20_T_TP_T)	2.5 h	>12 h

a TP refers to the triazolylphosphonate linkage.

The study of both 3′- and 5′-exonucleases allowed a reliable evaluation of the TP resistance to exonucleases in order to possibly use it for the synthesis of AS chimeric gapmers. The results clearly demonstrated the resistance provided by this internucleoside linkage. It should be noted that this modification does not allow the nucleases to “jump” above the modification and hydrolyse the rest of the ODN since a single modification prevents significantly the hydrolysis (contrary to methyl or methylene modified internucleoside linkages). Thermal denaturation studies were performed in order to quantify the contributions of TP linkages toward duplex formation. A series of TP linked ODN were hybridized with their complementary miR-15b RNA comprising the sequence 5′-UAGCAGCACAUCAUGGUUUACA-3′. The conclusion of these studies was that the TP linkage induced a slight destabilization of the duplexes of about −0.7 °C per modification. Finally, the cellular penetration of the TP-ODN was evaluated with different 16-mers having various modified linkages and labelled with fluorescein. The results were generally less efficient than with the unmodified ODN. Only the ODN with TP linkages at each extremity showed a better internalization of about 10% in HeLa cells, paving the way for potential therapeutic applications of these chimeric gapmer ODN.

#### 3.1.5 Phosphotriester (PT) linkage

**Scheme 19:**
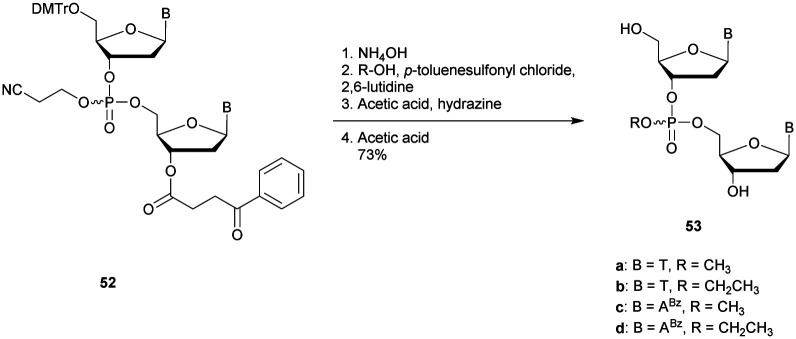
Synthesis of PT-dinucleotides **53a–d**.

The first phosphotriester (PT) linkage derivatives were described by T'so and Miller in the early 1970s.^217–222^ The neutrality of the generated backbone was immediately considered as an undeniable advantage for biological applications in order to enhance cellular uptake. Moreover, these derivatives were able to hybridize with complementary natural PO-ODN, leading to more stable duplexes than unmodified ones due to the removal of electrostatic repulsion. The first synthesis of PT dinucleotides was based on the final alkylation of a phosphate group. Fully CNE protected PT-dinucleotides were prepared according to protocols previously described.^223^ The CNE protecting group was removed from **52** by treatment with aqueous ammonia in pyridine and the resulting phosphate was functionalized with MeOH or EtOH in the presence of *p*-toluenesulfonyl chloride. The final deprotection led to the PT-dinucleotides **53a–d** (Scheme 19).

The resistance of d(T_PT_T) and d(A_PT_A) to SVPDE and micrococcal nuclease was evaluated and the PT linkage was found to be totally resistant to hydrolysis.

Numerous synthesis methods of PT-ODN have been described over the years but did not exceed the stage of the synthesis and will thus not be discussed here.^224–228^ This is probably due to the observation made by T'so and Miller concerning the fact that the neutral PT linkage was totally resistant to nucleases.

**Fig. 13:**
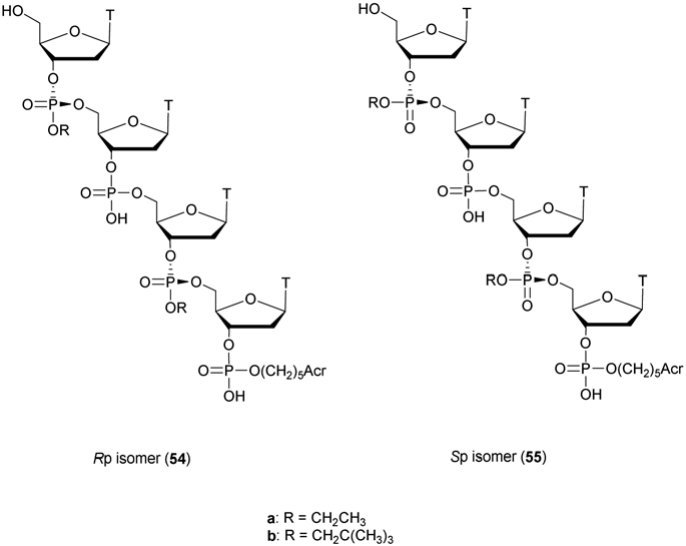
Chemical structures of the *R*_p_**54** and *S*_p_**55** PT stereoisomers.

In 1986 Asseline *et al.* described the synthesis of several 4-mer oligothymidylates involving alternating alkylphosphotriester–phosphodiester backbones.^229^ The key step in the synthesis was the separation of the generated PT-dinucleotide stereoisomers, leading *in fine* to the two *R*_p_**54** and *S*_p_**55** 4-mers. All ODN were functionalized with an acridine at the 3′-end as a fluorescent reporting group (Fig. 13).

Several nucleases were evaluated on the different ODN and compared to their natural counterparts. Both exonucleases CSPDE and SVPDE were inactive against PT-ODN because of the absence of free hydroxyl at the 3′-end and the presence of a PT linkage at the 5′-end. Endonucleases S1 and P1 were able to hydrolyse the natural PO linkages, but not the PT ones. The rates of hydrolyses were found to be lower than those for the natural ODN, demonstrating a protective effect of the adjacent PT linkages. Moreover, this protective effect was more important with the neopentyl than with ethyl group, highlighting a steric hindrance effect.

**Fig. 14:**
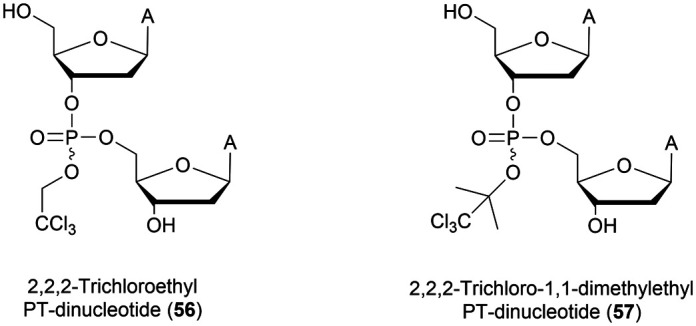
Chemical structures of PT modified dinucleotides **56** and **57** studied by Letsinger *et al.*^155^

Letsinger *et al.* also studied the behaviour of PT linkages.^155^ They synthesised two dinucleotides d(A_PT_A) **56** and **57** bearing chlorinated substituents and evaluated their resistance to SVPDE and CSPDE (Fig. 14). The purpose of these modifications was to introduce bulky lipophilic groups at the phosphorus atom of the nucleotides to bring new properties to the resulting ODN (*e.g.* enhanced cellular membrane interactions, stabilized hybridization…). The nuclease digestion study was performed qualitatively. However, the authors were able to conclude that under standard conditions, in the presence of SVPDE or CSPDE, both phosphotriester dinucleotides were completely stable during the experiment, confirming the results of other research groups. Indeed, *O*-ethyl^230^ and *O*-isopropyl^231^ phosphotriesters have been shown to be totally resistant to SVPDE, CSPDE and Eco-Ri 1 nucleases.

In 2015, Caruthers and co-workers developed a general method to obtain oligonucleotides modified with hydrophobic and/or cationic *O*-alkylated PT internucleoside linkages.^232^ The methodology used bis-(diisopropylamino)-3′-phosphorodiamidite derivatives that can be coupled with an alcohol in the presence of 5-ethylthio-1*H*-tetrazole, leading to *N*-protected amino alcohols instead of the classical cyanoethyl protective group. The different *O*-alkylated phosphoramidites were then incorporated within ODN sequences by solid phase synthesis using 5-ethylthio-1*H*-tetrazole as an activator. The effect of the P-substitution with various amino alcohols on the thermal stabilities of DNA duplexes was evaluated using various 22-mer ODN. Each ODN carried two, four, or six amino alcohol triester linkages (*Z*-l-alaninol, phenylalaninol, *Z*-l-glycinol, and β-alaninol). These modifications were located at the same relative position to allow reliable comparisons. The stability of the duplexes formed between these PT-ODN and their complementary strand was slightly affected by the presence of modified internucleoside linkages. Thereafter, homothymidylate 10-mers having either one *Z*-l-phenylalaninol (PT(PhA)) or one *Z*-l-alaninol (PT(A)) at either the 3′ or 5′ extremity were prepared and tested for resistance to SVPDE and CSPDE (Table 21).

**Table 21:** Half-life evaluations of PT-ODN against SVPDE and CSPDE^232^

ODN (5′ → 3′)^a^	*t*_1/2_ b	
	SVPDE (min)	CSPDE
d(T_14_)	<2	<45 min
d(TTTTTTTTT_PT(A)_T)	—	>24 h
d(TTTTTTTTT_PT(PhA)_T)	—	>24 h
d(T_PT(A)_TTTTTTTTT)	<30	—
d(T_PT(PhA)_TTTTTTTTT)	<30	—

a PT(A) and PT(PhA) refer to *Z*-l-alaninol and l-phenylalaninol phosphotriester internucleoside linkages respectively.

b ODN not tested.

ODN modified with a hydrophobic or cationic phosphotriester linkage at the 5′ extremity were totally resistant to CSPDE even after 24 h of incubation. Their 3′ modified counterparts showed increased resistance to SVPDE with half-lives of about 30 min. Note that these evaluations were performed with a single modification. The global resistance that could be provided by multiple successive PT internucleoside linkages has not been studied. Additionally, cell penetrations of mixed PT modified ODN labelled with fluorescein were studied in the absence of lipid transfection reagents. While the natural ODN did not penetrate the cells, the four PT modified ODN were successfully internalized inside the cells, with percentages varying from 19 to 95% depending on the nature of the PT linkage, its positioning and the concentrations tested. Experiments were performed on adherent (HeLa) and suspension (Jurkat) cells. Results demonstrate that these *N*-protected amino alcohol PT-ODN have the potential to become valuable tools for biological studies.

**Scheme 20:**
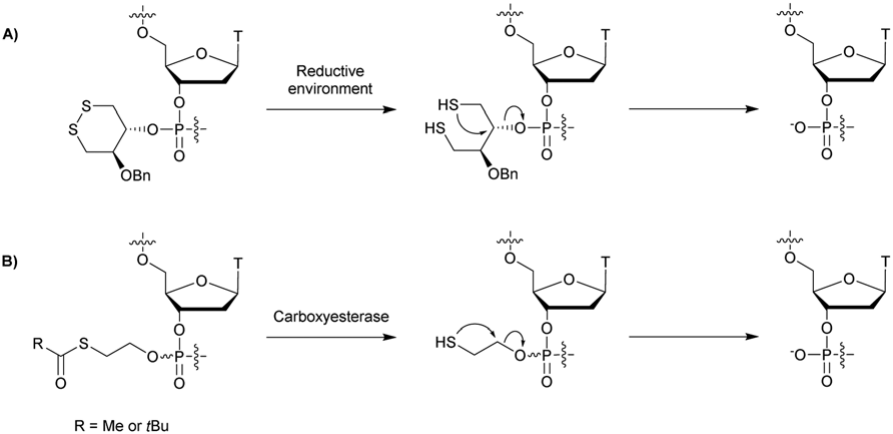
Conversion of PSS-ODN or SATE-ODN into natural PO-ODN in (A) a reducing environment and (B) in the presence of carboxyesterase respectively.

In 2017, Hayashi *et al.* published the synthesis of prodrug-type PT-ODN sensitive to intracellular reduction (Scheme 20A).^233^ They prepared 5′-*O*-dimethoxytrityl-3′-*O*-(±)-*trans*-5-benzyloxy-1,2-dithiane-4-yl *N*,*N*-diisopropylphosphoramidite isomers from a classical phosphoramidite building block and a couple of chiral dithianes. The latter were synthesised from dithiothreitol (DTT) in two steps. ^31^P NMR experiments confirmed that the structure was stable enough to realise solid phase ODN synthesis. *Trans*-5-benzyl-1,2-dithiane-4-yl (PSS) modified thymidines were used to synthesize different PSS-ODN. The nuclease resistance induced by the PSS linkage was evaluated by incubation in FBS (containing mainly 3′-exonucleases). For this purpose a d(T_10_) homothymidylate, as well as its analogue having a single modified PSS linkage at its 3′ extremity (d(T_8_T_PSS_T)), was synthesised. The results showed that the half-life of the modified ODN was multiplied by a factor greater than 15, demonstrating the utility of this modification to increase the resistance of ODN to the 3′-exonucleases. Then, an experiment to mimic the desired reduction of the disulphide *in cellulo* was performed. Thus, the same ODN was incubated at 37 °C in phosphate buffer in the presence of glutathione. The results showed that the modified homothymidylate was transformed into a natural ODN in 75 h (Scheme 20A). Finally, cellular uptake experiments were carried out and showed that with two uncharged PSS-modified linkages the efficiency of the ODN penetration within the cell increased significantly. The authors finally evaluated the ability of an 18-mer ODN bearing 4 modified PSS linkages to silence the gene coding for luciferase. An interesting 20% knockdown activity was observed although the PS analogue appeared to be more efficient with 70% knockdown activity. These first results demonstrate the potential of PSS modification for biological applications although optimizations must be made.

Imbach *et al.* have worked extensively on specific PT linkages as oligonucleotide prodrugs, the S-Acyl-ThioEthyl (SATE) phosphotriesters which are released after the enzymatic hydrolysis of carboxyesterases (Scheme 20B). The development of the SATE linkage has been described in a review dedicated to the use of oligonucleotides as prodrugs.^234^ In 1998, we described the incorporation of the SATE-PT internucleoside linkage within ODN sequences as prodrugs of ODN-AS.^235^ 5′-*O*-DMTr-3′-(SATE) phosphoramidite-thymidine derivatives were synthetized in two steps from 5′-*O*-DMTr-thymidine and *S*-(2-hydroxyethyl)thioacetate or *S*-(2-hydroxyethyl)thiopivaloate.

The building blocks were used to synthesize four different pro-dodecathymidines having phosphate or thionophosphate triester internucleoside linkages. Prolonged reaction times (180 s) were used to ensure high coupling yields along with a photolabile CPG support to avoid degradation of the SATE groups during aqueous ammonia treatment. Numerous stability experiments were then conducted on the four ODN. As expected, a high sensitivity to basic media (aqueous ammonia and 0.1 M aqueous NaOH) was observed, especially in the case of Me-SATE derivatives. Given the high hydrophobicity of *t*Bu-SATE-ODN (and consequently poor water solubility), only the Me-SATE-ODN were studied further. Their stability against pig liver esterase (PLE), SVPDE and CSPDE were studied along with various biological media (*i.e.* total CEM cell extracts (TCE), human serum and human gastric juice, Table 22). These studies showed that the Me-SATE-ODN were not degraded by SVPDE and CSPDE. Moreover, they were not sensitive to acidic media as demonstrated by the incubation in human gastric juice. Finally, the carboxyesterase stability varied depending on the biological medium used. Moreover, the greater lipophilicity of the Me-SATE-PS-ODN increased their resistance to carboxyesterases.

**Table 22:** Half-life evaluations of different Me-SATE-ODN against PLE, SVPDE, CSPDE, TCE, human serum and human gastric juice^235^

ODN (5′ → 3′)^a^	*t*_1/2_					
	SVPDE^b^ (h)	CSPDE^b^ (h)	PLE^c^	TCE^b^^,^^c^^,^^d^ (h)	Human serum^c^ (h)	Human gastric juice^e^ (d)
d(T)_10_-all-Me-SATE-PO-ODN	>19	>28	4.6 h	0.35 (22)	3	>7
d(T)_10_-all-Me-SATE-PS-ODN	>19	>28	>2 d	9.7 (20)	>24	>7

a Me-SATE-PO-ODN and Me-SATE-PS-ODN refer to Me-SATE phosphate and thionophosphate internucleoside linkages respectively.

b Nuclease activity.

c Carboxyesterase activity.

d Values in brackets correspond to the incubation times necessary for the formation of 50% of fully deprotected ODN (carboxyesterase activity).

e Acidic medium stability.

After this pioneering work, Imbach *et al.* continued their research efforts in the context of the SATE-PT internucleoside linkage such as the synthesis of chimeric phosphodiesters and SATE-PT prooligonucleotides,^236,237^ the development of specific tools for their solid supported synthesis^238–240^ and the study by mass spectrometry of the metabolization of SATE-PT-ODN within cell extracts.^234,241,242^ Recently, Meade *et al.* studied several short interfering ribonucleic neutrals (siRNN) containing neutral SATE groups.^228^ They observed an increase of cell delivery and conversion of the siRNN into native ODN by cytoplasmic esterases. siRNN conjugated to a hepatocyte-specific targeting domain have been shown to be active *in vivo* by inducing RNAi responses in mice.

The PT internucleoside linkage presents several advantages. Indeed it can be incorporated by modified solid supported phosphoramidite chemistry, exhibits high nuclease resistance and exhibits interesting properties in the context of prodrug applications as illustrated in this section with recent publications.

#### 3.1.6 Diphosphate diester (di-PO) linkage

**Scheme 21:**
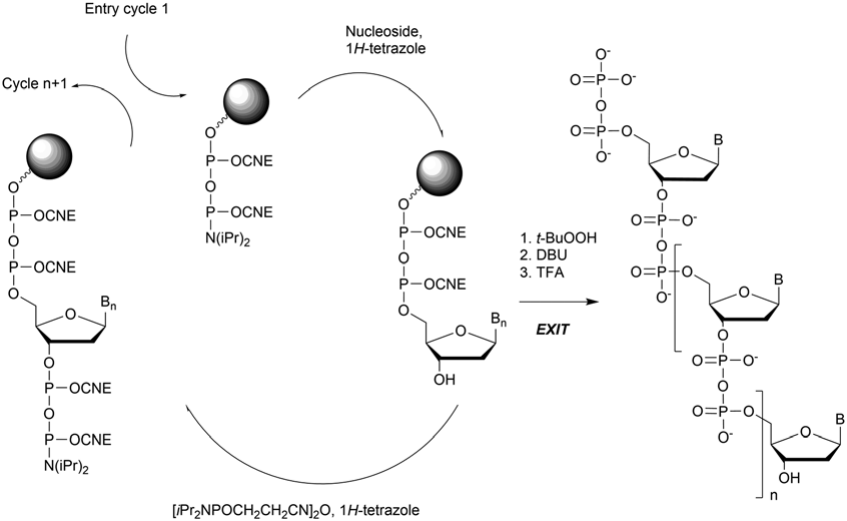
Synthesis cycle of diPO-ODN.

Ahmadibeni *et al.* published in 2007 a study concerning the synthesis of diphosphate diester modified ODN (diPO-ODN).^243^ The implemented phosphoramidite chemistry used unprotected nucleosides and a key diphosphitylation reagent to generate diphosphate internucleoside linkages (Scheme 21). The elongation took place in the 5′ → 3′ direction.

The *T*_m_ values of several diPO-ODN with complementary DNA strands were measured by comparing them with natural ODN with the same sequence. The formed duplexes were always more stable, but in a moderate way (a few degrees of stabilization on a 12-mer). Furthermore, modified ODN exhibited the ability to bind to the complementary unmodified strand (diPO-ODN/ODN duplexes). Thereafter, various modified ODN were incubated with either DNase I or 3′-exonuclease I to determine their nuclease resistance compared with the corresponding unmodified PO-ODN. Under the experimental conditions tested, the natural PO-ODN was hydrolysed significantly after three hours. In parallel, the diPO-ODN were completely stable over 4 h, demonstrating the potential utility of such modifications, which are easily incorporated by synthesis on solid supports. An RNase-H activation study has not been performed so far.

#### 3.1.7 Boranophosphate (BP) linkage

**Scheme 22:**
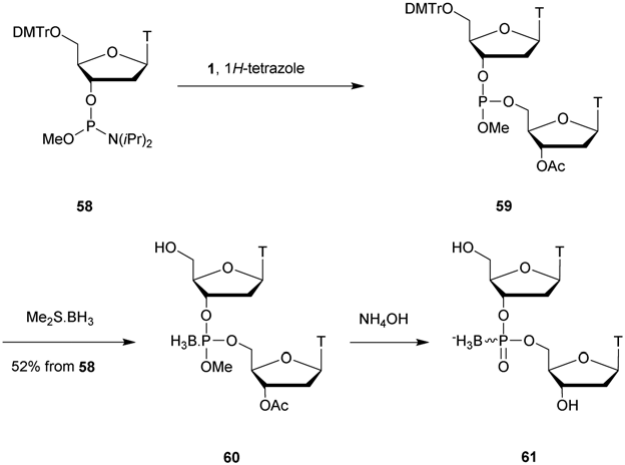
Synthesis of BP-dithymidine, **61**.

The boranophosphate (BP) internucleoside linkage was described for the first time by Shaw and Spielvogel in 1990.^244^ The inherent properties of its analogues and their potential as AS agents triggered a wide variety of research studies.^245–272^ The boranophosphate internucleoside linkage is negatively charged like its natural counterpart. The borane group is isoelectronic with oxygen but more hydrophobic, implying a possible better transmembrane penetration at the cellular level. Boranophosphates are isostructural to methylphosphonates, suggesting an increased resistance to nucleases. The BP linkage is easily obtained by treatment of an intermediate phosphite triester (**59**) with dimethyl sulfide–borane, which removes as well the 5′-DMTr protecting group (Scheme 22).

In this first publication, the authors mentioned that a boranophosphate internucleoside linkage in a dithymidine was particularly stable not only against acidic (1 N aq. HCl/MeOH) or basic (concentrated NH_4_OH at 55 °C) hydrolysis but also against SVPDE or CSPDE digestion. Under the same conditions, while the PO-ODN was hydrolysed to more than 97%, only 8% of the BP-ODN was degraded.

**Scheme 23:**
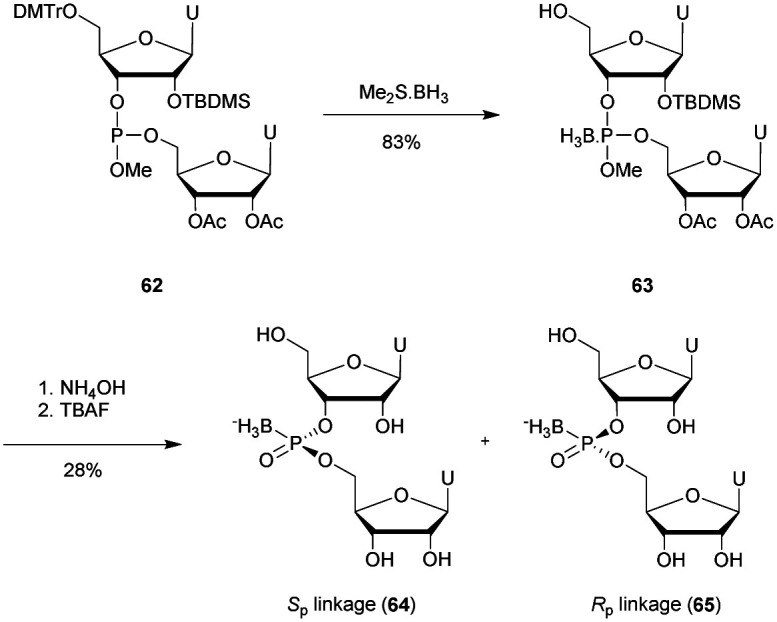
Synthesis of BP-diuridine diastereoisomers **64** and **65**.

Following this early work, Chen *et al.* evaluated the properties of the BP modification as well as the influence of the chiral configuration of the phosphorus atom by connecting two uridines.^273^ A diastereoisomeric mixture of BP-diuridines **64** and **65** was obtained after oxidation of the phosphite triester **62** with dimethyl sulfide–borane and full deprotection using aqueous ammonia and TBAF (Scheme 23).

The diastereoisomers were separated in a 1 : 1 ratio by reversed phase HPLC to give **64** and **65** in 18.9% and 21.3% yield respectively. The absolute configuration of the chiral phosphorus atom was assigned relying on the isoelectronic structure of the BP linkage with the PS one and the specific properties reported concerning the PS diastereoisomers.^146,182,274,275^ The resistance of both BP diastereoisomers **64** and **65** was tested against SVPDE, showing a significant increase in the resistance of the *S*p diastereoisomer with a half-life of about 80 h compared to PO-diuridine (*t*_1/2_ = 7 min). Furthermore the *R*_p_ diastereoisomer was totally stable during the course of the experiment, in agreement with the results observed with PS-ODN by taking into account that the comparable isoelectric configurations of the PS and BP internucleoside linkages have opposite configurations according to the Cahn–Ingold–Prelog attribution rules. These conclusions based on SVPDE hydrolysis kinetics were later supplemented by conformational analyses. The group of Shaw performed circular dichroism and NMR analysis on dithymidines.^276^ The absolute configuration at the chiral phosphorus atom of the dithymidine diastereoisomers was rigorously assigned.

**Scheme 24:**
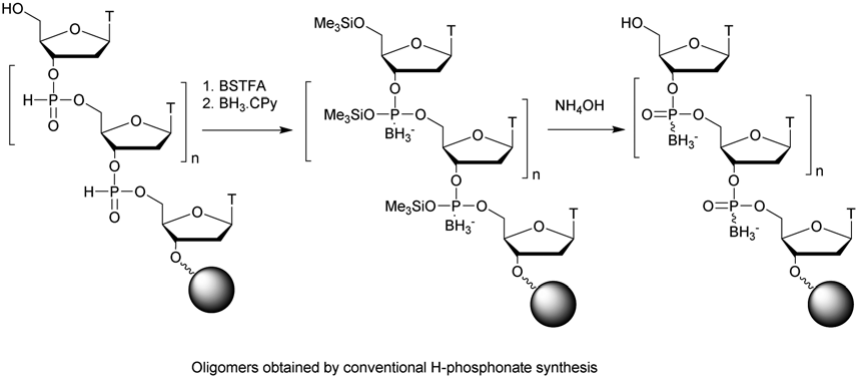
Solid supported synthesis of BP-ODN.

Sergueeva *et al.* confirmed these observations on the diastereoisomers of d(AC) synthesised by the *H*-phosphonate method.^277^ Once again the *R*_p_ isomer was totally stable against SVPDE during the course of the experiment, whereas the *S*_p_ isomer (*t*_1/2_ = 18 h) was more resistant compared to the PO-ODN (*t*_1/2_ < 1 min). Moreover, the authors developed the synthesis of fully modified boranephosphate oligomers using the classical H-phosphonate chemistry followed by an efficient global boronation by a borane–amine complex (Scheme 24).^278^

Oligothymidylates up to 12-mers were synthesised in good yields and their resistance to exo- and endonucleases was evaluated. Two series of experiments were conducted at low and high nuclease concentrations to discriminate the ODN more easily. Results with high concentrations of enzymes are presented in Table 23. The natural ODN is rapidly hydrolysed by all nucleases. The better resistance of PS-ODN to nucleases described in a previous part of this review is observed (see Section 2.1.1). The BP-ODN is more stable than the PS-ODN against SVPDE, P_1_ nuclease and S_1_ nuclease. However, the PS-ODN is the most stable against the BSPDE.

**Table 23:** Half-life evaluations of PS- and BP-ODN against SVPDE, BSPDE, P_1_ nuclease and S_1_ nuclease^278^

ODN (5′ → 3′)^a^	*t*_1/2_			
	SVPDE	BSPDE	P_1_ nuclease	S_1_ nuclease
d(T_12_)	<1 min	<1 min	<1 min	<1 min
[d(T_12_)]-all-PS	2 h	3 h	<5 min	<5 min
[d(T_12_)]-all-BP	7 h	1 h	6 h	70 h

a PS and BP refer to the phosphorothioate and boranophosphonate internucleoside linkages respectively.

**Scheme 25:**
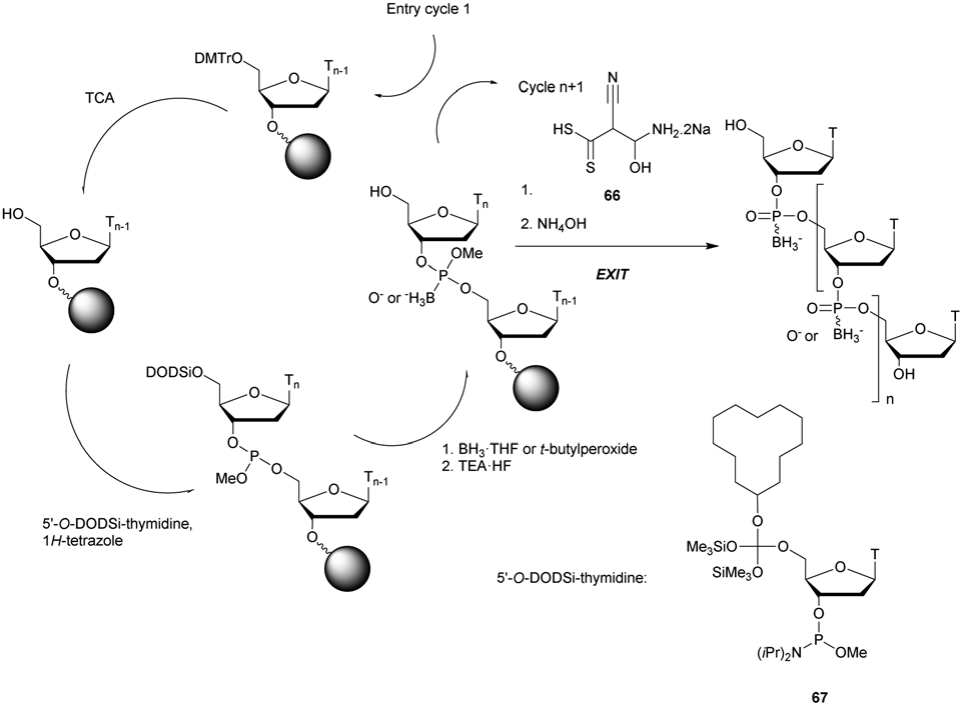
Synthesis cycle of chimeric BP/PO-ODN.

The approach presented above in order to synthetize BP-ODN by a post-synthetic boronation prevents the synthesis of ODN with alternating BP and PO linkages. Thus, the group of Caruthers used bis-(trimethylsiloxy)cyclododecyloxysilyl ether (DODSi) as a 5′ protective group, allowing the synthesis of chimeric BP/PO-ODN.^279^ After the coupling step, the P(iii) oxidation was performed with BH_3_·THF or *t*-butyl peroxide. Treatment with disodium-2-carbamoyl-2-cyanoethylene-1,1-dithiolate (**66**) removed the methyl group and the ODN was released from the solid support with concentrated aqueous ammonia (Scheme 25).

Four ODN were synthesised using this methodology: the natural ODN d(T_14_), its analogue possessing only BP linkages and two others having a BP linkage every two or three nucleosides. The stability experiments of the duplexes formed with their complementary ODN or ORN strand showed a destabilization of about −2 °C per modification, making the duplexes with fully modified 14-mer BP-ODN unstable under physiological conditions (Table 24). The resistance of these ODN was tested against DNase I and SVPDE.

**Table 24:** Thermal denaturation studies (*T*_m_ values) of BP-ODN with complementary DNA and RNA and their half-life evaluations against DNase I and SVPDE^279^

ODN (5′ → 3′)^a^	*T*_m_ with DNA (°C)	*T*_m_ with RNA (°C)	*t*_1/2_ b		RNase-H activation
			DNase I (min)	SVPDE (min)	
d(T_14_)	47	36.8	<2	<2	✓
d(T_BP_T_BP_)_7_	16.8	6.8	∞	∞	✗
d(T_BP_T)_7_	31.1	21.6	∞	∞	✓
d(T_BP_TTT_BP_TTT_BP_TTT_BP_TTT_BP_T)	38	28.5	∞	Slow hydrolysis	✓

a BP refers to the boranophosphonate internucleoside linkage.

b Duration of the experiment not specified.

A total resistance was observed during the course of the experiments with the exception of the analogue having a boranephosphate linkage at every third position. In addition, the ability of the modified ODN to activate RNase-H was assessed (Table 24). Although RNase-H is not activated in the case of a completely modified ODN, the use of chimeric BN/PO-ODN allows the activation of RNase-H while increasing nuclease resistance.

**Fig. 15:**
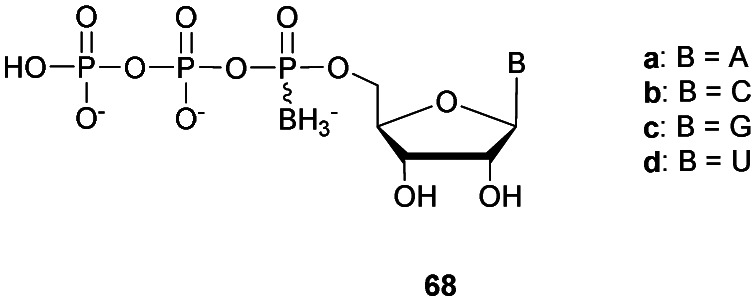
Chemical structures of the 5′-(α-*P*-borano)triphosphates **68a–d** synthesised.

In an effort to study the effect of the boranophosphate stereochemistry on siRNA efficacy and resistance to nucleases, Hall *et al.* managed to incorporate BP linkages within ORN *via* T7 polymerase *in vitro* transcription using 5′-(α-*P*-borano)triphosphates of adenine, cytosine, guanine, and uracil **68a–d** (Fig. 15).^256^

**Scheme 26:**
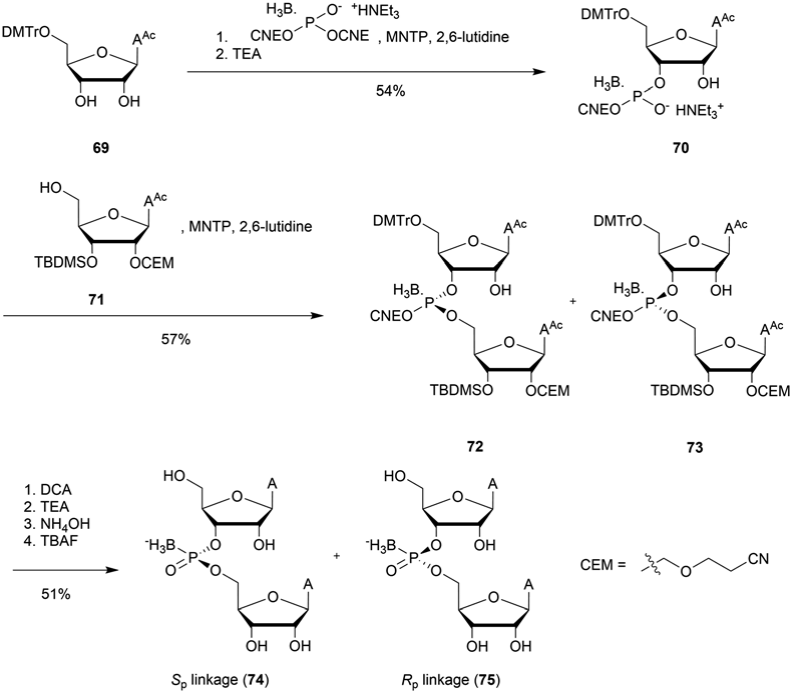
Synthesis of BP-diadenosines **74** and **75**.

Modified ORN with either *R*_p_-PS or *S*p-BP were obtained, and their respective resistances to nucleases and *in cellulo* activities were compared to their PO counterparts.^280^ Many double strands were synthesised by varying the nature, number, and location of the modified internucleoside linkages. Only a few representative duplexes are provided in Table 25. The translation of the enhanced green fluorescent protein (EGFP) in HeLa cells was inhibited by several siRNA. The authors demonstrated that siRNA with multiple BP modified internucleoside linkages at the center of the sense strand show a decrease in activity compared to the non-modified strand (Table 25). This result was in agreement with data from the literature explaining that even minor sequence changes in the middle of siRNAs can drastically reduce its activity.^281^ With the exception of this particular case of multiple modifications at the center of the antisense strand, BP–siRNA duplexes were always more active than the natural one. Moreover, with an equivalent number of modifications it was also shown that the BP-siRNA were more active than the corresponding PS–siRNA duplexes. Regarding nuclease resistance, the BP modified duplexes were tested against a mixture of nucleases from the bovine pancreas (high nuclease concentration because all siRNA tested were stable in FBS for at least 24 h). All the BP modified siRNA showed increased resistance compared to the natural one (up to 10 times). This increase in resistance was confirmed on diastereoisomeric diadenosine boranophosphates **74** and **75** a few years later by Enya *et al.*^282^ These dimers were obtained from protected ribonucleosides using the boranophosphotriester method. The protected adenosine **69** was transformed into boranophosphate monomer **70** in several steps using bis(2-cyanoethyl) boranophosphate. After condensation with a second adenosine derivative the pure *S*_p_**74** and *R*_p_**75** isomers were separated by reverse-phase chromatography (Scheme 26).

**Table 25:** Percent inhibition of GFP fluorescence in cells treated with native, PS or BP siRNA at 25 nM^280^

siRNA duplex^a^	*t*_1/2_ b (h)	EGFP translation; % of inhibition
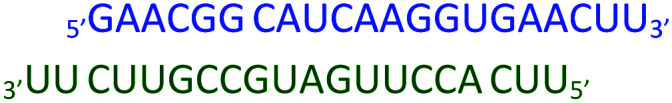	1.4	80
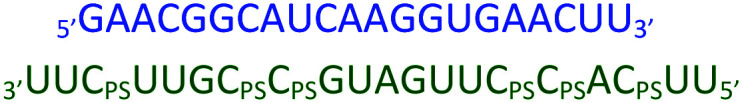	—	85
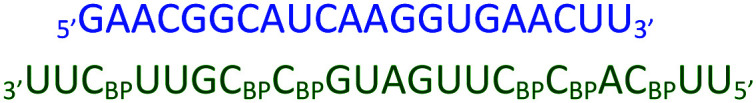	2.2	96
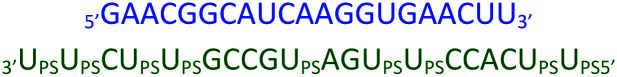	—	45
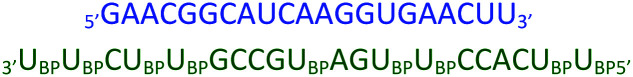	—	70

a PS and BP refer to the phosphorothioate and boranophosphonate internucleoside linkages respectively: sense strand (blue) and antisense strand (green).

b ODN not tested.

Resistance to SVPDE was evaluated for each of the diastereoisomers by comparing them to the natural PO and PS dinucleotides (Table 26).

**Table 26:** Half-life evaluations of different diadenosines against SVPDE and nuclease P1^282^

Dinucleotide^a^	*t*_1/2_	
	SVPDE	Nuclease P1
r(AA)	<1 min	<1 min
r(A_*S*_p_-PS_A)	>48 h	>48 h
r(A_*R*_p_-PS_A)	3 h	10 h
r(A_*S*_p_-BP_A)	10 h	<1 h
r(A_*R*_p_-BP_A)	>48 h	>48 h

a PS and BP refer to the phosphorothioate and boranophosphonate internucleoside linkages respectively.

The *R*_p_-BP isomer was found to be more resistant than the *S*_p_-BP one, thus corroborating observations made in the DNA series. Similarly, with the corresponding isoelectronic configuration, BP dimers appear to be more stable than PS isomers.

An interesting NMR study published by Shaw and Germann provided a rational explanation for the tolerance of the BP linkage to RNase-H.^283^ Indeed, as seen in this review, very few modifications of the phosphodiester linkage allow its activation. ODN containing a single stereospecific modification of pure stereochemistry *S*_p_ or *R*_p_ were synthesised and analysed by NMR. NOESY experiments were particularly useful to establish interactions through space between atoms. Thus they determined the spatial positions of all atoms of the modified linkage within a duplex formed with a complementary ORN. They were able to highlight the spatial orientation of the BH_3_ group according to the stereochemistry of the phosphorus atom. More precisely, in the *S*_p_ configuration, the BH_3_ group points inside the major groove. In contrast, in the *R*_p_ configuration, the BH_3_ group points outside the double helix. This orientation of the BH_3_ group plays a critical role when RNase-H approaches the duplex. In the case of the *R*_p_ isomer, the BH_3_ group prevents access to the docking area of RNase-H, resulting in the loss of cleavage activity. This is probably due to the modification of the steric hindrance and the local charge distribution.

**Scheme 27:**
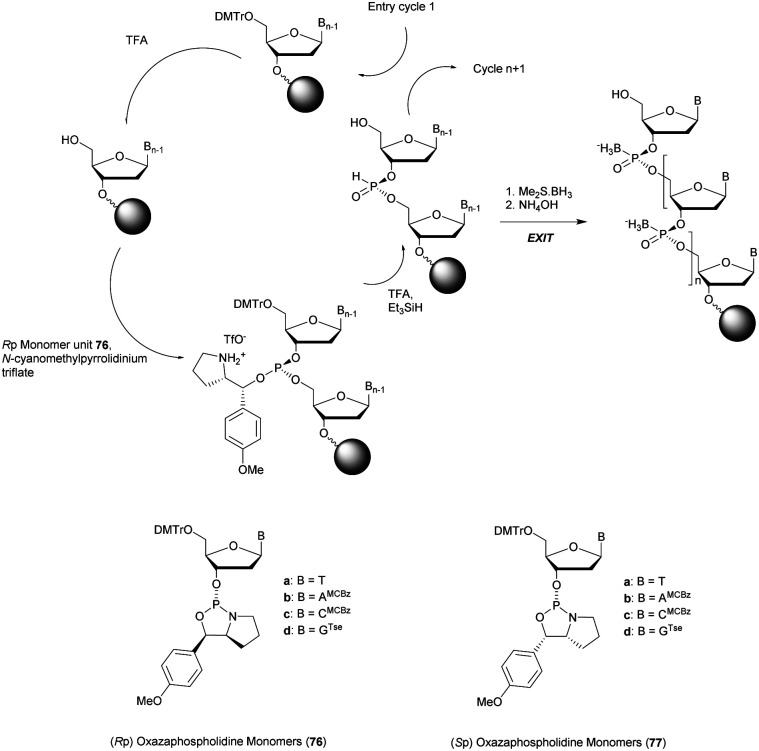
Synthesis cycle of chimeric stereoregular BP/PO-ODN. Structures of the (*R*_p_) **76a–d** and (*S*_p_) **77a–d** oxazaphospholidine monomers.

Recently the group of Wada published the first stereocontrolled synthesis of BP linkages^284^ by adapting the methodology developed for the stereocontrolled synthesis of PS linkages described above (see Section 2.1.1). The main difficulty encountered concerned the reductive properties of boronating reagents. Consequentially, the authors used acid labile protecting groups on nucleobases and oxazaphospholidine monomers that have a 4-methoxyphenyl substituent unit. The syntheses of the stereodefined building blocks **76** and **77** were based on the functionalization of l-proline (α*R*,2*S*) and d-proline (α*S*,2*R*) in 7 steps. The monomers obtained were used for solid supported synthesis of all-*R*_p_-BP and all-*S*_p_-BP 12-mer ODN with very low 2 and 3% isolated yields (Scheme 27).

Then, the differences between all-*R*_p_ and all-*S*_p_-BP-ODN were studied in terms of duplex-formation, nuclease resistance and RNase-H activation. The authors observed that BP-ODN were not able to form stable duplexes with their complementary DNA strand. Melting temperatures were evaluated in the RNA series and a slight destabilization was observed (Δ*T*_m_ ∼ −1.7 °C per modification in the *R*_p_ series and Δ*T*_m_ ∼ −0.7 °C per modification in the *S*_p_ series, Table 27). Thereafter, nuclease digestion experiments were conducted against nuclease P1 (known as (*S*_p_)-specific nuclease) and SVPDE (known as (*R*_p_)-specific nuclease) as demonstrated using PS-ODN (see Section 2.1.1). No kinetic monitoring was performed, but only an analysis after 12 h of incubation. The results confirm the complete digestion of the natural ODN. As shown in Table 27, the all-*R*_p_-BP-ODN was resistant to SVPDE but not to nuclease P1. In contrast, the all-*S*_p_-BP-ODN was resistant to nuclease P1 but hydrolysed by SVPDE. These results corroborate the results previously described for PS ODN. Finally, RNase-H activation experiments were performed. In total accordance with the results obtained previously,^283^ all-*S*_p_-BP-ODN allowed fast and efficient cleavage of the complementary RNA strand, whereas the all-*R*_p_-BP-ODN induced a very low activity of the RNase-H.

**Table 27:** Thermal denaturation studies (*T*_m_ values) of BP-ODN with complementary RNA and their half-life evaluations against nuclease P1 and SVPDE^284^

ODN (5′ → 3′)^a^	*T*_m_ with RNA (°C)	*t*_1/2_ b		RNase-H activation
		Nuclease P1 (h)	SVPDE (h)	
d(GTACTACTACTT)	40.9	—	—	✓
[d(GTACTACTACTT)]-all-*R*_p_-BP	22.7	<12	>12	✓
[d(GTACTACTACTT)]-all-*S*_p_-BP	33.5	>12 h	<12	✗

a BP refers to the boranophosphonate internucleoside linkage.

b Half-lives were not precisely quantified; only one HPLC analysis was performed after 12 h of incubation for each sample.

Boranophosphate ODN have all the required characteristics for therapeutic use along with phosphorothioates. As described in this section, the physico-chemical and biological properties of BP-ODN are now well understood. However, there are still no bioactive molecules using BP as a substitute of the natural PO linkage. The PS-ODN is still favoured by biologists and pharmaceutical companies, probably because PS-ODN are easy to synthetize using standard procedures. The development of new efficient synthetic methodologies may allow renewed interest of medicinal chemists for the BP modification in the future.

#### 3.1.8 Doubly modified internucleoside linkages

##### 3.1.8.1 Carbon–phosphorus–sulphur: methylphosphonothioates (MPS)

**Scheme 28:**
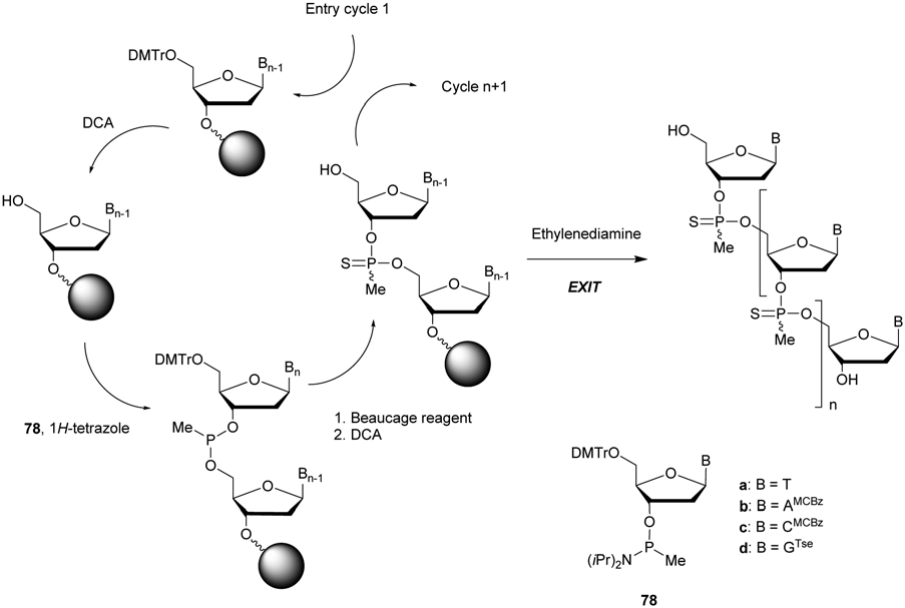
Synthesis cycle of MPS-ODN.

The first methylphosphonothioates (MPS) were synthesised by the group of Caruthers in the late 1980s.^285,286^ They used methylphosphonothioic dichloride as a condensing reagent with 5′-*O*-Tr-thymidine and thymidine, leading to the corresponding dinucleotide methylphosphonothioate in 56% yield over the two steps. This synthesis was improved in the following years but it is only in 1993 that the group of Agrawal reported the first supported automated synthesis of MPS-ODN with an average efficiency of 97% per coupling step.^287^ The supported synthesis relies on the key nucleoside methylphosphonamidites **78** which are condensed under classical conditions using 1*H*-tetrazole in MeCN, followed by an oxidation with the Beaucage reagent (Scheme 28). Alternatively, a conventional coupling according to the phosphoramidite chemistry can be carried out to obtain PO linkages.

Several chimeric MPS-ODN were synthesised using classical and modified cycles of phosphoramidite chemistry (Scheme 28). The stability of the duplexes formed with their complementary RNA strand was evaluated (Table 28). A slight destabilization of the resulting duplexes is highlighted without apparent proportionality according to the number of modifications.

**Table 28:** Thermal denaturation studies (*T*_m_ values) of MPS-ODN with complementary RNA and their half-life evaluations against SVPDE^287^

ODN (5′ → 3′)^a^	*T*_m_ with RNA (°C)	*t*_1/2_ (s)
d(ACACCCAATT-CTGAAAATGG)	51.2	44
d(ACACCCAATT-CTGAAAAT_MPS_G_MPS_G)	47.8	210
d(ACACCCAATT-CTGAAAA_MPS_T_MPS_G_MPS_G)	48	264
d(ACACCCAATT-CTGAAA_MPS_A_MPS_T_MPS_G_MPS_G)	47.1	401

a MPS refers to the methylphosphonothioate internucleoside linkage.

The resistance to SVPDE of these ODN was also quantified (Table 28). The MPS linkage achieved a superior resistance to SVPDE but better results were expected when combining both PS and MP modifications. Although further work on the synthesis of MPS-ODN has been reported,^288–290^ no biological applications have been published to date, presumably because of their surprising low resistance to nucleases.

##### 3.1.8.2 Sulphur–phosphorus–sulphur: phosphorodithioates (SPS)

**Scheme 29:**
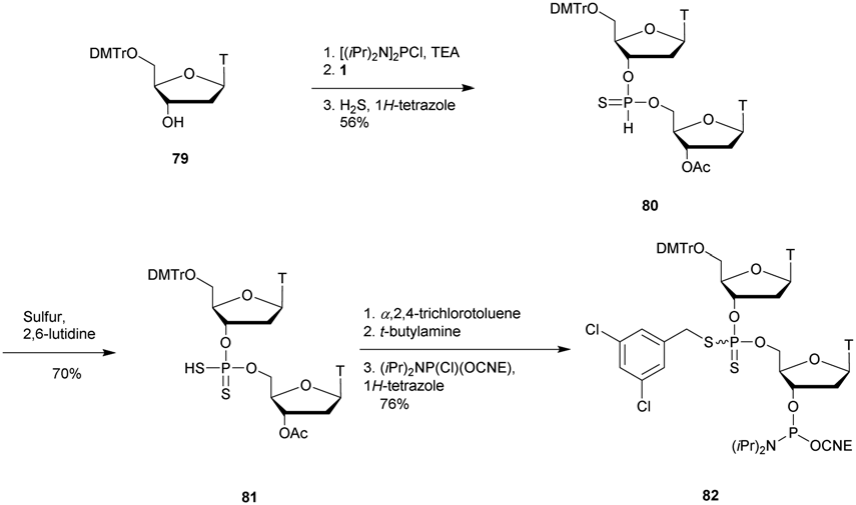
Synthesis of SPS phosphoramidite building block **82**.

The phosphorodithioate (SPS) linkage has been extensively studied because it is isostructural and isopolar with the natural PO linkage. Moreover, contrary to the PS linkage it presents the advantage of being achiral. Its first synthesis was described by the group of Caruthers in 1988.^291^ The developed method paved the way for the synthesis of phosphorothioamidate, alkyl phosphorothioate and phosphorothioate internucleoside linkages.^291^ The synthesis of phosphorodithioate dithymidine started with the condensation of 5′-*O*-DMTr-thymidine (**79**) with bis(diisopropylamino)chlorophosphine. The phosphoramidite generated was then coupled with 3′-*O*-Ac-thymidine, **1**. The first sulfurization was performed with H_2_S gas and the second with elementary sulphur. Dimer **81** was then protected with α,2,4-trichlorotoluene and the 3′-*O*-Ac group was removed, allowing the reaction with bis(diisopropylamino)chlorophosphine, leading to the desired phosphoramidite derivative **82** (Scheme 29). It should be noted that this strategy *via* a phosphoramidite allows the generation of a phosphate diester using conventional oxidation conditions.

Digestion experiments of the deprotected dimer T_SPS_T against SVPDE were carried out. While the natural dinucleotide T_PO_T was fully hydrolysed, under the same conditions the phosphorodithioate was totally stable.

Shortly after, two other publications concerning the synthesis of phosphorodithioate dinucleotides confirmed the total resistance to SVPDE, CSPDE,^292^ BSPDE and nuclease P1.^293^

After extensive efforts by several research groups,^291,294–300^ Caruthers and co-workers developed an efficient solid-supported method for the synthesis of SPS-ODN.^301^ The results are obtained by extension on a solid support of the procedure previously described. The free 3′-OH derivative of **82** was immobilized onto a solid silica support to proceed to the elongation (3′ → 5′ synthesis).

At the same time, the authors synthesised SPS-ODN using their solid supported method and evaluated their ability to activate RNase-H *in cellulo*.^302^ They synthesised several ODN sequences with various lengths and numbers of SPS modifications. First, they showed that the SPS modification only slightly decreased the melting temperature value of the duplexes formed with their complementary natural ODN (Δ*T*_m_ ∼ −0.5 °C per modification). Then, they performed experiments to evaluate the ability of SPS-ODN to activate RNase-H in cell extracts and compared the results with those obtained with natural PO-ODN and PS-ODN. They worked with oligodeoxycytidine targeting the HIV reverse transcriptase. The assay involved carrying out repair synthesis with HIV reverse transcriptase using a primer (15-mer) hybridized with a template (30-mer) in the presence or absence of the SPS-oligodeoxycytidine at increasing concentration for competitive inhibition. The purpose of the experiment was also to evaluate the resistance of the ODN to nucleases present in the cytosol of human cells (Table 29).

**Table 29:** Summary of ID_50_ values for the inhibition of HIV reverse transcriptase^302^

ODN (5′ → 3′)^a^	ID_50_ (μM)
[d(C)_14_]-all-PO	36
[d(C)_14_]-all-PS	1.7
[d(C)_14_]-all-SPS	60
[d(C)_20_]-all-SPS	10
[d(C)_10_]-all-SPS	220
[d(C)_8_]-all-SPS	1.2
[d(C)_4_]-all-SPS	20

a PO, PS and SPS refer to the phosphodiester, phosphorothioate and phosphorodithioate internucleoside linkages respectively.

The results showed that SPS-ODN are strong inhibitors of HIV reverse transcriptase, notably in comparison to their natural and PS analogues (600 and 28 times more active respectively). These encouraging experiments have justified the continuing interest of the scientific community regarding phosphorodithioates.^303,304^ In particular, important efforts have been devoted towards improving their solid supported synthesis,^300,305–308^ or their potential as therapeutic agents.^309,310^

##### 3.1.8.3 Sulphur–phosphorus–nitrogen: thiophosphoramidates (NPS)

**Fig. 16:**
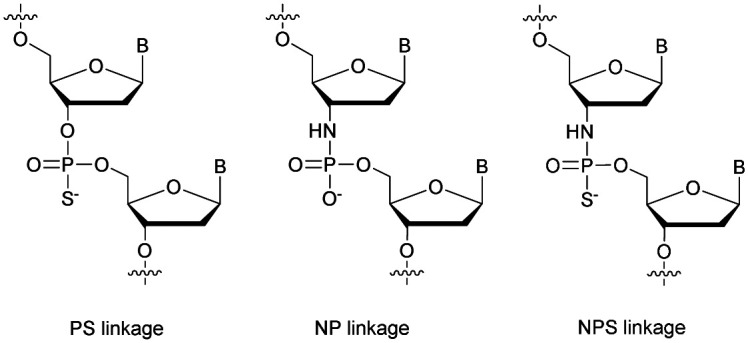
Chemical structures of PS, NP and NPS linkages.

In 1999, the group of Gryaznov introduced the N3′ → P5′ thiophosphoramidate (NPS) linkage^311^ in order to combine the advantages of phorphoramidate and phosphorothioate modifications (Fig. 16).

**Scheme 30:**
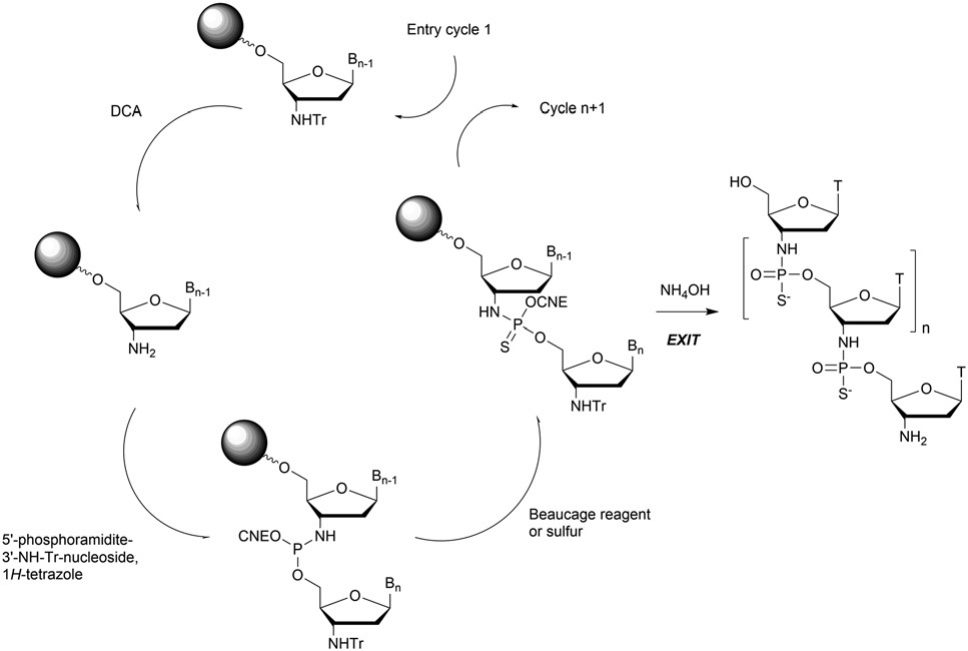
Synthesis cycle of NPS-ODN.

An efficient solid supported synthesis was developed based on a phosphoramidite amine-exchange reaction.^312^ The synthetic strategy used 3′-(Tr)amino-5′-phosphoramidite monomers and classical phosphoramidite chemistry. Surprisingly, elemental sulphur gave better results than Beaucage reagent for the oxidizing step (Scheme 30).

The properties of NPS-ODN were studied. The NPS-d(TAGGGTTAGACAA) demonstrated increased stability in 40% aqueous acetic acid (*t*_1/2_ ∼ 6 h) compared to its NP counterpart (*t*_1/2_ ∼ 0.5 hour). The binding properties with the complementary strand showed that NPS-ODN formed more stable duplexes than natural ODN. The results observed are similar to those obtained with the NP-ODN, showing that the replacement of a non-bridging oxygen atom with a sulphur atom did not affect the binding properties. To the best of our knowledge, no formal study of the nuclease resistance of this modified linkage has been performed. Years later the same group focused on the development of therapeutic ODN based on NPS chemistry targeting the human telomerase in order to treat cancer.^313–318^ The resistance of NPS-ODN to cellular nucleases has been clearly demonstrated with a lipid modified NPS-ODN currently in clinical trial (phase I/II) against cancerous solid tumors in an antagonist strategy, developed by GERON (Imetelstat® or GRN163L). Imetelstat® is a 13-mer lipid-conjugated PNS-ODN complementary to the hTR (RNA chemical structure) component of human telomerase. Imetelstat® binds to the hTR template region at the hTERT (human telomerase reverse transcriptase) active site with high affinity and prevents the recruitment of telomeric DNA. The exploitation of this bioactive NPS-ODN confirms the potential of such a modification for the elaboration of therapeutic ODN able to prevent enzyme recognition of their target even without demonstrated activation of RNase-H.

##### 3.1.8.4 Nitrogen–phosphorus–carbon: methanephosphonamidates (NMP)

**Scheme 31:**
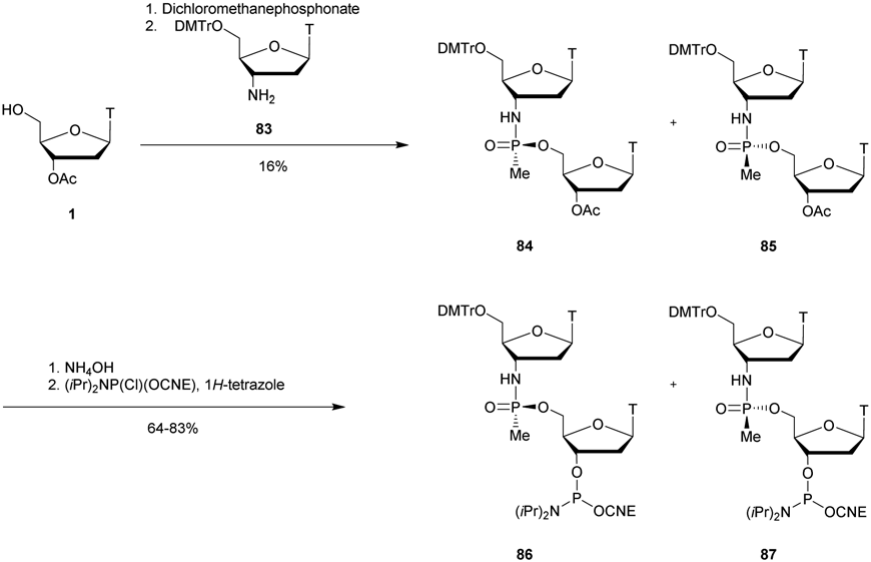
Synthesis of NMP-dithymidine phosphoramidite building blocks **86** and **87**.

The methanephosphonamidate (NMP) internucleoside linkage was introduced in 1998 by the group of Stec.^319^ Diastereomeric dithymidine methanephosphonamidates **86** and **87** (T_NMP_T) were used as building blocks to prepare dodecathymidylates possessing one to four modifications. The latter were synthesised using dichloromethanephosphonate as a key reagent to link 5′-*O*-DMTr-3′-amino-3′-thymidine (**83)** and 3′-*O*-Ac-thymidine, **1** (Scheme 31).

The absolute configuration at the phosphorus atom was based on data reported for the structurally related methanephosphonates. Several polythymidines having one to four NMP linkages were synthesised. The stabilities of the duplexes formed with the complementary DNA strand were studied. An important destabilization of, respectively, −2.4 and −6 °C per modification was observed depending on the absolute configuration of the phosphorus atom. Thereafter, the resistance of the NMP linkage of dithymidine was studied against SVPDE and P1 nuclease. The NMP dithymidine was completely resistant to these two nucleases. Then, 12-mer homothymidylates bearing one to four NMP modifications were incubated with P1 nuclease, CIAP and 3′-exonuclease from human plasma. With 3′-exonucleases from human plasma, the hydrolysis of the ODN occurred from the 3′-end until the enzymes reached the first NMP bond as observed by gel electrophoresis on both NMP absolute configurations without any difference. In all cases, the ODN were digested until the nucleases reached the first NMP linkage which was totally resistant to hydrolysis over 2 h. Moreover, it was shown that alternating PO/NMP linkages drastically slowed down the activity of endonucleases on PO linkages. This is probably due to the hydrophobicity of the NMP linkage chemical structure, which makes the PO bonds less accessible to the enzyme.

Later, Olejniczak *et al.* assigned the absolute configuration at the phosphorus atom using NMR spectroscopy.^320^ This modification of the phosphodiester linkage has not been further studied or exploited for applications in molecular biology.

##### 3.1.8.5 Boron–phosphorus–carbon: boranomethylphosphonates (BMP)

**Scheme 32:**
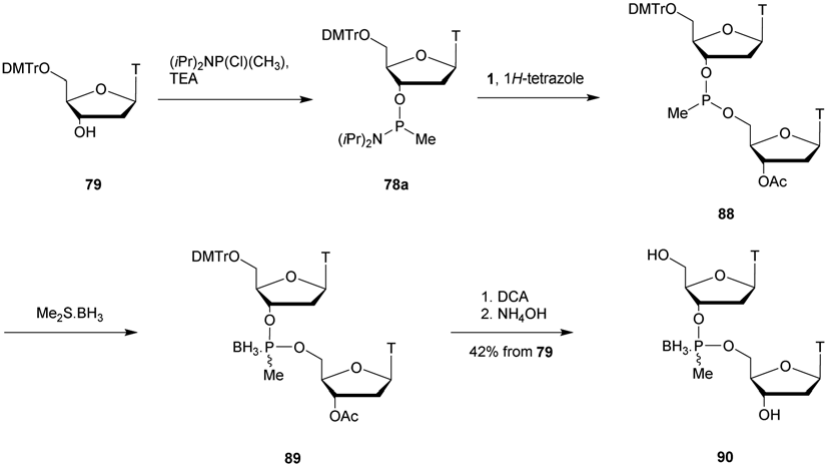
Synthesis of BMP-dithymidine **90**.

In 2001, the group of Shaw developed the synthesis of a dinucleotide modified with a boranomethylphosphonate linkage, **90** (BMP, Scheme 32).^321^ This dinucleotide was obtained from 5′-*O*-DMTr-thymidine (**79**) and 3′-*O*-Ac-thymidine (**1**) that were coupled using 1*H*-tetrazole, leading to the dinucleotide analogue **88**. The latter was oxidized using borane-dimethyl sulfide and finally deprotected to obtain the desired dinucleotide **90** (Scheme 32).

The authors described the boranomethylphosphonate linkage as very stable toward neutral and acidic hydrolyses and extremely resistant toward cleavage by both SVPDE and BSPDE.

**Scheme 33:**
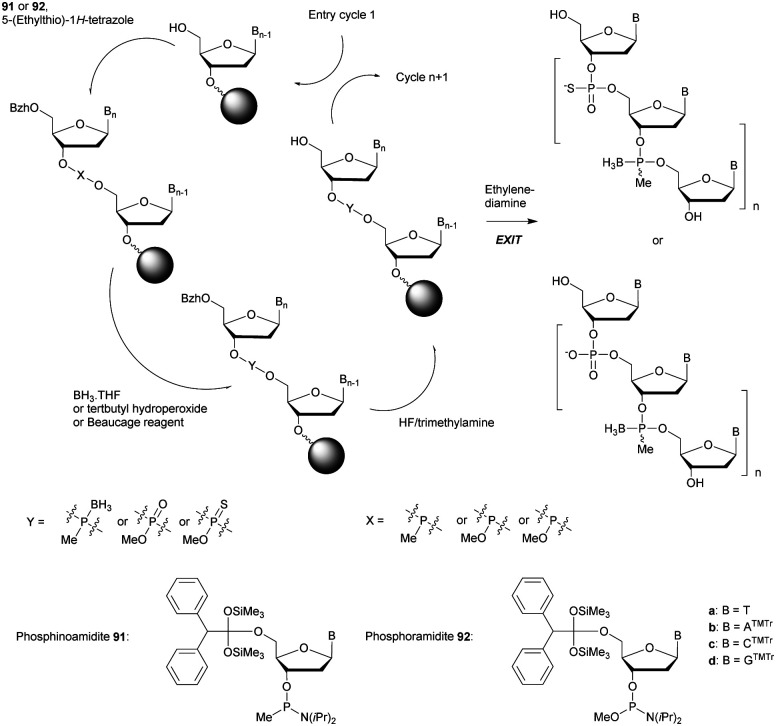
Synthesis cycle of chimeric BMP/PO- or BMP-PS-ODN. Structures of the methylphosphinoamidite building blocks **91** and **92** used for BMP-ODN elongation.^322^

Ten years later, the group of Caruthers developed a solid-supported synthesis of ODN bearing BMP modifications.^322^ The solid-phase synthesis of mixed sequences having methylborane phosphine and PO or PS linkages was achieved using methylphosphinoamidite **91** (synthesised in four steps from unprotected nucleosides) and phosphoramidite **92** synthons (Scheme 33). Note that it was necessary to replace the conventional 5′-*O*-DMTr protecting group with a fluoride labile 5′-*O*-silyl ether (5′-*O*-[benzhydryloxy-bis(trimethylsilyloxy)-silyl], Bzh) and to protect the exocyclic amines with a mild acid-labile TMTr group. Indeed this strategy prevented the reduction of commonly used amide protecting groups to *N*-alkyl or aryl exocyclic amines by borane reagents.^323^ In order to synthetize chimeric ODN, the phosphoramidite chemistry had to be modified (Scheme 33). The strategy employed allows the intermediate synthesis of boranomethylphosphonate, phosphotriester (PT) or thiophosphotriester (PsT) linkages according to the building block used and the oxidation procedure. After deprotection and cleavage from solid supports chimeric BMP/PO- or BMP-PS-ODN were isolated.

Numerous mixed backbone ODN 16-mers bearing methylborane phosphine and phosphate or thiophosphate internucleoside linkages were synthesised. First, the stabilities of the duplexes formed with their complementary ODN or ORN strand were evaluated. The results showed that the modified linkages destabilize all duplexes. However, this destabilization was moderate (Δ*T*_m_ ∼ −1 °C per modification) and did not prevent the formation of duplexes even with a fully modified 16-mer ODN. Then, their resistance to nucleases was tested (Table 30).

**Table 30:** Half-life evaluations of different ODN against SVPDE and CSPDE^322^

ODN (5′ → 3′)^a^	*t*_1/2_	
	SVPDE	CSPDE
d(TAACACGATACGCGAT)	<3 min	<10 min
d(T_BMP_AACACGA-TACGCGA_BMP_T)	60 min	6 h
d(T_BMP_A_BMP_ACACGA-TACGCG_BMP_A_BMP_T)	90 min	12 h
d(T_BMP_A_PS_A_PS_C_PS_A_PS_C_PS_G_PS_A_PS_-T_PS_A_PS_C_PS_G_PS_C_PS_G_PS_A_BMP_T)	7 h	>20 h
d(T_BMP_A_BMP_A_PS_C_PS_A_PS_C_PS_G_PS_A_PS_-T_PS_A_PS_C_PS_G_PS_C_PS_G_BMP_A_BMP_T)	>10 h	>20 h

a BMP and PS refer to boranomethylphosphonate and thiophosphate internucleoside linkages respectively.

The half-life of the natural ODN in the presence of nucleases is about a few minutes. In the case of SVPDE, the presence of a single BMP modification at the 3′-end of a PO-ODN induces a significant increase in the resistance of the ODN (60 min). The authors observed that the SVPDE was able to hydrolyse the BMP linkages, but very slowly. Indeed, a rapid degradation of the internal PO linkages was observed as soon as the BMP cap at the 3′ end was degraded by the exonuclease. As expected, the resistance of the oligomers increased significantly when the internal PO linkages were substituted with PS. Similar results are observed with the less active CSPDE. In another experiment the digestion of a 5-mer ODN (5′-TTT_BMP_TT-3′) bearing a single boranomethylphosphonate linkage did not reveal any SVPDE specificity according to the *R*_p_ or *S*_p_ configuration of the internucleoside linkage. Finally, cellular uptake experiments on HeLa and WM-239A cells have shown that modified (uncharged) ODN penetrate far more easily within cells than natural ODN. Although this modification has certain advantages in terms of nuclease resistance, hybridization capacities and cellular penetration, no antisense or activation of RNase-H experiments has been performed so far.

### 3.2 Non-phosphorus internucleoside linkages

The previous section dealt with modified linkages derived from the natural phosphodiester linkage. Indeed, although multiple modifications have been considered, a phosphorus atom is always present within the linkage. Alternatively, numerous modifications have been developed in which the internucleoside linkage is entirely substituted. This strategy has the advantage of generating achiral linkages while requiring significant synthesis efforts. Such modifications whose resistance to nucleases have been evaluated are reported in this section. Note that in most cases the synthesis of modified dinucleotides was developed by the authors. In a second step, the latter were incorporated using classical solid supported methods and the properties of the resulting ODN were studied, in particular the consequences on adjacent PO linkages for nuclease resistance. Only a few modifications reviewed below have led to new synthetic methods allowing the elaboration of entirely modified ODN (*i.e.* triazole, amide, guanidinium, methylene(methylimino) and carbamate modifications).

#### 3.2.1 Triazole (TR) linkage

The CuAAC, described independently by Sharpless and Meldal in 2002,^215,216^ is the most widely used “click reaction”.^324–327^ Many research groups have studied this reaction in order to effectively functionalize ODN for various applications.^328–332^ This topic has been reviewed by the group of Brown.^333^

**Fig. 17:**
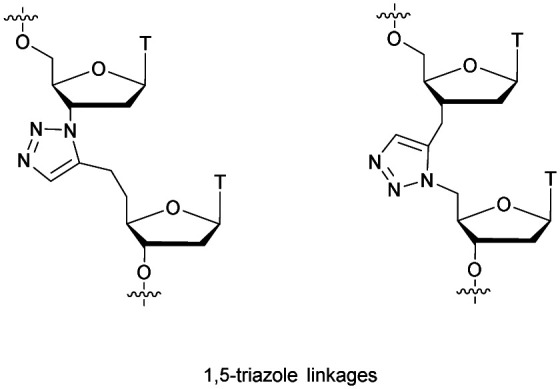
Chemical structures of 1,5-triazole linkages.^334,335^

The efficiency of the CuAAC explains why the triazole (TR) linkage has been one of the most studied non-phosphorus internucleoside linkages. Historically, the first description of a TR linkage between nucleobases dates back to 1997 by Von Matt *et al.* before the use of the Cu(i) catalyzed version of the Huisgen cycloaddition.^334,335^ Triazole modified dithymidines were synthesised *via* the regioselective thermal cycloaddition of a 2-oxoalkylidene triphenylphosphorane with an azide derivative to generate the triazole ring (Fig. 17). The obtained dithymidines were then converted to their phosphoramidite derivatives and incorporated within ODN sequences. The stabilities of the duplexes formed with the ODN or ORN complementary strands were studied.

A significant destabilization of the duplexes formed with the complementary RNA strand was observed although the linkage is electronically neutral and would suggest a stabilizing effect.

After the advent of the CuAAC, numerous studies have described the synthesis of triazole internucleoside linkages from a terminal alkyne and an azide.^336–339^ Among all the publications concerning the TR linkage, only a few have quantified their resistance to nucleases.

For almost a decade the group of Brown has exploited the potential of the CuAAC to replace the natural phosphodiester linkage in various structures such as hairpin and hammerhead ribozyme constructs^340^ or their use for *in vitro* transcription and RNA production.^341^ Moreover, a gene containing the triazole linkage was demonstrated to be functional in *Escherichia coli*.^342^ More recently, the authors described the successful synthesis of a biocompatible triazole-linked gene by one-pot multiple templated ligations.^343^ Finally, they exploited both the CuAAC and SPAAC azide–alkyne cycloadditions to improve the synthesis of single-guide RNA used to program the Cas9 nuclease for CRISPR-Cas9 genome editing compared to enzymatic approaches.^344^

**Scheme 34:**
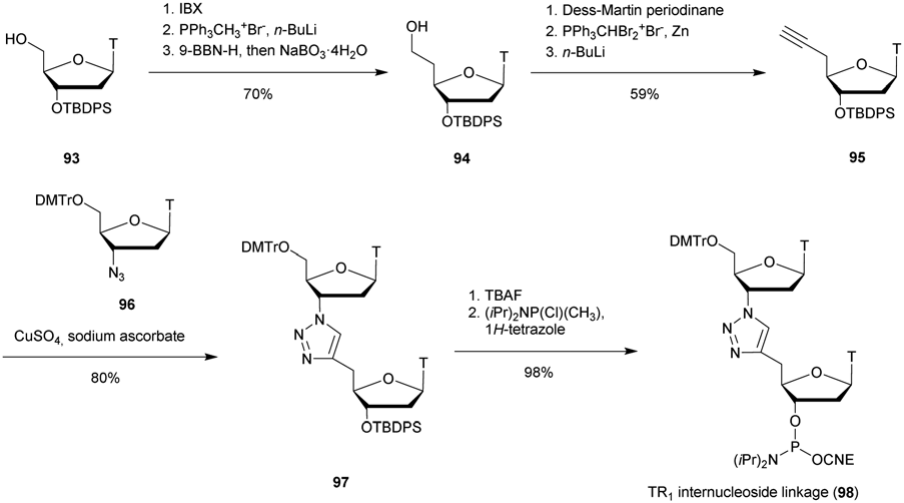
Synthesis of TR_1_-dithymidine phosphoramidite building block **98**.

Isobe *et al.* published the first synthesis of fully modified TR-d(T_10_) ODN^339^ and Varizhuk *et al.* published a few years later the synthesis of chimeric TR/PO-ODN bearing the same TR backbone^345,346^ exploiting a TR-dithymidine building block. The latter was synthesised using 3′-azido-3′-deoxy-5′-*O*-DMTr-thymidine (**96**) and an acetylenic nucleoside (**95**) synthesised in 6 steps from 3′-*O*-(*t*-butyldiphenylsilyl)thymidine, **93** (Scheme 34).^346^ The CuAAC was then implemented to obtain the dithymidine **97** having a triazole (TR_1_) internucleoside linkage (Scheme 34).

The impact of the modification on ODN hybridization was evaluated according to its position within a 17-mer. It was observed that when placed at the 5′-end the modification had little effect (Δ*T*_m_ ∼ −0.7 °C), whereas a strong destabilization was observed when the modification was located at the center (Δ*T*_m_ ∼ −10.4 °C) or at the 3′ end (Δ*T*_m_ ∼ −3.0 °C). This study confirmed the results obtained previously by Von Matt *et al.*^334,335^

**Scheme 35:**
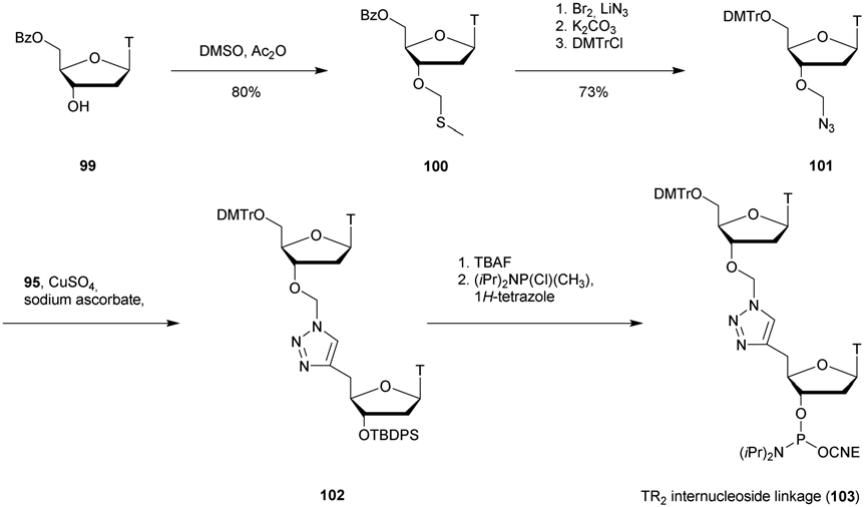
Synthesis of TR_2_-dithymidine phosphoramidite building block **103**.

The resistance of ODN containing a TR1 triazole linkage to nuclease was evaluated using DNase I along with another slightly different one containing 2 more atoms (3′-*O*-CH_2_–, TR_2_).^347^ A very similar strategy was used for the synthesis of TR_2_-dithymidine phosphoramidite. The same acetylenic nucleoside **95** was engaged in a CuAAC reaction with 3′-*O*-(azidomethyl)-5′-*O*-DMTr-thymidine (**101**) obtained in 4 steps from 5′-*O*-Bz-thymidine, **99** (Scheme 35).

Several chimeric ODN were synthesised using supported phosphoramidite chemistry and the thermal stabilities of duplexes formed with a complementary DNA strand were evaluated. Once again a slight destabilization was observed (Δ*T*_m_ ∼ −1.5 °C per modification). Finally, two ODN with three modified linkages were digested by DNase I (Table 31).

**Table 31:** Thermal denaturation studies (*T*_m_ values) of different TR-ODN with complementary DNA and their half-life evaluations against DNase I^347^

ODN (5′ → 3′)^a^	*T*_m_ with DNA (°C)	*t*_1/2_ (min)
d(TTAACTTCTTCACATTC)	50.3	15
d(T_TR1_TAACTTCT_TR1_TCACAT_TR1_TC)^346^	33.0	30
d(T_TR2_TAACTTCT_TR2_TCACAT_TR2_TC)^347^	45.4	30

a TR1 and TR2 refer to the triazole internucleoside linkages of Scheme 34^346^ and Scheme 35^347^ respectively.

The results showed a slowdown of the enzyme activity concerning the ODN containing TR_1_ and TR_2_ linkages compared to the natural PO with half-lives multiplied by a factor 2. Finally, the triazole linkage was found to be well tolerated by polymerases (Taq and Pfu) when used within PCR primers. Modified DNA strands were efficiently copied during PCR with high fidelity. It should be noted that this modification is not supported by all types of polymerase and that the polymerase activity decreases as the number of modifications increases.

**Fig. 18:**
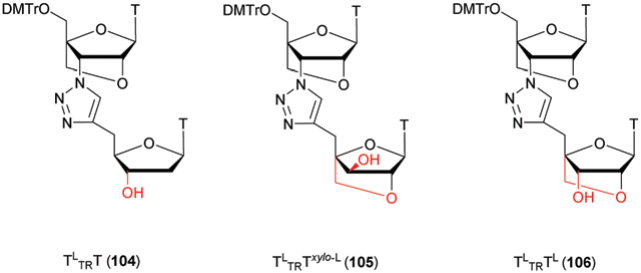
Chemical structures of T^L^_Tr_T (**104**), T^L^_Tr_T^xylo-L^ (**105**) and T^L^_Tr_T^L^ (**106**) TR-LNA-dithymidines.

In 2017 the group of Watts, aware of the weak nuclease resistance and the low binding affinity of triazole ODNs, worked on triazole-linked locked nucleic acids (TR-LNA).^348^ A chemistry similar to the one presented previously (Scheme 34) was implemented in the well documented LNA chemistry. Thus, three LNA-dithymidines possessing the TR_1_ linkage were synthesised: T^L^_Tr_T (**104**), T^L^_Tr_T^xylo-L^ (**105**) and T^L^_Tr_T_L_ (**106**), based on combinations of DNA, *xylo*LNA and LNA (Fig. 18).

**Scheme 36:**
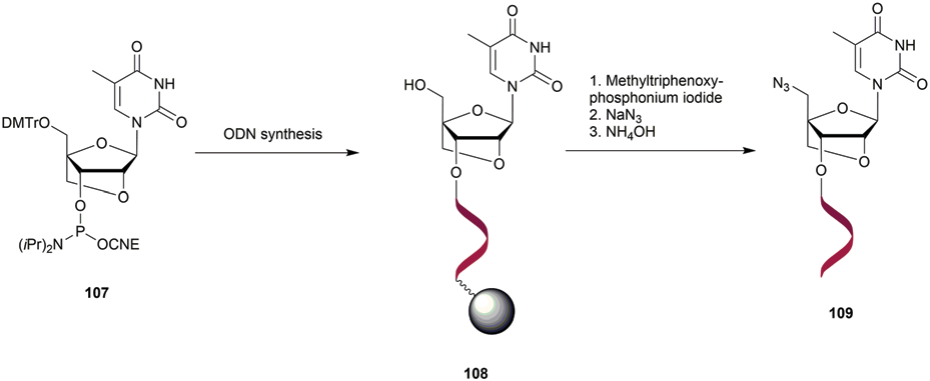
Synthesis of 5′-azido-ODN **109**.

Different ODN bearing one of these modifications were synthesised and their melting temperatures were evaluated when hybridized with their complementary DNA or RNA strand. Concerning the 12-mer ODN modified at the center of the sequence, the duplexes were strongly destabilized in all cases (Δ*T*_m_ ∼ −13 to −26 °C per modification, the reported value in the literature for this triazole linkage is Δ*T*_m_ ∼ −8 °C per modification^339,346^). The intrinsic rigidity of both the triazole linkage and modified carbohydrate strongly reduces the binding affinity relative to triazole alone at the internal position. Thus, the authors studied the effect of a modification at the 3′-end of a 14-mer. Interestingly, the 3′-terminal triazole modification was well tolerated and in fact showed minor duplex stabilization with the complementary ORN (Δ*T*_m_ ∼ +0 to 3 °C per modification, Table 32). Thereafter, the resistances of the ODNs with 3′ modified linkages to SVPDE were evaluated (Table 32). The increase in resistance was significant, particularly in the case of T^L^_Tr_T^xylo-L^ and T^L^_Tr_T^L^ which in addition to the internucleoside linkage presented two consecutive modified deoxyriboses. To further explore the application of TR-LNA linkages, the authors incorporated them into siRNA duplexes. siRNA induced the recruitment of Argonaute protein to silence a targeted RNA. The presence of a phosphate at the 5′-end of the antisense strand was necessary to maintain biological activity. However, the cytoplasmic 5′-exonuclease XRN1 recognized and hydrolysed 5′-phosphorylated RNA including siRNA. Consequentially, stabilizing the 5′-extremity of a siRNA from XRN1 digestion could extend the bioavailability of siRNA. *In vitro* results showed that the three modifications placed at the 5′-end of the antisense strand made the siRNA duplexes resistant to XRN1. Thus, the TR-LNA modified linkage could be helpful for biological applications of gene silencing. Interestingly, a similar study was accomplished at the same time by the group of Brown.^349^ They studied the combination of LNA with their six-atom long triazole linkage whose results were published back to back in the same journal. They focused precisely on the d(C^Me^T) dimer [PO-d(C^Me^T), LNA-d(C^Me^T_L_) and TR-LNA-d(C^Me^_L_T_L_)]. The first two dimers were classically synthesised, using the LNA phosphoramidite version of thymidine for the LNA-d(C^Me^T_L_). The third dimer required the synthesis of an ODN with a final LNA-dT nucleotide whose 5′-OH was converted into an azide (**109**) after elongation by an iodination/azide substitution procedure (Scheme 36).^131,350^

**Table 32:** Thermal denaturation studies (*T*_m_ values) of TR-ODN with complementary DNA or RNA and their half-life evaluations against SVPDE and XRN1^348^

ODN (5′ → 3′)^a^	*T*_m_ with DNA (°C)	*T*_m_ with RNA (°C)	*t*_1/2_	
			SVPDE^b^ (min)	XRN1^c^ (h)
d(TCTCTCTCCCTTTT)	50.5	41.9	<2	<6
d(TCTCTCTCCCTTT^L^_Tr_T)	49.5	42.4	5	>12
d(TCTCTCTCCCTTT^L^_Tr_T^xylo-L^)	49.2	42.7	30	>12
d(TCTCTCTCCCTTT^L^_Tr_T^L^)	49.1	42.9	30	>12

a T^L^_Tr_T, T^L^_Tr_T^xylo-L^ and T^L^_Tr_T^L^ refer to dithymidines with the triazole internucleoside linkages described in Fig. 18.

b Half-lives were estimated from gel electrophoresis performed at different times during incubation.

c No precise half-lives are given because only one gel electrophoresis was performed after 12 h of incubation.

**Scheme 37:**
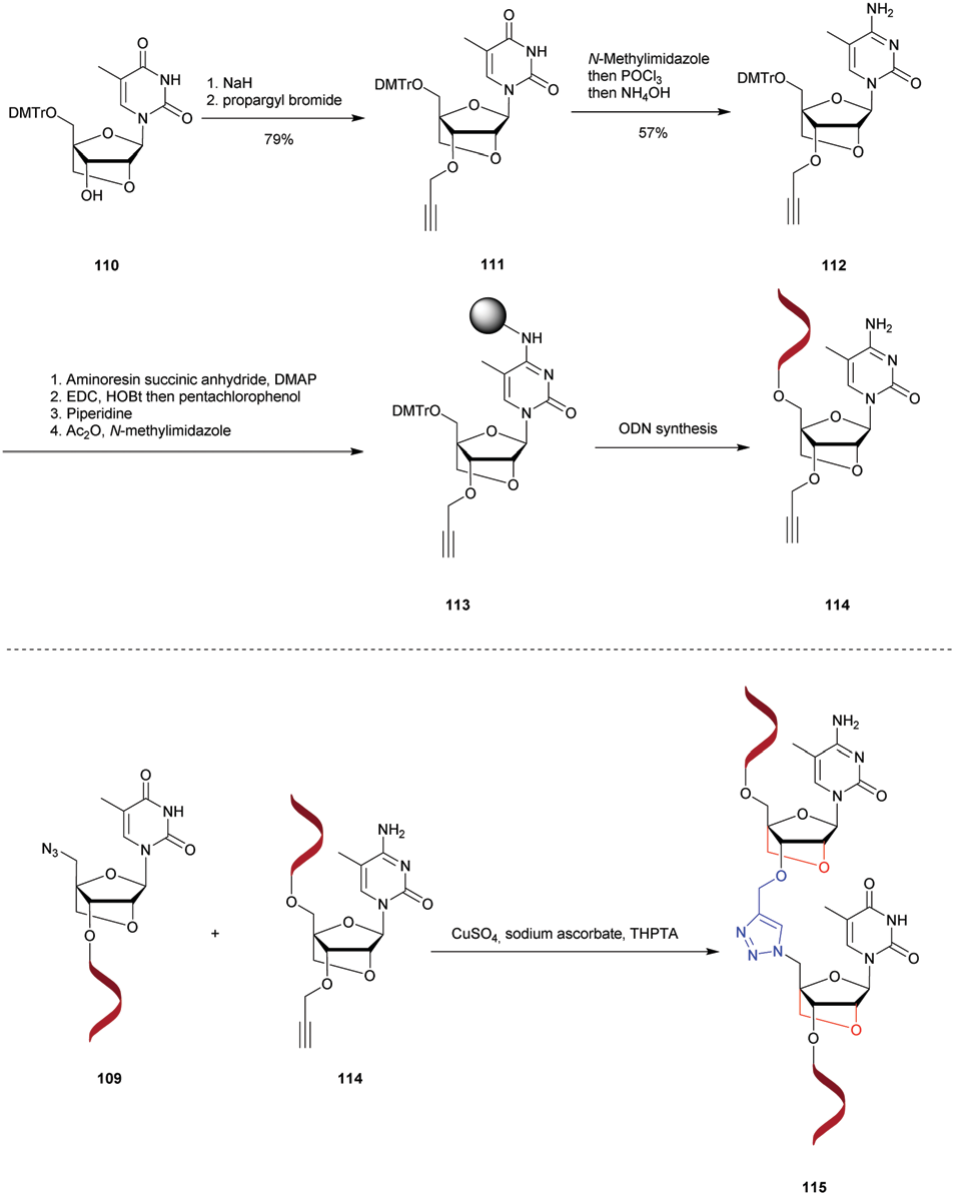
Synthesis of TR-LNA-ODN **115**.

The second partner was obtained from 5′-*O*-DMTr-LNA thymidine (**110**), which first underwent a 3′-propargylation and was then converted to its Me-cytidine analogue **112**. The Me-cytidine (**112**) was attached to a solid support and used for automated ODN synthesis leading to an ODN with a 3′-propargyl LNA cytidine derivative, **114**. A CuAAC reaction was then performed with the two ODN partners **109** and **114**, leading to the desired modified TR-LNA-ODN **115** (Scheme 37).

Thereafter, several 13-mer ODN containing a central ^Me^CT dimer were synthesised. The duplexes formed with their complementary DNA strand are significantly destabilized by the presence of the triazole linkage at the center of the sequence (Δ*T*_m_ ∼ −6 to −12 °C per modification) depending on the number and the position of LNA modifications at the ribose of ^Me^C and/or T. Variations in the *T*_m_ are less significant in the case of DNA/RNA duplexes. Interestingly, the LNA modification stabilizes the duplexes if the modification is present on the 3′ side of the modified dimer (Δ*T*_m_ ∼ +0.1 °C), counterbalancing the destabilization due to the triazole linkage (Table 33).

**Table 33:** Thermal denaturation studies (*T*_m_ values) of TR-ODN with complementary DNA or RNA and their half-life evaluations against SVPDE^349^

ODN (5′ → 3′)^a^	*T*_m_ with DNA (°C)	*T*_m_ with RNA (°C)	*t*_1/2_ b (min)
d(CGACG^Me^CTTGCAGC)	64.2	62.8	<5
d(CGACG^Me^C_Tr_T^L^TGCAGC)	58.2	62.0	—
d(CGACG^Me^C_Tr_TTGCAGC)	55.3	56.6	—
d(CGACG^Me^CT^L^TGCAGC)	67.5	68.9	<5
d(CGACG^Me^C^L^_Tr_TTGCAGC)	52.7	55.5	—
d(CGACG^Me^C^L^_Tr_T^L^TGCAGC)	58.4	62.9	10

a Tr and L refer to the triazole internucleoside linkage described in Scheme 37 and LNA residues respectively.

b ODN not tested.

Thereafter, the authors studied the resistance to SVPDE achieved by this modification to ODN. The latter is fully capable of hydrolysing the modified ODN. However, the enzyme activity is drastically reduced, opening the way in case of multiple modifications to a significant *in vivo* bioavailability increase for the implementation of therapeutic applications.

**Fig. 19:**
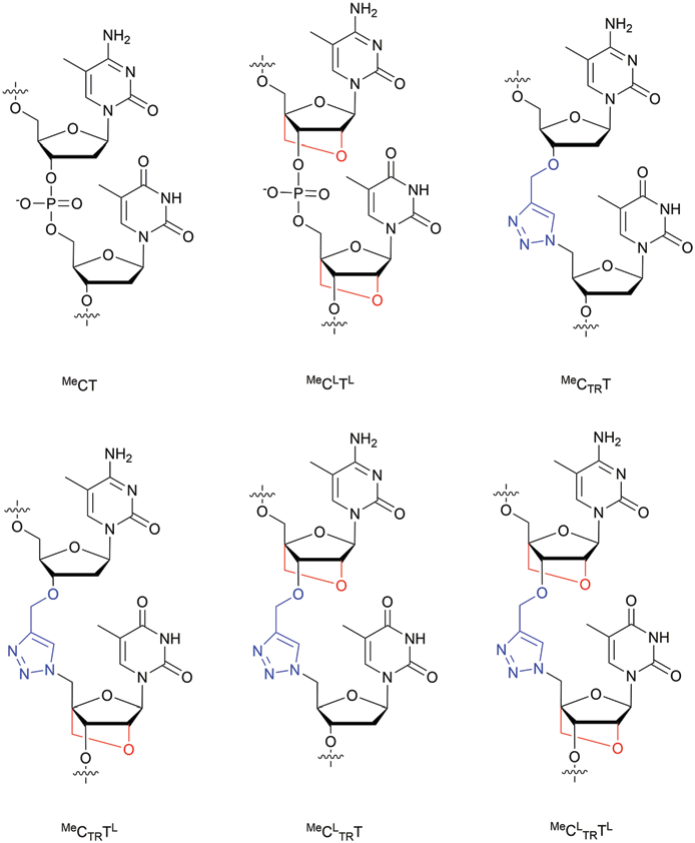
Chemical structures of PO- and TR-DNA and LNA backbones investigated.^351^

The study concerning multiple incorporations of various ^Me^CT dimer within ODN was conducted and published in 2018 (Fig. 19).^351^

Hybridization studies have been undertaken on ODN containing one to four blocks of the dimers described above (Fig. 19). The results previously obtained were confirmed^349^ with a destabilization of the duplexes formed with the complementary DNA or RNA strand for each TR linkage replacing a natural PO. However, the LNA modification surrounding the modified linkage counterbalanced the decrease in affinity and allowed in some cases the formation of more stable duplexes than with the natural ODN (Table 34). In addition, ODN containing multiple modified linkages from ^Me^C_TR_T^L^ and ^Me^C^L^_TR_T^L^ dimers are highly resistant to nuclease degradation thanks to the contributions of both the TR and LNA modifications (Table 34). This effect was observed with SVPDE and FBS on an ODN containing four ^Me^CT dimers. The digestion by SVPDE clearly showed the resistance achieved by the LNA or TR linkage modification. The 13-mer containing four units of ^Me^C^L^_TR_T^L^ was extremely stable (*t*_1/2_ > 8 h, Table 34), while the one having a single modification was rapidly hydrolysed (*t*_1/2_ ∼ 10 min, Table 34). This unambiguously demonstrates the additive effect of multiple modifications concerning the resistance to SVPDE. Regarding FBS, the results showed that the enzymes work differently than SVPDE. Actually, the contribution to the resistance to the nucleases contained in FBS is mainly provided by the modification of the ribose compared to the one provided by the TR linkage. Logically, once again the modified ODN containing four units of ^Me^C^L^_TR_T^L^ is the most stable. Further biochemical and biological studies are required to explain the synergistic effect of multiple modified blocks, but the results highlight the great potential of this modification for antisense applications.

**Table 34:** Thermal denaturation studies (*T*_m_ values) of the TR-ODN with complementary DNA or RNA and their half-life evaluations against SVPDE and FBS

ODN (5′ → 3′)^a^	*T*_m_ with DNA (°C)	*T*_m_ with RNA (°C)	*t*_1/2_	
			SVPDE	FBS (h)
d(^Me^CTCA^Me^CTAT^Me^CTG^Me^CT)	58	56.7	<2 min	1
d(^Me^C^L^T^L^CA^Me^C^L^T^L^AT^Me^C^L^T^L^G^Me^C^L^T^L^)	—	>75	30 min	8
d(^Me^C_TR_TCA^Me^C_TR_TAT^Me^C_TR_TG^Me^C_TR_T)	37.6	39.4	1 h	<4
d(^Me^C_TR_T^L^CA^Me^C_TR_T^L^AT^Me^C_TR_T^L^G^Me^C_TR_T^L^)	45.8	57.8	4 h	6
d(^Me^C^L^_TR_T^L^CA^Me^C^L^_TR_T^L^AT^Me^C^L^_TR_T^L^G^Me^C^L^_TR_T^L^)	48.3	62.3	>8 h	>12

a TR and L refer to the triazole internucleoside linkage described in Scheme 37 and the LNA nucleoside respectively.

#### 3.2.2 Dialkyl sulfide (s) linkage

**Scheme 38:**
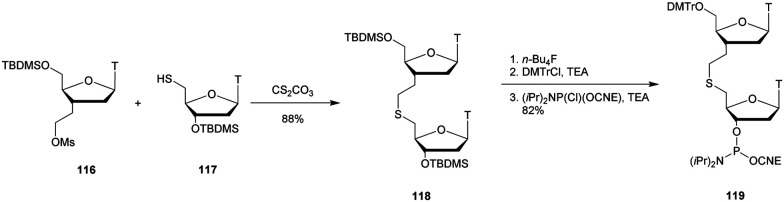
Synthesis of *S*-dithymidine **119** (T_S_T).

In 1993, Kawai *et al.* developed an efficient synthesis of a thymidine dinucleotide analogue having a dialkyl sulfide (s) linkage.^352,353^ They developed the synthesis of 5′-SH functionalized thymidine **117***via* a Mitsunobu reaction using AcSH as a nucleophile. Coupling with the mesylated derivative **116** led to the desired dimer precursor of the dithymidine phosphoramidite **119** (Scheme 38), which was used in an automated supported synthesis.

Several modified ODN were obtained and tested against SVPDE, CSPDE, nuclease S1 and nuclease P1. None of these enzymes were able to hydrolyse the sulfide linkage T_S_T after 48 h of incubation at 37 °C. The 6-mer d(T_s_TT_s_TT_s_T) was degraded to T_s_T dimers by SVPDE, demonstrating once again the ability of this enzyme to “jump” over a modified linkage as is the case with methyl or methylene phosphonate modifications.^174,196^ The CSPDE was not capable of performing such “jump”; therefore its activity was stopped as soon as it encountered a sulfide linkage. Thus, the presence of a dialky sulfide modified internucleoside linkage can provide an effective protection against 5′-exonucleases. It is also possible to use it as a 3′ protective group by preparing several consecutive modified linkages to prevent SVPDE from “jumping” over a single modification as was done for methylphosphonate modification.^180^

#### 3.2.3 Sulfamate (SUL) linkage

**Scheme 39:**
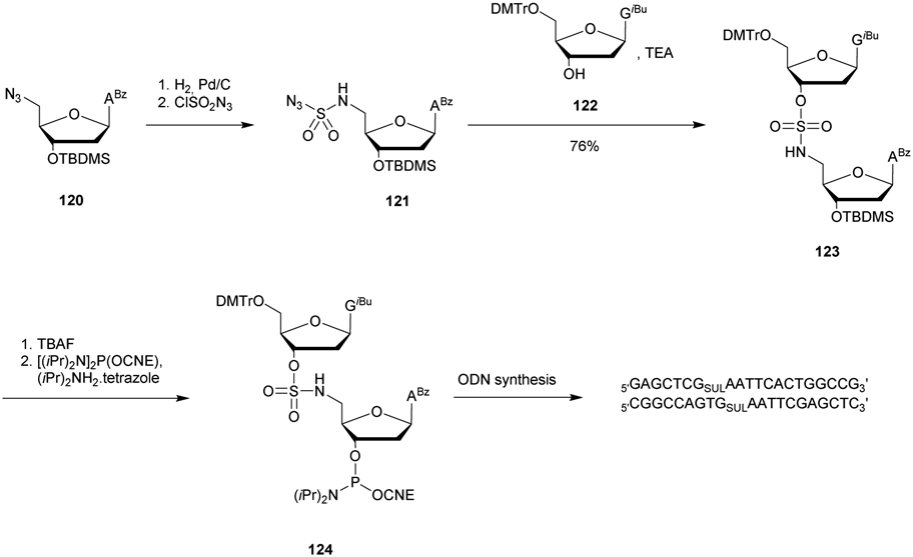
Synthesis of SUL-dithymidine phosphoramidite building block **124** used for ODN elongation.

Huie *et al.* reported in 1992 the synthesis of two complementary ODN comprising the recognition sequence of the EcoR1 enzyme (GAATTC) bearing a sulfamate (SUL) linkage at the cleavage site. The targeted sequence being very specific, it required the synthesis of the SUL-d(GA) dimer.^354^ The synthesis started with the reduction of 5′-azido-*N*^6^-benzoyl-2′,5′-dideoxyadenosine (**120**) using a H_2_ and Pd/C catalytic system. The sulfamoylazide derivative **121** was obtained by reaction with chlorosulfonyl azide. The latter was then condensed with 5′-*O*-DMTr-*N*^6^-iBu-2′-deoxyguanosine (**122**) in the presence of triethylamine, leading to dimer **123**. The SUL phosphoramidite building block **124** was obtained after desilylation with TBAF and reaction of the free alcohol with 2-cyanoethyl *N*,*N*-diisopropylchlorophosphoramidite. **124** was then used in classical supported ODN synthesis to obtain a pair of complementary ODN that formed an EcoR1 restriction endonuclease recognition site (Scheme 39).

The authors studied the hybridization properties of the modified DNA double strand as well as its resistance to EcoR1 and a mixture of SVPDE and CIAP (Table 35).

**Table 35:** Thermal denaturation studies (*T*_m_ values) of SUL-ODN duplexes and their half-life evaluations against EcoR1 and SVPDE (once the modified nucleoside was reached)^354^

Hybridized duplex^a^	*T*_m_ (°C)	*t*_1/2_ b	
		EcoR1 (min)	SVPDE + CIAP
5′-GAGCTCGAATTCACTGGCCG-3′	73	30	—
3′-CTCGAGCTTAAGTGACCGGC-5′			
5′-GAGCTCG_SUL_AATTCACTGGCCG-3′	70	>60	>60
3′-CTCGAGCTTAA_SUL_GTGACCGGC-5′			

a SUL refers to the sulfamate internucleoside linkage.

b ODN not tested.

While the natural double strand had a half-life of about 30 min, the analogue containing sulfamate linkages at the hydrolysis site remained perfectly stable against EcoR1. In addition, a resistance experiment of the sulfamate linkage was performed with a mixture of SVPDE and CIAP. While all the PO linkages were hydrolysed, the SUL linkage was found to be totally resistant with the recovery of the sulfamate dinucleotide after 60 min of incubation. The high resistance observed for the sulfamate internucleoside linkage towards EcoR1, SVPDE and CIAP could lead to interesting biological applications after further investigations.

#### 3.2.4 Boronate (bn) linkage

Boronic acids are well known for their ability to react with *cis*-1,2 or 1,3-diol functions, resulting in the reversible formation of cyclic boronic esters in aqueous medium.^355^ Our group explored the replacement of the natural internucleoside phosphodiester linkage with a boronate ester (bn).^356,357^ Indeed, such a linkage presents a strong electronic analogy with its natural PO counterpart. The synthesis of boronothymidine (dT_bn_) relies on the hydroboration of an alkyne derivative using diisopinocampheylborane ((ipc)_2_BH). Similar strategies were implemented in order to access dC_bn_ and dG_bn_, but dA_bn_ required a different route *via* a cross-metathesis reaction.^356,357^ The phosphoramidite derivative was prepared by protection of the boronic acid in the form of a pinacol borane ester, followed by standard phosphitylation for solid supported ODN elongation.^358^

**Scheme 40:**
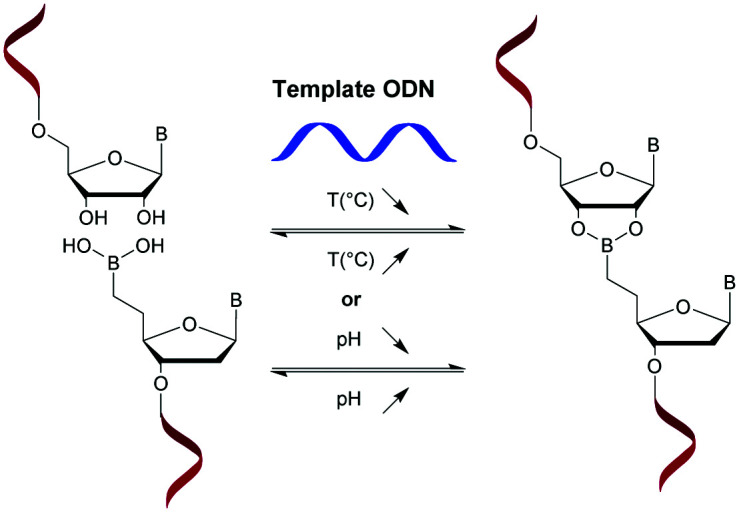
Temperature and pH driven boronic ester chemical ligation.

Following this work, we described a DNA- and RNA-templated ligation system in which the terminal 5′-phosphate of an ODN was replaced with a boronic acid. Hence, in the presence of a 3′-ended ribonucleotide partner, the dynamic and reversible formation of a boronic ester internucleoside linkage provided an efficient means to covalently link the two ODN partners (Scheme 40).^358–360^ The linkage can be reversibly directed by controlling the pH, temperature or anion concentration. Melting temperature experiments showed that the presence of the bn linkage induced a strong destabilization compared to the analogous non-modified duplex. However, the bn linked short ODN are more stable than their nicked counterparts due to the formation of covalent boronic ester linkages.

This concept has recently been applied for the development of an efficient non-enzymatic and sequence-specific DNA- and RNA-templated polymerization of short oligomers resulting in the formation of multiple boronate internucleoside linkages.^360^ To fully exploit the biological potential of boron-based ssDNA and dsDNA, it appeared necessary to evaluate their respective resistance to nuclease degradation.^361^ Single and double stranded DNA were tested against different 3′- and 5′-end exonucleases (Exo I, Exo III, SVPDE and CSPDE). The results obtained demonstrated a high resistance of borono-based ODN to nuclease degradation (Table 36). Concerning ssODN, exonuclease I rapidly digested both modified and natural ODN. However, the bn 5′-end modification induced an increased resistance to SVPDE and a total resistance to CSPDE (probably because it required a hydroxyl group at the 5′-end). Then, the resistance of dsODN was studied. The presence of the 5′-end boronic acid modification induced an important increase in resistance to exonuclease III, SVPDE and CSPDE compared to the non-modified strand. We also studied the impact of the presence of a boronate internucleoside linkage at pH 8.5. High resistance to hydrolysis was observed against all the nucleases compared to the natural duplex. All these results indicated that ss and ds boronic acid-modified DNA cause significant inhibition of various nuclease enzymes. This property has been so far unique among all the modifications described in the literature. Whereas a modified internucleoside linkage generally prevents or slows down nuclease activity once reached, the boronic acid moiety provides an overall protection to the entire ODN. Likewise, the presence of a boronate linkage at the center of ODN sequences dramatically increases the global resistance of the ODN to nuclease degradation (especially 3′-exonucleases).

**Table 36:** Half-life evaluations of bn-ss and bn-dsODN against Exo I, Exo III, SVPDE and CSPDE^361^

ODN (5′ → 3′)^a^	*t*_1/2_ b			
	Exo I (ss)	Exo III (ds)	SVPDE (ss/ds) (min)	CSPDE (ss/ds)
d(T_bn_GAATACAAATT)	<5 min	—	180	>5 d
d(TGAATACAAATT)	<5 min	—	<5	<5 min
d(T_bn_GAATACAAATT)/d(TTTGTATTCAGCCCATATCTT)	—	>5 d/>5 d	180/75	>5 d/24 h
d(TGAATACAAATT)/d(TTTGTATTCAGCCCATATCTT)	—	30 min/30 min	<5/<5	<5 min/<5 min
d(T_bn_GAATACAAATT)/d(GATATGGG)rC/d(TTTGTATTCAGCCCATATCTT)	—	>5 d/>5 d/>5 d	180/180/75	>5 d/>5 d/24 h
d(TGAATACAAATT)/d(GATATGGG)rC/d(TTTGTATTCAGCCCATATCTT)	—	30 min/30 min/30 min	<5/<5/<5	<5 min/<5 min/<5 min

a bn refers to the boronate internucleoside linkage.

b ODN not tested.

These results eventually led to the development of a new label-free enzyme-assisted fluorescence-based method for single mismatch detection based on the addition of SYBR Green I.^361^ This dye exhibits a large fluorescence enhancement upon binding to dsDNA. Indeed, with mismatched target strands being digested much more rapidly, addition of SYBR Green I after 30 min of incubation induced a 17-fold intensity enhancement in the presence of the wild-type complementary strand. Consequentially, the properties of 5′-end boronic acid open the way to a variety of potential applications for the detection and control of genes both *in cellulo* and *in vivo*.

In 2016 we demonstrated that 5′-boronic acid modified ODN could also inhibit RNase-H activity in a bn-ODN/RNA duplex.^362^ We then exploited this property to develop an original system allowing the chemoselective detection of endogenous and exogenous peroxynitrites in RAW264.7 cells. The method relies on the recovery of RNase-H activity upon oxidation of the boronic acid moiety and hence the increase in fluorescence due to the termination of FRET between a fluorophore and a quencher.

The properties of the bn linkage concerning its reversibility, enhanced nuclease resistance and inhibition of RNase-H activity make it a very interesting modification for sensing applications.

#### 3.2.5 Piperazine (PI) linkage

**Scheme 41:**
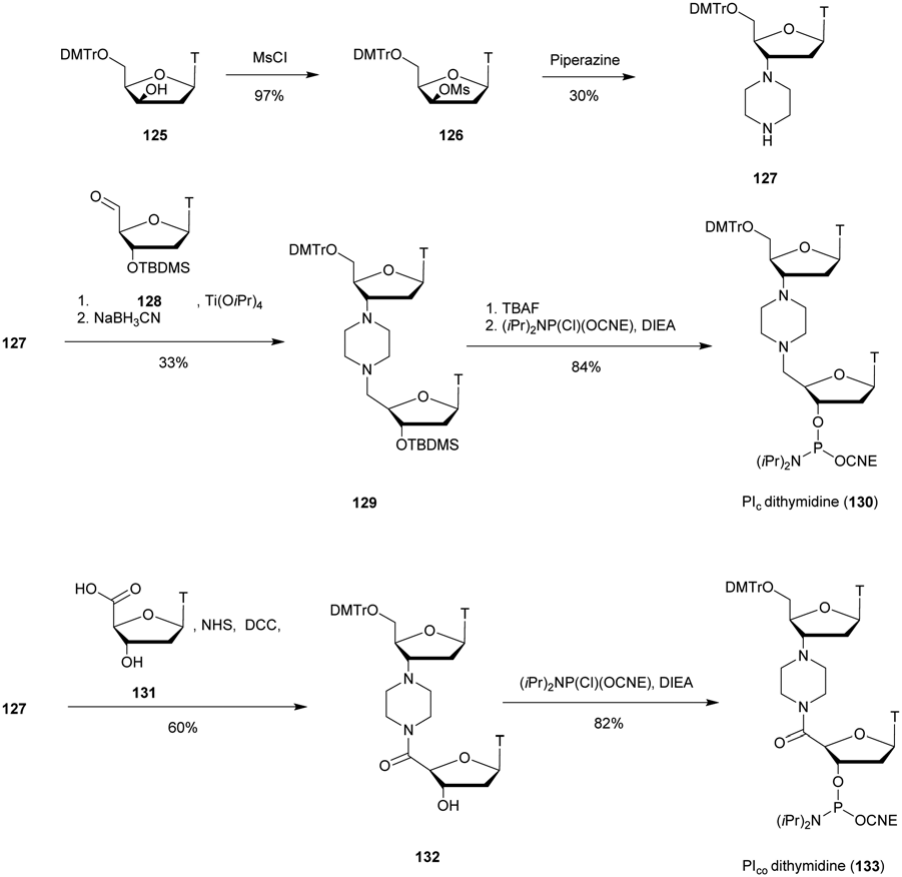
Synthesis of piperazine-dithymidine phosphoramidite building blocks **130** and **133** (PI_c_ and PI_co_).

In 1995 Petersen and Wengel described the synthesis of thymidine dinucleotides in which a piperazine (3′-(N(CH_2_CH_2_)_2_N)-CH_2_-4′ (PI_c_) and 3′-(N(CH_2_CH_2_)_2_N)-CO-4′ (PI_co_) replaced the natural PO linkage.^363^ Nucleoside **125**^364^ was mesylated in pyridine and substituted with piperazine, leading to derivative **127**. The 3′-*O*-TBDMS-5′-aldehyde-thymidine (**128**) and 4′-carboxylic acid-thymidine (**131**) were prepared from thymidine as previously reported.^365,366^ Dimer **129** was synthesised from **127** and **128** by reductive amination using sodium cyanoborohydride and titanium tetraisopropoxide in toluene followed by TBAF desilylation in THF. Dimer **132** was obtained from **127** and **131** by DCC/NHS coupling. The phosphoramidite derivatives **130** and **133** were obtained by reaction with 2-cyanoethyl *N*,*N*-diisopropylchlorophosphoramidite (Scheme 41).

Both phosphoramidites **130** and **133** were incorporated within an ODN on an automated DNA-synthesizer. This alteration of the internucleoside linkage caused a strong destabilization of the duplexes formed with a complementary DNA strand of about −11 °C per modification for PI_c_ and −2 °C for PI_co_. The loss of stability can be explained by the greater rigidity of the linkage as well as its overall shorter length compared to the natural PO linkage, despite the greater number of atoms as mentioned by the authors. These analogues were then evaluated against SVPDE digestion. When the modified dithymidine was placed at the center of the strand, no resistance increase of the strand was observed. However, when the modification was placed at the 3′-end of the ODN, an increase in resistance was observed by a factor of eight to nine. The lower thermal stability of the duplexes compared to natural ODN and their poor resistance to nuclease demonstrate the low potential of the Pi_c_ containing ODN. Although the PI_co_ piperazine linkage only slightly destabilized duplexes, it was also sensitive to nuclease digestion. The study of several successive modifications at the 3′-end of an ODN could be interesting in order to demonstrate a possible important increase in resistance of the ODN to SVPDE and 3′-exonucleases.

#### 3.2.6 Guanidine (GUA) linkage

**Scheme 42:**
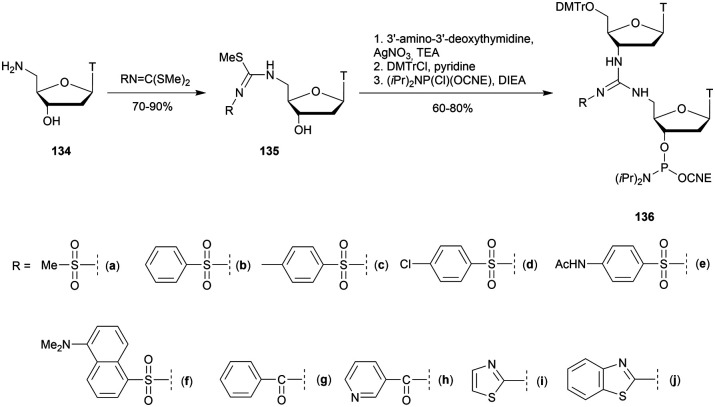
Synthesis of *N*-substituted guanidine-dithymidine phosphoramidite building blocks **136a–j**.

The replacement of the natural phosphodiester linkage with *N*-substituted guanidine (GUA) groups was first described by Herdewijn and co-workers in 1993.^367^ These derivatives were obtained by reacting the unprotected 5′-amino-5′-deoxy-thymidine **134** with different *S*,*S*-dimethyl-*N*-substituted dithiocarbonimidate reagents, leading to the corresponding *N*-substituted isothiourea **135**. Treatment with 3′-amino-3′-deoxythymidine allowed access to thymidine dimers with different *N*-substituted guanidine linkages (Scheme 42). After tritylation and phosphitylation of the 5′ and 3′ positions, respectively, these dimers **136a–j** were incorporated into ODN sequences *via* a standard phosphoramidite method on a DNA synthesizer.

Several homothymidylates were synthesised, including a series possessing at the center of the strand a single modified guanidine linkage. Thermal denaturation studies of the duplexes formed with their complementary DNA or RNA strands were performed. The results indicated a slight destabilization of the duplexes formed with a complementary DNA strand (a few degrees per modification). However, with a complementary RNA strand a strong destabilization between −5 and −10 °C per modification was observed. The resistance to SVPDE of several modified ODN bearing the mesyl substituted guanidine dithymidine was studied (Table 37).

**Table 37:** Thermal denaturation studies (*T*_m_ values) of GUA-ODN with complementary DNA or RNA and their half-life evaluations against SVPDE (absolute time not given)^367^

ODN (5′ → 3′)^a^	*T*_m_ with DNA^b^ (°C)	*T*_m_ with RNA^b^ (°C)	Rel *t*_1/2_^c^
d(T)_17_	43	—	1
d((T_GUA_T)_8_T)	31.5	—	20.4
d(TCTCTCTCTCTTTTT)	46.3	—	1
d(TCTCTCTCTCT_GUA_TT_GUA_TT)	44.8	—	4.1
d(T)_13_	33.2	30.2	1
d(T_GUA_TTTTTTTTTT_GUA_TT)_13_	31.7	—	1.6
d(TTTTTT_GUA_TTTTTTT)_13_	—	25.3	1.4

a GUA refers to the mesyl substituted guanidine internucleoside linkage.

b ODN not tested.

c Rel *t*_1/2_ = *t*_1/2_ modified ODN/*t*_1/2_ unmodified ODN.

The replacement of one PO linkage at the 3′-end of the ODN with a GUA linkage increased the resistance to SVPDE by about 1.6 times. The protection is more efficient if there are two consecutive modified dimers (4.1 times more stable). Moreover, an additional effect with a strong increase in resistance to SVPDE was observed in the case of ODN with alternating PO and GUA linkages (about 20.4 times). All ODN were degraded to the intact dimers, showing the total resistance of the GUA internucleoside linkage and demonstrating once again the ability of the SVPDE to ignore a resistant linkage and to “jump” over it to continue its hydrolytic activity. Thus, several consecutive GUA modified linkages could make ODN totally stable against SVPDE and therefore useful as ODN 3′-end protective groups for biological applications.

**Scheme 43:**
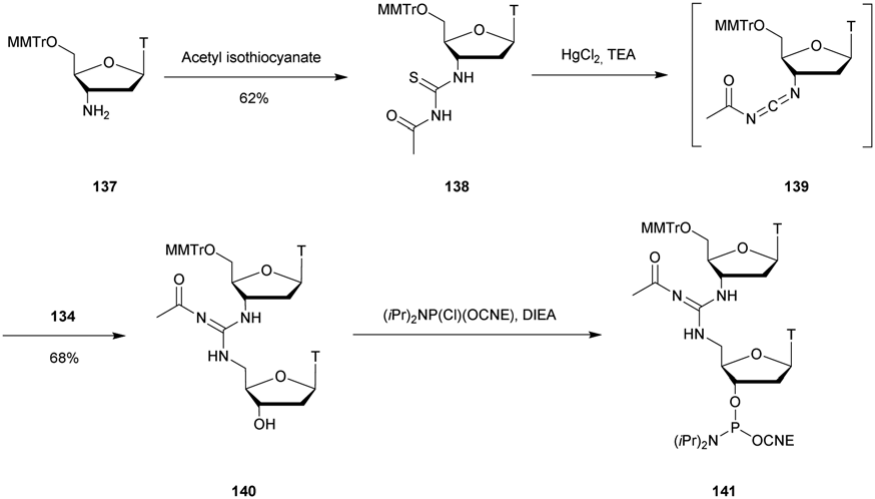
Synthesis of acetylguanidine-dithymidine phosphoramidite building block **141**.

The group of Bruice has studied for years different aspects of the cationic internucleoside guanidinium linkage.^368–374^ In 1998, a specific paper reported the nuclease resistance induced by this modified linkage.^375^ Thus, a mixed backbone 18-mer ODN was synthesised from a GUA modified dithymidine. The latter was obtained starting with 5′-*O*-MMTr-3′-amino-3′-deoxythymidine (**137**) that reacted with acetylisothiocyanate in DCM. Then, an intermediate carbodiimide (**139**) was synthesised by treatment with Hg(ii) in the presence of TEA. Thereafter, the carbodiimide **139** was coupled with 5′-amino-5′-deoxythymidine (**134**) to generate acetyl protected guanidinium dithymidine, **140**. Finally, treatment with *N*,*N*-diisopropylchlorophosphoramidite gave access to the phosphoramidite building block **141** (Scheme 43).

ODN were then synthesised using standard conditions of supported oligonucleotide elongation. The stability of the duplexes formed with the complementary strands in the DNA and RNA series was evaluated. An important effect related to the ionic strength of the aqueous solution was demonstrated. Indeed, a decrease in the ionic strength allowed the positive charge of the guanidinium to form a stable ion pair with the opposite phosphate of the complementary strand. In the case of natural duplexes, the low cation concentration increases the electrostatic repulsion of the phosphate negative charges of the strands. Thereafter, the resistance of the ODN to exonuclease I was evaluated. While the natural ODN had a half-life of 30 min, the GUA-ODN with several 3′-end modifications were completely resistant to exonuclease I (3′-exonuclease activity) during the course of the experiment even at high ionic strength (*i.e.* 50 mM KCl). The GUA-ODN carrying only a single modification at the center of the strand was partially hydrolysed from the 3′-end until the GUA linkage was reached. This demonstrates the high resistance of the GUA linkage to this nuclease. In the context of an *in vivo* use, the presence of a GUA modification at the 3′-end could protect it from certain exonucleases, although the very active SVPDE has not been tested. Years later, Bruice and co-workers described the solid-phase synthesis (3′ → 5′ direction) of fully modified GUA-ODN by adapting their method using mercury salts.^374^ Recently a new method for synthesizing GUA-ODN on solid supports has been published by Mirkin and co-workers.^376,377^ It exploits iodine as a mild and inexpensive coupling reagent and therefore avoids the use of toxic mercury salts. This work demonstrates the interest that this modified backbone first described almost 30 years ago still arouses today.

#### 3.2.7 Methylene(methylimino) (MMI) linkage

**Scheme 44:**
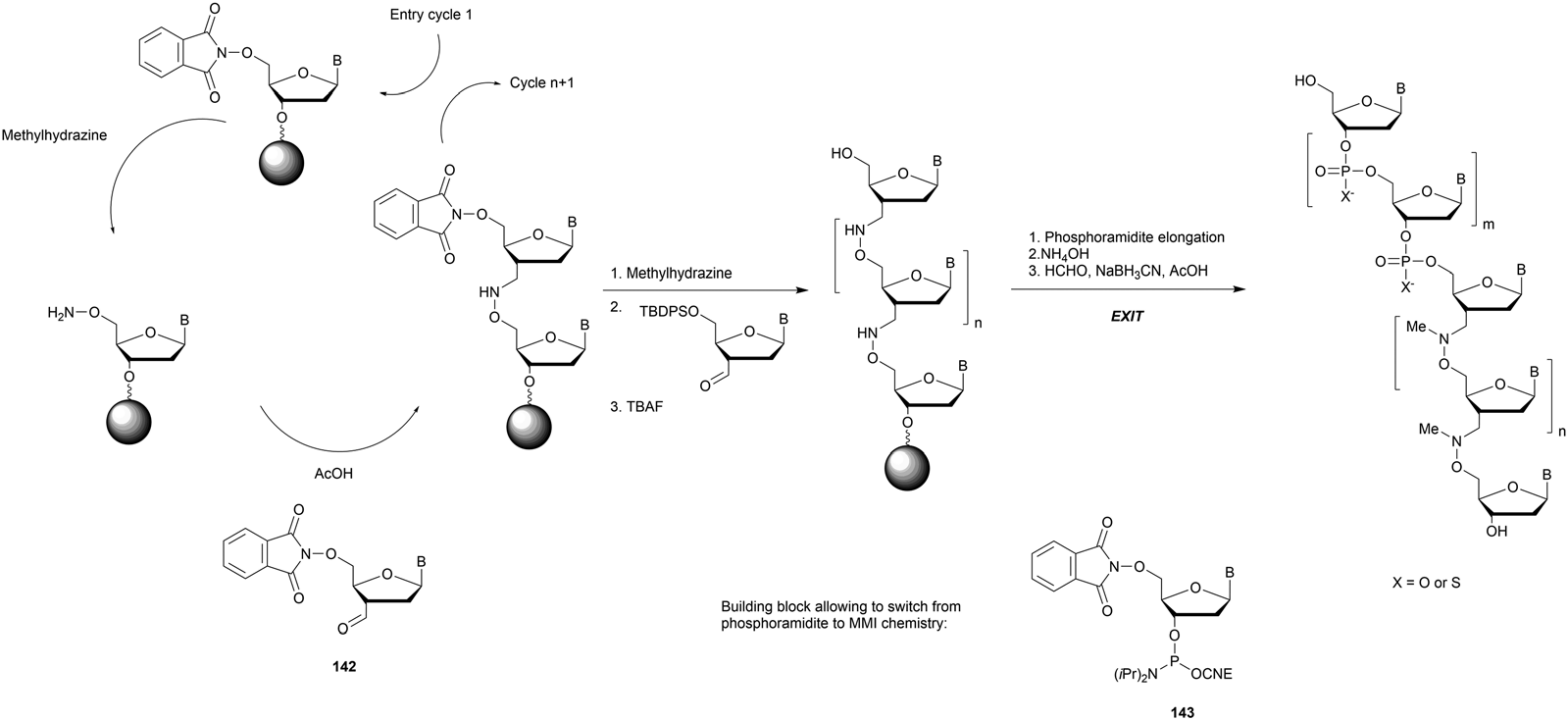
Synthesis cycle of chimeric MMI/PO-ODN.

In 1992, some of us were involved in the replacement of the anionic PO linkage with a neutral methylene(methylimino) (MMI, 3′-CH_2_NH(Me)OCH_2_-5′) linkage.^378^ A dithymidine phosphoramidite building block was synthesised and used for the elongation of ODN using a classical phosphoramidite methodology. The key step relies on the condensation of 5′-aminoxy-5′-deoxy-3′-*O*-TBDPS-thymidine and 3′-formyl-3′-deoxy-5′-*O*-tritylthymidine under acidic conditions. Alternatively, the desired dimer can be obtained through the stereoselective radical dimerization of 5′-*O*-Tr-3′-deoxy-3′-iodothymidine and 5′-*O*-(methyleneamino)-3′-TBDPS-thymidine.^379^ Several ODN sequences were synthesised using phosphoramidite chemistry. Hybridization studies indicated that the MMI linkage has remarkably little effect on the stability of the duplexes formed between the ODN and their RNA complementary strand (Table 38). Nuclease resistance studies were performed by incubation in HeLa cellular extracts or FCS. Polymodified ODN exhibited high resistances to cell extracts with a half-life of 16 h (30 min for natural ODN) and the 3′-capped ODN showed good stability in FCS that contains mostly 3′-exonucleases. A few years later, MMI-ODN comprising consecutive MMI linkages were synthesised using a solid supported synthesis.^380^ Indeed the MMI linkage allows the elaboration of fully modified or chimeric MMI/PO- or MMI/PS-ODN thanks to the specific building blocks **142** and **143** designed. It allows switching from MMI synthesis to classical phosphoramidite chemistry and *vice versa* (Scheme 44).

**Table 38:** Thermal denaturation studies (*T*_m_ values) of MMI-ODN with complementary RNA and their half-life evaluations against HeLa cellular extracts and FCS^378^

ODN (5′ → 3′)^a^	*T*_m_ (°C)	*t*_1/2_ b	
		HeLa cellular extracts	FCS (h)
d(GCGTTTTTTTTTTGCG)	50.2	30 min	—
d(GCGT_MMI_TT_MMI_T-T_MMI_TT_MMI_TT_MMI_TGCG)	50.8	16 h	—
d(CGACTATGCAATTTC)	44.1	—	—
d(CGACTATGCAATT_MMI_TC)	43.6	—	14

a MMI refers to the methylhydroxylamine internucleoside linkage.

b ODN not tested.

A tetramer bearing 3′ modified linkages was found to be fully resistant to SVPDE and nuclease S1. The ability of SVPDE to “jump” over modified internucleoside linkages described previously on several modifications is in any case prevented by the successive MMI linkages. Thereafter, analogues of phosphorothioate modified PS-3521 antisense ODN directed against the PKC-alpha protein (PKCα is associated with the growth and invasion of numerous cancers) coding sequence (active at 100 nM) were synthesised. They inhibited protein translation to the same degree as the PS-ODN but with the advantage of being more resistant to nucleases. This modification has numerous advantages such as an achiral and neutral backbone, a high affinity toward RNA and strong nuclease resistance. Finally, its usefulness was demonstrated as a capping sequence for the synthesis of bioactive gapmers. Noteworthily, entirely modified MMI-ODN exhibited poor water solubility due to their neutral backbone.

#### 3.2.8 Amide (AM), urea (UR), morpholino phosphoramidate (MO) and morpholino phosphorodiamidate (PMO) linkages

**Scheme 45:**
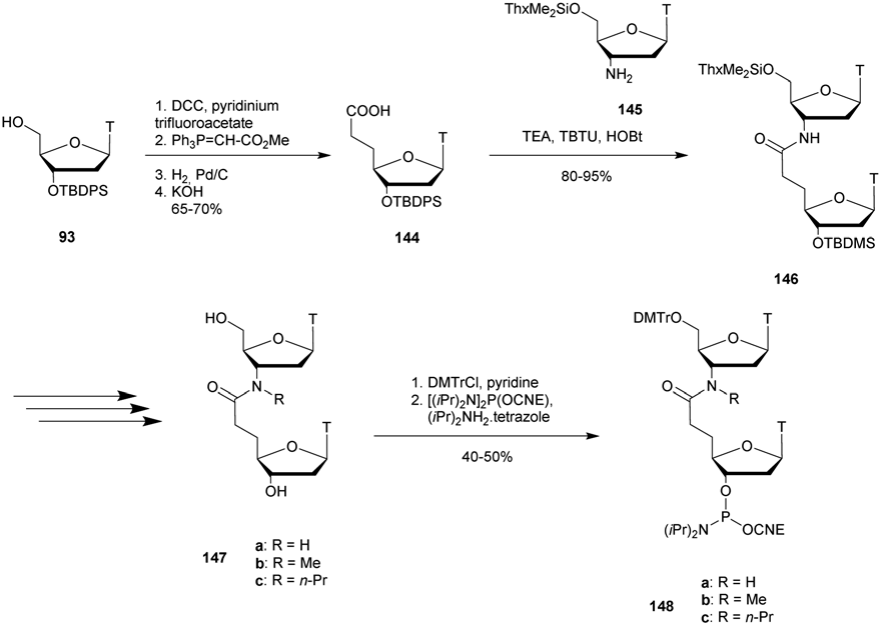
Synthesis of amide-dithymidine phosphoramidite building blocks **148a–c**.

In 1993, Lebreton *et al.* described the synthesis of modified dithymidines bearing an amide linkage (AM).^381^ Their elaboration starts from two synthons previously reported.^382,383^ The 3′-*O*-TBDPS-thymidine **93** was subjected to oxidation with DCC and pyridinium trifluoroacetate and then engaged with a Wittig reagent to give an α,β-unsaturated ester. After reduction of the double bond with H_2_ and Pd/C followed by saponification, the free carboxylic acid **144** was coupled with the 3′-amino thymidine **145** using TBTU as a coupling reagent, leading to compound **146** (Scheme 45). *N*-Alkyl amides were obtained from the protected AM-dithymidine **147**. The bases were then protected with BOM-Cl and then the nitrogen atom of the amide linkage was alkylated using MeI or allyl iodide in the presence of NaH. Full deprotection/reduction followed by 5′ introduction of the DMTr group and phosphitylation led to the desired building blocks **148a–c** (Scheme 45).

**Fig. 20:**
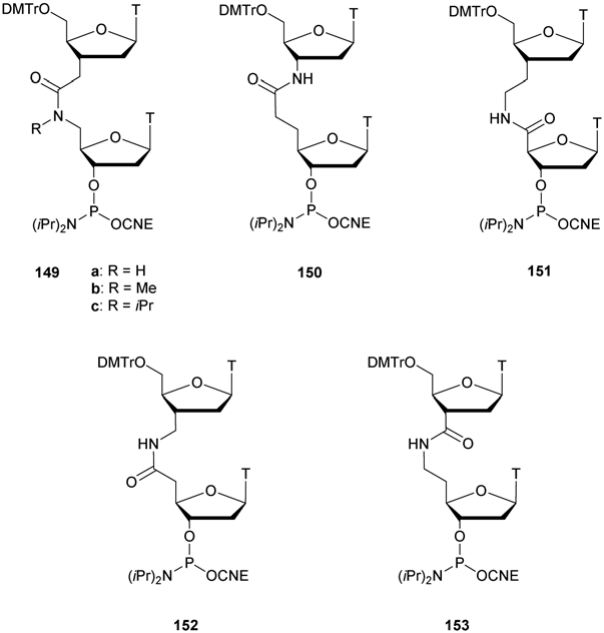
Chemical structures of the AM-dithymidine phosphoramidite building blocks **149–153** synthesised by Just^384^ and De Mesmaeker.^385,386^

Different ODN bearing the three different AM modifications were synthesised. Annealing experiments showed that the AM linkage induces a significant destabilization of the duplexes formed with a complementary RNA strand (Δ*T*_m_ = from −2.2 to −5.4 °C per modification). Thereafter, resistance to nucleases was evaluated in 10% FCS using an ODN bearing a single AM linkage. The authors observed a 2–3 fold resistance increase compared to the unmodified ODN. In parallel, the group of Just published a similar strategy to obtain amide modified dithymidines with an inversion of the position of the amine and the carboxylic acid functions.^384^ They synthesised both amide and *N*-methyl amide modified internucleoside linkages dithymidine phosphoramidites **149a** and **b** (Fig. 20).

After their incorporation within several AM-ODN sequences, annealing experiments showed similar destabilization of the duplexes formed with either DNA or RNA complementary strands. No nuclease resistance study was performed.

Shortly after, another study of De Mesmaeker *et al.* described the synthesis of five AM-dithymidines **149a** and **150–153** (including the one previously described by Just *et al.*) using a similar strategy (Fig. 20).^385^ An annealing experiment with complementary RNA showed a slight destabilization due to the AM-modification (about −1.3 °C per modification) and nuclease resistance was evaluated against 3′-exonucleases in 10% FCS. Five to six times resistance increases were observed for the modification induced by the incorporation of **151**. The nuclease resistance induced by the incorporation of **149a** was reported in another article.^387^ The latter exhibited an enhanced resistance towards hydrolysis by a factor 3 in 10% foetal calf serum.

In 1994, as a continuation of this work, the nitrogen atom of the most interesting AM-modification was alkylated by methyl (**149b**) or isopropyl (**149c**) groups (Fig. 20).^386^ The thermal denaturation studies of the resulting modified ODN were similar to those previously described with only a slight decrease in stability. The nuclease resistance to 3′-exonucleases in 10% FCS with a 3′-end modified AM-ODN was once again studied and the resistance increased by a factor 9 (Me) or 17 (iPr) on the 13-mer d(TCCAGGTGTTT_AM_TC). Clearly this amide modification is more efficient than those previously described probably thanks to the steric hindrance achieved by the isopropyl group that disrupts nuclease activities (Fig. 20).^386^

**Scheme 46:**
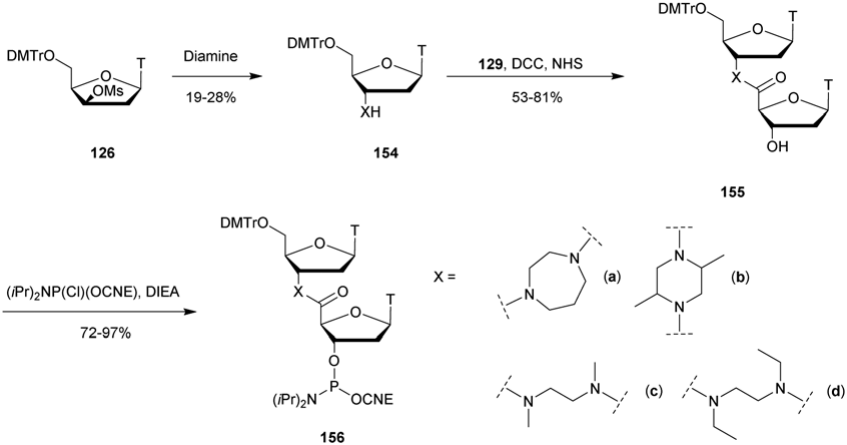
Synthesis of amide-dithymidine phosphoramidite building blocks **156a–d**.

In 1996, Petersen published the synthesis of bulky amide modified internucleoside linkages containing diamines (homopiperazine, 2,5-*trans*-dimethylpiperazine, *N*,*N*-dimethylethylenediamine and *N*,*N*-diethylethylenediamine).^388^ This work was the extension of the piperazine modified linkage described above (see Section 2.2.5).^363^ The synthesis started with the thymidine **126** which reacted with the appropriate diamine in pyridine at 150 °C. Products **154a–d** were then coupled using DCC/NHS with 4′-carboxylic acid-thymidine **131**. The phosphoramidite derivatives **156a–d** were obtained by phosphitylation under classical conditions, allowing the implementation of solid supported ODN synthesis using phosphoramidite chemistry (Scheme 46).

**Scheme 47:**
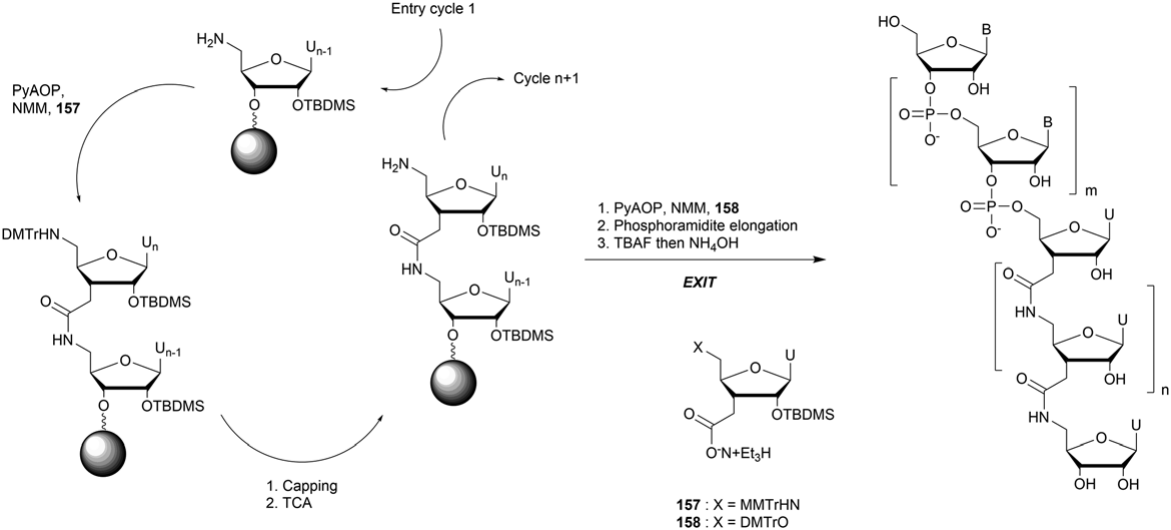
Synthesis cycle of chimeric AM/PO-ODN.

The thermal stabilities of the duplexes formed by various modified ODN with their complementary DNA or RNA strands were studied. The introduction of **156a**, **156c** and **156d** modifications implied a decrease in stability of about −3 °C and −4 °C per modification in the DNA and RNA series respectively. The **156b** modification caused a more pronounced destabilization of −7 and −8 °C respectively. AM-ODN modified at the 3′-end were tested for their resistance to SVPDE. All modified linkages induced a 15 to 20 fold resistance increase of the modified ODN compared to the natural one. In 2007, Iwase *et al.* developed an AM-siRNA.^389^ Modified RNA containing one or two amide linkages at their 3′-end were synthesised *via* peptide coupling on solid supports. The methodology simply consists of using 5′-*N*-MMTr-amino-2′-*O*-TBDMS-3′-carboxymethyl-3′,5′-dideoxyuridine^390^ (**157**) for AM chemistry elongation and 5′-*O*-DMTr-2′-*O*-(TBDMS)-3′-carboxymethyl-3′-deoxyuridine^391^ (**158**) to switch from AM to phosphoramidite chemistry. Coupling reaction with PyAOP reagent in the presence of *N*-methylmorpholine (NMM) has an average yield of 82%. Noteworthily, this strategy allows the elaboration of fully modified ODN. Dimers and trimers of amide-linked oligouridines were prepared and subjected to classical phosphoramidite elongation (Scheme 47). Different sense and antisense 21-mer strands were synthesised and annealed to obtain a modified siRNA targeting firefly luciferase gene sequence. A slight stabilization was observed (about +1 °C) compared to the control siRNA. This result suggests that the presence of the AM-dangling ends (dimers or trimers) at the extremities increases the thermodynamic stability of the duplex. The nuclease resistance was evaluated against nuclease S1. The experiments indicated a significant increase in the half-lives of the modified siRNA (Table 39).

**Table 39:** Thermal denaturation studies (*T*_m_ values) of different AM-siRNA and their half-life evaluations against nuclease S1. Percent inhibition of Luc in A549 cells transfected with pRL luciferase vectors and 20 nM siRNA^389^

siRNA duplex^a^	*T*_m_ (°C)	*t*_1/2_ (min)	Normalized Luc./RL.^b^ (%)
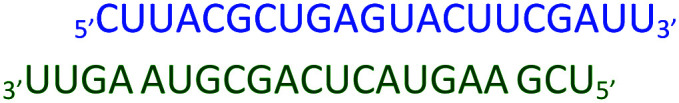	69.5	<10	10.2 ± 1.0
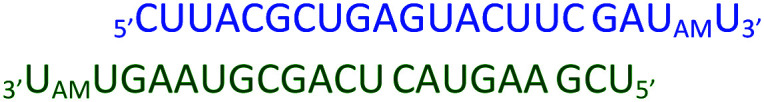	70.3	>120	6.0 ± 1.5
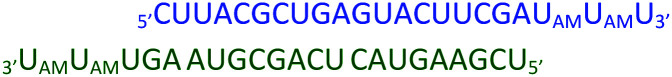	71.5	>120	10.8 ± 0

a AM refers to the amide internucleoside linkage: sense strand (blue) and antisense strand (green).

b Ratios of firefly luciferase activity (Luc.) to *Renilla* luciferase activity (RL.) were normalized to the vector control experiment without siRNA. The half-lives of the siRNA were evaluated against nuclease S1.

Finally, the activity of the modified siRNA was evaluated *in cellulo*. Results demonstrated that the amide-linked RNA segments are tolerated for siRNA gene silencing. In order to go further concerning the study of AM modified siRNA, Iwase *et al.* elaborated longer ODN in order to allow the hybridization of the entire ODN sequence in the absence of dangling ends. The idea was to increase their nuclease resistance with more rigid structures.^392^ Indeed, the authors assumed that the first PO linkage after the AM sequence not located into the double strand structure was the vulnerable point from which endonucleases were recruited. The same chemistry was used^389^ to synthetize second generation siRNA and their melting temperatures were evaluated. Thereafter, they were exposed to SVPDE and FBS digestion (Table 40). A slight increase of the duplex stability and better resistance to nuclease hydrolysis were observed. Whereas the natural siRNA was hydrolysed within a few minutes, the half-lives of the modified siRNA were about 40 min. The remaining siRNA incubated in 10% FBS after 24 h was quantified to be 13% for native siRNA, 47% for the first generation of modified siRNA and 80% for the second generation, demonstrating the potential of the additional duplex structure formed by AM linked RNA and its complementary strand. The ability of this second generation of AM-siRNA to inhibit gene expression was not evaluated.

**Table 40:** Thermal denaturation studies (*T*_m_ values) of AM-siRNA and their half-life evaluations against SVPDE and FBS^392^

siRNA duplex^a^	*T*_m_ (°C)	*t*_1/2_	
		SVPDE (min)	FBS^b^ (%)
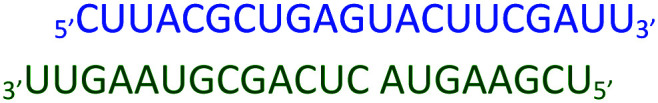	69.5	<5	13
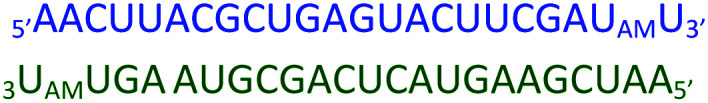	70.5	42	80
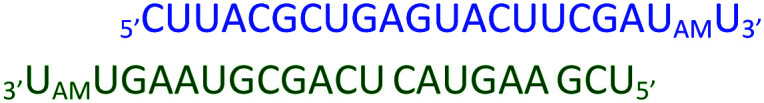	70.3	—	47
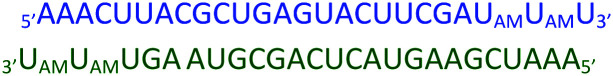	72.5	44	—

a AM refers to the amide internucleoside linkage: sense strand (blue) and antisense strand (green).

b Remaining intact siRNA after 24 h incubation.

At the same time Rozners *et al.* performed X-ray crystallography and siRNA activity assays on modified ODN sequences containing the same amide modified linkages published by Iwase *et al.* The purpose of their study was to determine the structure and the biological activity of modified siRNA.^393,394^ The modifications were incorporated at the 5′-end and at the center of both strands of the siRNA to study their influence. The chemistry used for AM linkage formation was very similar to the one used by Iwase *et al.*,^389,392^ consisting of coupling the primary amine of a 5′-amino nucleoside (A or U) with a 3′-carboxylic acid uridine using HBTU coupling reagent. No further details of this work will be given because no nuclease resistance experiments were performed but it illustrates the potential of AM modifications as mimics of the PO linkage for therapeutic applications due to the preservation of the base-pairing during hybridization. Indeed, although the destabilization was about −3.5 °C per modification, circular dichroism and NMR experiments demonstrated that a typical A-form duplex was formed despite the presence of three consecutive AM linkages.

**Scheme 48:**
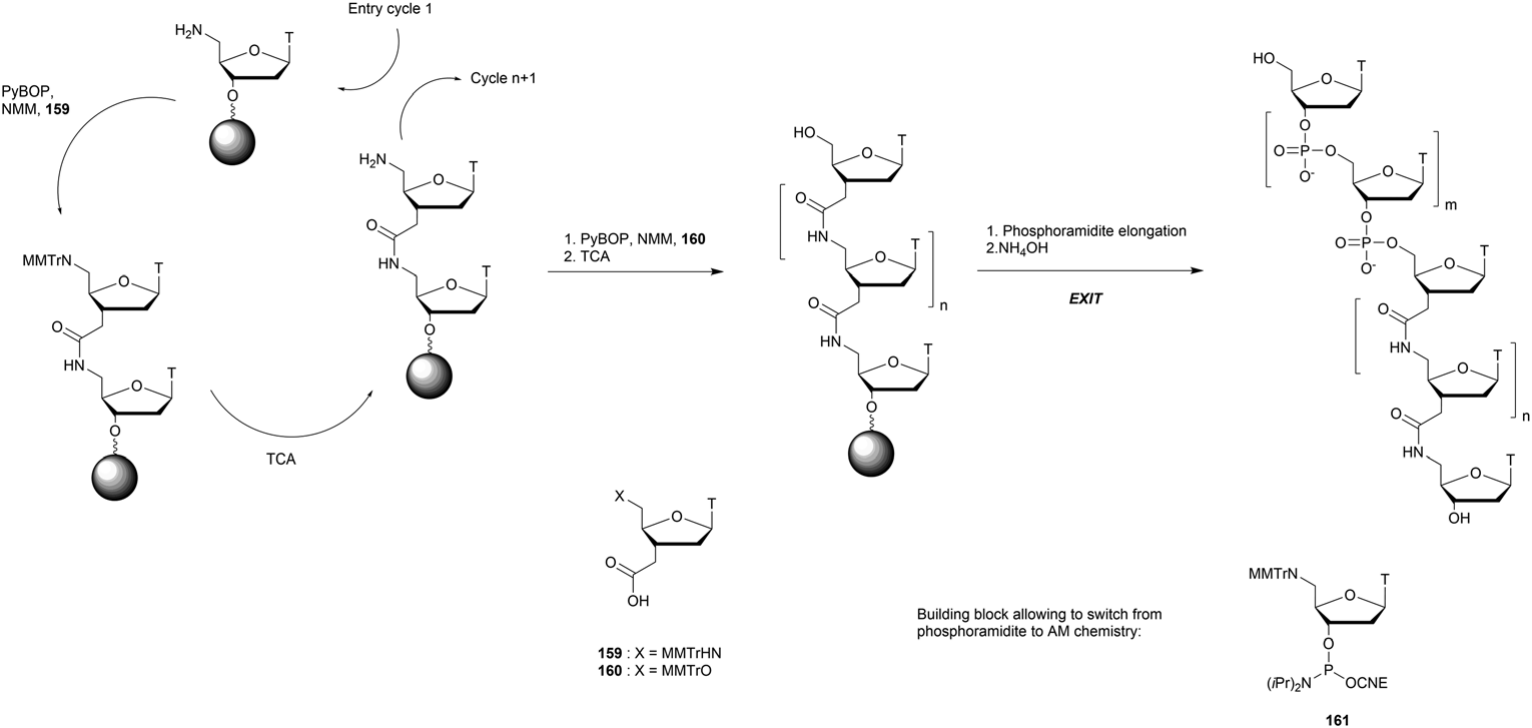
Synthesis cycle of chimeric AM/PO-ODN.

Recently, Brown and co-workers reported the synthesis of chimeric AM/PO-ODN.^395^ The chemistry used is very similar to the one described above for AM-ORN.^389^ The authors exploited the 5′-*N*-(MMTr)amino-3′-carboxymethyl-thymidine (**159)** for coupling reactions on solid supports using PyAOP reagent in the presence of NMM. The transitions between AM- and PO-cycles were achieved using two specific building blocks, **160**, which has previously been described,^396^ and **161**, which is commercially available (Scheme 48). Several chimeric ODN were synthetized. A specific 18-gapmer comprising four AM internucleoside linkages at each extremity was studied and compared to its 2′-OMe analogue and the native PO-ODN. The water solubility of this oligomer was sufficient for biological applications. The thermal stabilities of the duplexes formed with complementary DNA and RNA strands were studied along with resistance to FBS (Table 41). The 2′-OMe modifications induced an increase of the affinity to RNA (+0.25 °C per modification), whereas the AM internucleoside linkage slightly destabilized the duplex (−0.19 °C per modification). Thereafter, *in vitro* experiments were conducted to evaluate the activation of RNase-H using the three ODN. As expected, the native one and the 2′-OMe gapmer were active and induced the total degradation of the targeted RNA strand within 30 min. The AM-gapmer has proven to be as efficient as the positive control tested despite the differences of the measured melting temperatures. Noteworthily, this result is an improvement compared to previous work concerning the use of gapmers with a neutral backbone using PNA that only induces non-catalytic degradation.^397^ The neutral section has to be limited to one wing to trigger catalytic activities.^398^ The nuclease resistance by incubation in FBS resulted in enhanced stability for the AM-gapmer compared to the native one and the 2′-OMe gapmer that were both rapidly degraded even if the 2′-OMe modification slightly improved the nuclease resistance. Finally, cellular uptake experiments within HeLa cell lines were performed using the same sequences with an additional fluorescein tag at their 5′ extremities for confocal microscopy observation. Although all ODN managed to penetrate within the cells, the best results were obtained with the AM-gapmer, demonstrating the potential of the neutral backbone to improve cell penetration. Their uses as gapmers ensure the activation of RNase-H, enabling potential therapeutic application of the AM-linkage within gapmers.

**Table 41:** Thermal denaturation studies (*T*_m_ values) of AM-ODN with complementary DNA or RNA and their half-life evaluations against FBS^395^

ODN (5′ → 3′)^a^	*T*_m_ with DNA (°C)	*T*_m_ with RNA (°C)	*t*_1/2_ b (h)
d(TTTTTCCTGATAGTTTTT)	55.5	56.1	<1
d(T_AM_T_AM_T_AM_T_AM_TCCTGATAGT_AM_T_AM_T_AM_T_AM_T)	52.3	54.6	∼6
d(U̲U̲U̲U̲U̲CCTGATAGU̲U̲U̲U̲U̲)	47.8	58.6	<1

a AM and U̲ refer to the amide internucleoside linkage and 2′-OMe-uridine respectively.

b Approximate values based on raw data.

**Scheme 49:**
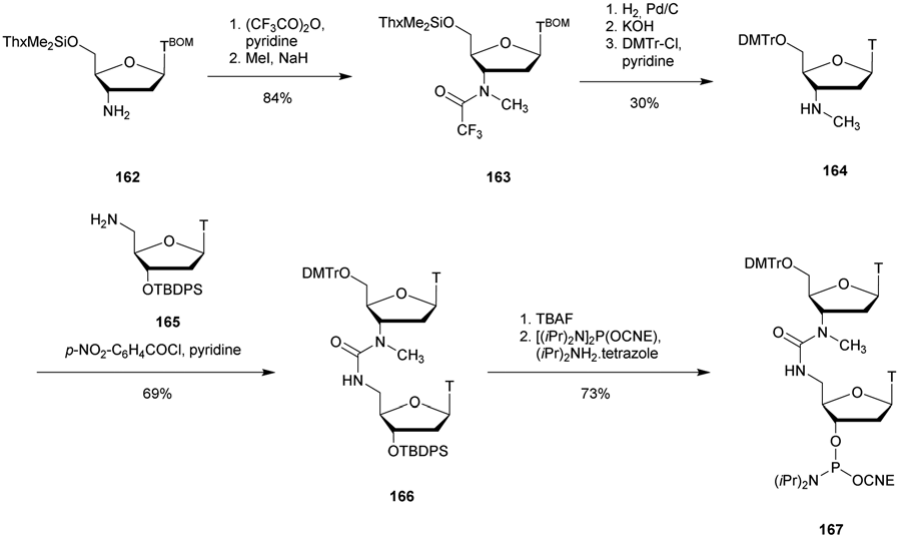
Synthesis of urea-dithymidine phosphoramidite building block **167**.

Structurally very close to an AM linkage, the modification replacing the PO linkage with an urea was developed Waldner and co-workers. Indeed Waldner *et al.* described in 1994 the synthesis of urea (UR) modified dithymidines^399^ and their incorporation within ODN sequences. The synthesis was similar to the work described above concerning the amide internucleoside linkage^381^ with a protected 3′-amino-thymidine, **162**. The latter was subjected to trifluoromethylation followed by methylation of the amide. After full deprotection, the primary 5′ alcohol was protected with a DMTr group. The coupling step was achieved using the *in situ* formation of *p*-nitrophenol to give the urea. The phosphoramidite **167** was obtained after desilylation and phosphitylation of dimer **166** (Scheme 49).

**Scheme 50:**
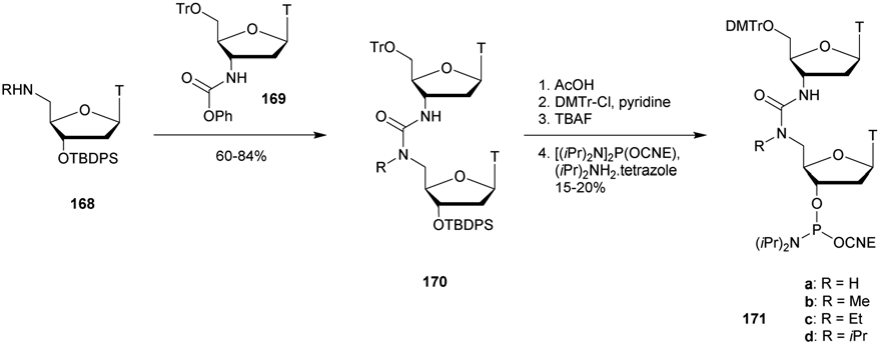
Synthesis of urea-dithymidine phosphoramidite building blocks **171a–d**.

A whole set of alkylated urea derivatives were synthesised from 5′ substituted amines **168a–d** that were coupled with 5′-*O*-Tr-3′-phenylcarbamate-thymidine (**169**), leading to dimers **170a–d**. After several conventional steps the phosphoramidites **171a–d** were obtained (Scheme 50).

Five modified dithymidines were incorporated into ODN sequences and the thermal stabilities of the duplexes formed with complementary DNA and RNA strands were studied. UR modified linkages were introduced at different positions within ODN. Three interesting results have to be pointed out. First, the destabilization was more pronounced when the 3′-nitrogen atom of the linkage was substituted (up to −7.7 °C for one modification), which is a limitation for biological applications that require hybridization of the modified strand. Second, the steric hindrance increases did not correlate with the induced destabilization (the *i*Pr group is more stable than simple H). Finally, there was no significant difference between DNA/DNA and DNA/RNA duplexes. The nuclease resistance was evaluated in 10% FBS using the sequence d(CGACTATGCAATT_UR_TC). The resistance observed was 15 fold higher compared to the natural ODN.

**Scheme 51:**
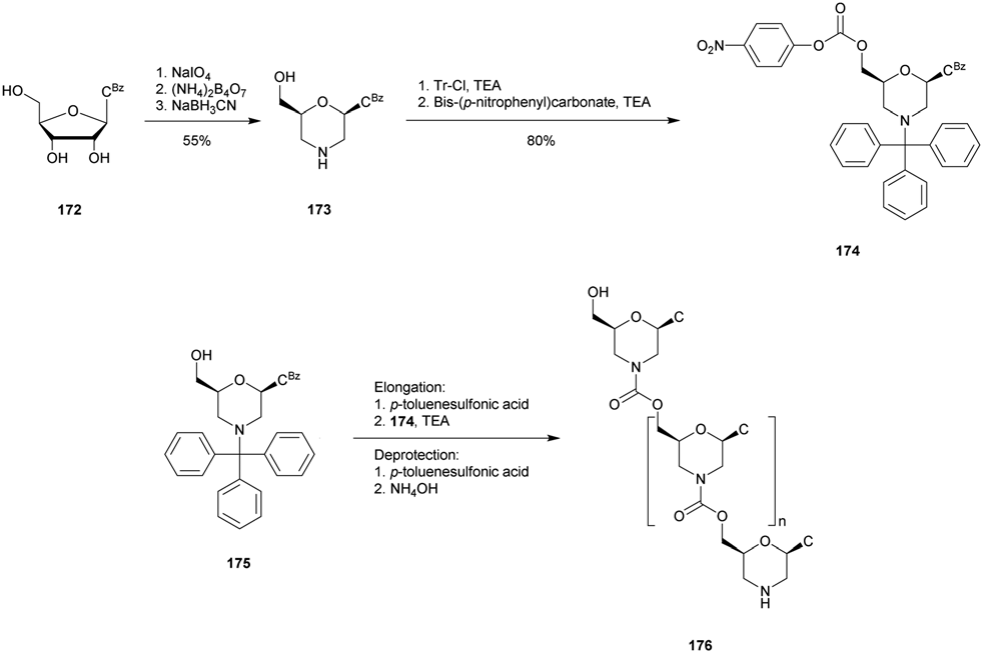
Synthesis of morpholino cytidine oligomers **176** with carbamate internucleoside linkages.

In order to study the possible additional effect of both the amide linkage and ribose modification, Stirchak *et al.* published the synthesis of an original amide modified ODN whose ribonucleic subunit has been modified as a morpholine derivative.^400^ The latter was obtained through an oxidative cleavage of the 2′,3′-dihydroxy ribonucleoside of *N*^4^-benzoyl-cytidine (**172**) followed by reductive amination of the resulting dialdehyde in the presence of ammonia. The amine of the morpholine nucleoside **173** was then protected with a Tr group to give **175**. Activation of the 5′-OH as a *p*-nitrophenylcarbonate, **174**, allowed the synthesis of a dimer. Iteration of this procedure led to the synthesis of various homocytidine oligomers **176** (Scheme 51). Noteworthily, this strategy allows the elaboration of fully modified ODN.

**Fig. 21:**
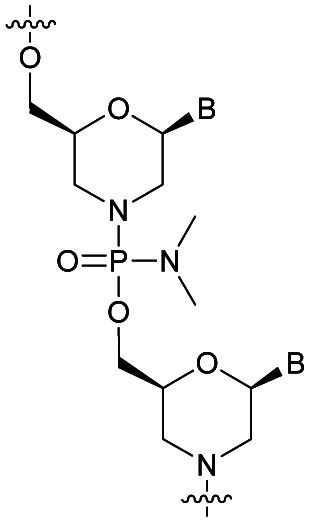
Chemical structure of the phosphorodiamidate morpholino linkage.

A few years later, the synthesis of morpholine nucleosides was improved and extended to all nucleobases.^401^ The modified morpholine nucleosides were used for the synthesis of phosphorodiamidate (PMO) linkages which are derivatives of MO and NP linkages (Fig. 21).^401^

Indeed, this particular class of modified ODN has been extensively studied for their applications in an antisense strategy. Their enzymatic resistance was evaluated by Hudziak *et al.* A fully modified 25-mer (5′-GGUGGUUCCUUCUCAGUCGGACUGG-3′, synthesised by Summerton and Weller procedures^401^) was incubated with various nucleases and esterases and in human serum in order to evaluate their suitability for *in vivo* use.^402^ It was shown that PMO-ODN were totally stable in all the media tested, making them promising candidates for biological applications. A year later, Summerton *et al.* demonstrated their ability to inhibit genetic expression at very low concentrations both *in vitro* (50% inhibition at 10 nM) in the presence of RNase-H and *in cellulo* (41% inhibition at 30 nM).^403^ Since these pioneering studies, many attempts to use the phosphorodiamidate morpholino linkage in antisense therapy have been reported and reviewed recently.^404–407^ As these types of modifications are outside the scope of the present review, these studies will not be detailed further. We have to mention though that in the past few years successful applications of PMO antisense ODN as therapeutic tools against Duchenne muscular dystrophy led to two approved drugs developed by Sarepta Therapeutics: Eteplirsen (Exondys 51®), approved in 2016 by the FDA,^408,409^ and Golodirsen (Vyondys 53®), approved in 2019.^410^

#### 3.2.9 *S*-Methylthiourea (MU) linkage

**Scheme 52:**
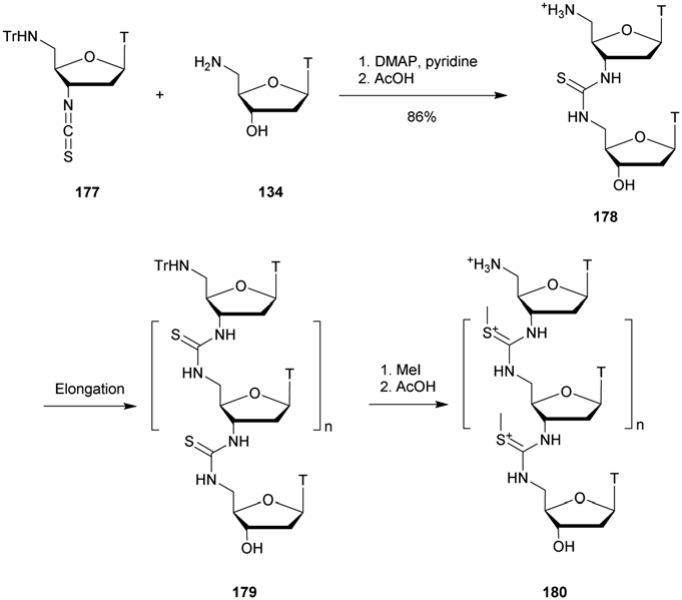
Iterative procedure for MU-homothymidylate **180** synthesis.

In 1998, the group of Bruice described the synthesis of positively charged methylisothiouronium (MU) linkages.^411,412^ The goal was to retain some structural backbone features of the phosphorothioate and methyl phosphonate modifications. The authors first described the synthesis of a 5-mer MU-ODN *via* an iterative procedure using 3′-isothiocyano-5′-*N*-Tr-3′,5′-deoxythymidine (**177**) and 5′-amino-5′-deoxythymidine (**134**) (Scheme 52).^368,369^ The two compounds were condensed in pyridine in the presence of DMAP, affording the 3′ → 5′ thiourea-linked dimer **178** after treatment with AcOH to cleave the Tr protecting group. These two steps were repeated, and once the expected length was obtained, treatment with iodomethane in a mixture of EtOH and DMF followed by final deprotection led to the desired modified ODN **180** (Scheme 52).

The first study published concerned the formation of duplexes with the complementary 5-mer (A_5_) in the DNA or RNA series. Very stable duplexes were formed, especially in the RNA series.^411^*T*_m_ values were more important than those obtained with natural homothymidylates probably due to electrostatic attractions. Moreover, these cationic MU homothymidylates were able to form stable triplexes with a complementary homopolymer of adenosine (molar ratio of 2 : 1).

**Scheme 53:**
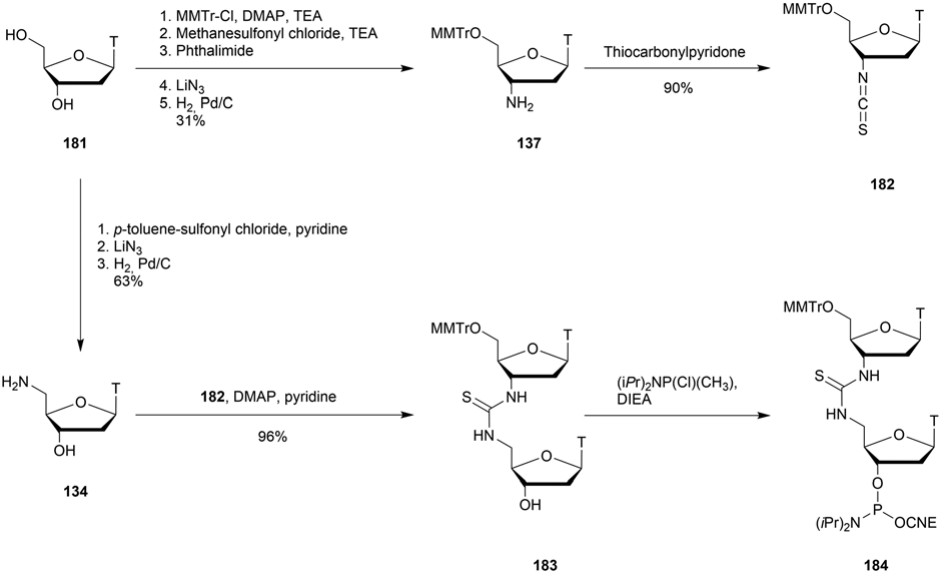
Synthesis of *S*-methylthiourea-dithymidine phosphoramidite building block **184**.

A couple of years later, the same group published a supported synthesis version to obtain polythymidine MU-ODN.^413^ They finally moved to the incorporation of the MU internucleoside linkage into ODN sequences to study both binding characteristics and nuclease resistance.^414^ To achieve this goal, they synthesised a dithymidine building block with a similar optimized chemistry to the one previously described (Scheme 53).

The MU-dithymidine was used as a building block to introduce one or two MU linkages at the desired positions of chimeric 15- or 18-mer ODN. The thermal stabilities of the duplexes formed with a complementary DNA strand were studied. A slight destabilization was observed (about −2 °C with either 1 or 2 modifications). Thereafter, to complete the study, an hybridization experiment was performed by varying the ionic strength of the hybridization buffer. The stability of the duplexes formed between modified strands and their complementary one increased with the ionic strength of the buffer, as for unmodified duplexes. This result indicates that the incorporation of one or two MU linkages into chimeric ODN does not affect the overall electrostatic state of the duplexes formed with a complementary DNA strand. The MU linkage was found to be totally stable against exonuclease I. While the half-life of the unmodified ODN was about 30 min, the 3′ modified ODN was stable to digestion over 12 h. The ODN bearing a MU linkage at the center of the strand was locally hydrolysed until the enzyme reached the modification. The MU modified internucleoside linkage presents the advantage of generating stable duplexes with complementary DNA while providing nuclease resistance. However, hybridization experiments with a complementary RNA strand, digestion experiments with a larger panel of nucleases and RNase-H activation has to be evaluated before considering any uses for biological applications.

#### 3.2.10 Carbamate (CA) linkage

In 1974, Gait *et al.* published the first synthesis of a thymidine dimer analogue bearing a 5′-*N*-carbamate (CA) internucleoside linkage.^415^ They studied its stability in different aqueous buffers and observed good stability against acidic and basic hydrolyses.

**Scheme 54:**
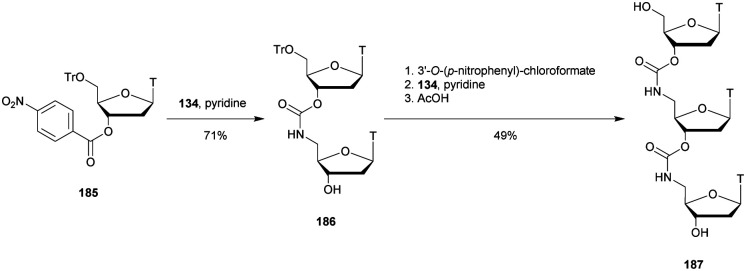
Synthesis of 5′-*N*-CA trinucleoside **187**.

In 1977, Mungall *et al.* published the synthesis of a trinucleotide analogue (**187**) bearing also a 5′-*N*-CA internucleoside linkage by implementing coupling in solution *via* successive protection/coupling/deprotection reactions.^416^ The synthesis was performed by reaction of 5′-amino-5′-deoxythymidine (**134**) with the 3′-*O*-(*p*-nitrophenyl)-carbonate of 5′-*O*-Tr-thymidine, **185**.^417^ The resulting dinucleotide carbamate **186** was again activated at the 3′ position with *p*-nitrophenylchloroformate and condensed as above to give, after detritylation, trimer **187** in 30% overall yield (Scheme 54).

They demonstrated that the linkage was totally resistant to basic (0.1 M NaOH), acidic (0.1 M HCl) and SVPDE hydrolyses. This first test of the resistance of the CA linkage to SVPDE was encouraging to justify further studies.

This was done in 1987 by Couli *et al.* who synthesised a homothymidylate 6-mer bearing five 5′-*N*-CA internucleoside linkages.^418^ The latter was unable to form duplexes with a complementary DNA or RNA strand, demonstrating the strong destabilization implied by the CA linkage.

**Scheme 55:**
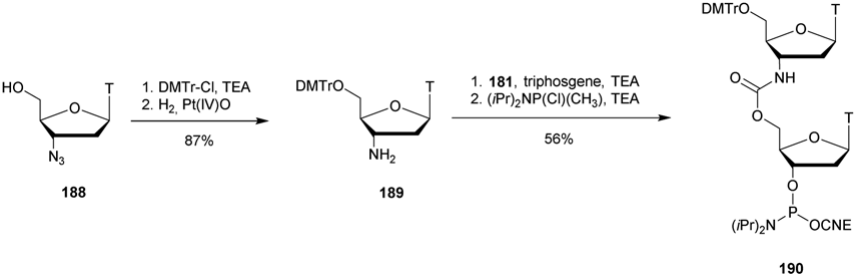
Synthesis of 3′-*N*-CA-dithymidine phosphoramidite building block **190**.

In 1994 the group of Just described the synthesis of 3′-*N*-CA modified dithymidines (along with urea derivatives).^419^ Encouraged by their previous results concerning the amide modified internucleoside linkage,^384^ they decided to study the potential of the carbamate modification. The synthesis was based on the functionalization of 5′-*O*-DMTr-3′-amino-3′-deoxythymidine (**189**) obtained from azidothymidine **188** after 5′-DMTr protection and reduction of the azide group. The carbamate dithymidine was obtained by condensation with thymidine using triphosgene in the presence of TEA. Finally, the phosphoramidite dithymidine **190** was obtained after phosphitylation (Scheme 55).

The carbamate dithymidine was used in automated ODN elongation with an average 95% coupling yield using standard protocols. The authors evaluated the melting temperatures of two ODN bearing one and three modified internucleoside linkages respectively. A strong destabilization of the duplexes was observed (about −8 and −12 °C per modification, respectively, *versus* DNA and RNA strands) just like the inverse carbamate (5′-*N*-CA) described by Couli *et al.*^418^

**Fig. 22:**
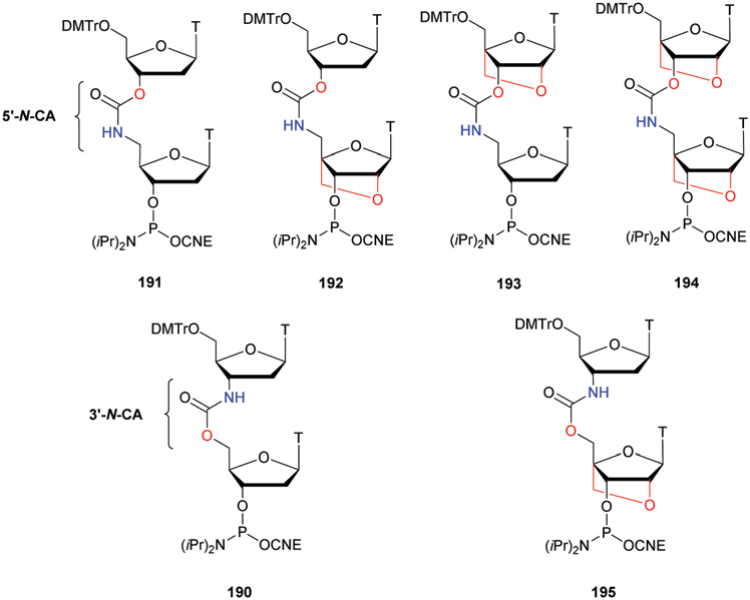
Chemical structures of the 6 CA-dithymidine phosphoramidite building blocks studied by the group of Brown bearing either 5′-*N*-CA or 3′-*N*-CA linkages.

Recently, the group of Brown decided to exploit the strong resistance of the CA linkage to nucleases. In order to counterbalance the low binding affinity of the CA linkage, they chose to use the favourable thermodynamic properties of LNA nucleosides.^420^ Numerous 5′-*N* and 3′-*N*-CA dithymidines combining natural ribose or LNA (CA-LNA) were synthesised using the appropriate amino-thymidine (or LNA thymidine) and activated carbonate intermediates following the strategy previously described.^384^ They were finally converted into phosphoramidite derivatives (Fig. 22), allowing their incorporation within ODN sequences *via* classical phosphoramidite chemistry.

The first studies performed on ODN bearing a unique CA modified linkage without LNA modification confirmed the strong destabilization induced by this linkage. In addition, the authors observed an increased destabilization with 3′-*N*-CA **190** compared to 5′-*N*-CA **191**. Thus, they only studied further the 5′-*N*-CA linkage. A better stability of the duplexes formed with the DNA complementary strand compared to RNA was observed. Thereafter, they studied the expected stabilization induced by the presence of a LNA nucleoside. When introduced on the 5′ side of the CA linkage, an additional destabilization was observed. However, if placed on the 3′ side of the CA linkage a reverse effect was observed (Δ*T*_m_ ∼ +2.6 and +4.1 °C per modification, respectively, with DNA and RNA strands). Finally, addition of LNA nucleosides on both sides of the CA linkage resulted logically in a moderate destabilization with the complementary DNA but surprisingly in the most stable duplex with RNA. This result is probably due to the conformational influence of the LNA carbohydrate. After these preliminary studies, the authors went further working on ODN bearing 3 modified CA linkages. The same deleterious effect on the thermal stability of the duplexes was observed (Table 42). Moreover, the ODN modified with the CA linkage flanked with 2 LNA nucleosides showed also a significant destabilization of the duplexes formed with its complementary DNA strand but good stability with RNA.

**Table 42:** Thermal denaturation studies (*T*_m_ values) of CA-ODN with complementary DNA or RNA and their half-life evaluations against SVPDE and FBS (once the modified nucleoside was reached)^420^

ODN (5′ → 3′)^a^	*T*_m_ (°C) with DNA	*T*_m_ (°C) with RNA	*t*_1/2_	
			SVPDE (min)	FBS (h)
d(GCTTGCTTCGTTCC)	60.2	63.6	<2	<4
d(GCTT^L^GCTT^L^CGTT^L^CC)	—	—	<2	<4
d(GCT^L^T^L^GCT^L^T^L^CGT^L^T^L^CC)	—	—	∼30	>24
d(GCT_CA_TGCT_CA_TCGT_CA_TCC)	52.1	44.8	<2	<8
d(GCT_CA_T^L^GCT_CA_T^L^CGT_CA_T^L^CC)	60.1	59.6	∼15	<8
d(GCT^L^_CA_T^L^GCT^L^_CA_T^L^CGT^L^_CA_T^L^CC)	41.2	61.6	>60	>24

a CA refers to 5′-*N*-CA internucleoside linkages and L to LNA residues.

Finally, enzymatic stability assays were performed to ensure potential biological applications of the CA linkage. First, different ODN were incubated with SVPDE (Table 42). As expected, the natural ODN was fully degraded within a few minutes along with the LNA modified one. The triply modified ODN with LNA dimers was hydrolysed quickly until the enzyme reached the first modification and exhibited a significant increase in resistance contrary to the CA modified ODN that was fully degraded within a few minutes. Finally, the combination of the CA linkage with a single LNA nucleoside increased the resistance of the resulting ODN but the best resistance to nucleases was obtained with the additive effect of the CA modification flanked with two LNA nucleosides. Noteworthily, no water solubility issues were observed. The results presented are in accordance with the work of Mungall *et al.*,^416^ who described the total resistance of the CA containing ODN against SVPDE. Indeed, once again the SVPDE was probably able to “jump” over the modified dithymidine to completely hydrolyse the ODN. The resistance of the ODN was also evaluated against nucleases present in FBS. The hydrolyses were globally slower than that against SVPDE but the order of stability due to the different modifications is consistent. The presence of several consecutive modifications could probably lead to a total stability of the strand. To conclude, the combination of LNA nucleosides along with the 5′-*N*-CA internucleoside linkage is of interest, especially when a CA linkage is flanked with two LNA nucleosides. These modifications provide good stability against nucleases along with a selectivity to form stable duplexes with RNA but not with DNA. Consequentially the CA-LNA modification could be useful for biological applications.

## 4. Conclusions

**Fig. 23:**
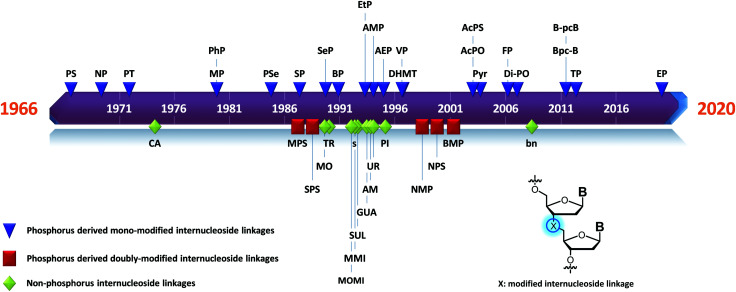
Timeline representing the first publication of the internucleoside linkages described in this review.

Synthetic ODN represent an important class of therapeutic drugs. To fully exploit their potential, it is necessary to prevent their degradation *in vivo* by nucleases while remaining intact in other cellular processes. Chemical modifications are consequentially needed to ensure good stability. Among the different possibilities, the alteration of the internucleoside linkage is particularly efficient as a nuclease recognition site. This review provides an overview of the different modified internucleoside linkages synthesised over the last forty years whose resistances to nucleases have been evaluated. In this context, we report in Fig. 23 the descriptions of all the modified internucleoside linkages described in this review for historical insights. The hybridization properties of the synthesised ODN are provided as well as their ability to allow the activation of RNase-H for possible therapeutic applications *via* the antisense strategy or as duplexes for siRNA gene control. The basic properties of the modified ODN described in this review are summarized in Table 43. Some examples exploit modified internucleoside linkages described for the first time dozens of years ago even if for now the most therapeutically used modification remains the phosphorothioate one. Indeed, new therapeutic PS-ODN were recently approved for commercialization and several recent studies have been devoted to their enantioselective synthesis. This demonstrates that the use of modified ODN in therapy is an established, validated class of drugs that could modulate nearly any genetic target. The six oligonucleotide therapies approved within the last two years elicited unprecedented renewal in the field. Moreover, the first patient-customized ODN-AS therapy (Milasen) recently reported opens up new perspectives for the treatment of genetic diseases without alternative therapies in the future. However, there are challenges remaining to overcome, especially concerning the nuclease resistance, and the potential for future innovation is tremendous. Although PS-ODN are the most widely studied internucleoside modifications, there may be interesting opportunities if researchers are looking more deeply into neglected modified linkages that could lead to major biologically active molecules. Moreover, as shown by the group of Brown with the exploitation of the triazole-modified internucleoside linkage over the past 10 years, some “old” linkages with interesting hybridization and/or nuclease resistance properties can be very useful for various biological applications. Thus, we hope that this review will inspire new research studies to further study underestimated modified linkages but also develop new linkages that have to be discovered. Additionally, the data reported herein can be very useful to researchers willing to develop new internucleoside linkages in the future, and to compare their results with the existing literature before possible exploitation in the fields of therapeutics, diagnostics and molecular biology applications.

**Table 43:** Reported synthetic non-natural internucleotide linkages tested for their resistance to nuclease hydrolysis

Internucleotide linkage	Hybridization efficiency^a^	Nuclease resistance^b^	RNase-H activation^c^	Significant references
Phosphorus derived internucleosidic linkages				
Phosphorothioate (PS)	−	+	✓	88, 96 and 130
Thiophosphate (SP)	−	+	✓	137
Phosphoroselenoate (PSe)	−	+	∅	147
Selenophosphate (SeP)	−	+	∅	151
Phosphoramidate (NP), representative example	−	+	✗	157 and 158
5′-Amino-2′,4′-BNA phosphoramidates (2′,4′-BNA-NP)	++	+	∅	166 and 167
Methyl carbophosphonate (MP)	−	++	✗	172, 175 and 178–180
Phenyl carbophosphonate (PhP)	∅	++	∅	172
Methyl phosphonate LNA (LMP)	++	++	∅	176
Pyridyl carbophosphonate (PyrP)	−	+++	∅	190
Aminomethyl (AMP) carbophosphonate	+ (*R*_p_ isomer); − (*S*p isomer)	∅ (unstable linkage)	∅	191
Aminoethyl carbophosphonate (AEP)	+ (*R*_p_ isomer); − (*S*p isomer)	+++	✗	191
3′-Deoxy-3′-C-(hydroxymethyl)thymidine (3′-DHMT)	−	+++	∅	193
5′-Deoxy-3′-C-(hydroxymethyl)thymidine (5′-DHMT)	−	−	∅	193
3′-Phosphonate (Bpc-B)	—	+ (endonucleases); − (exonucleases)	✗	196
5′-Phosphonate (B-pcB)	−	+ (endonucleases); − (exonucleases)	✗	196
Ethyl (EtP)	−	−	∅	202
Vinyl (VP)	−	+	∅	204
Ethynyl (EP)	—	+	✗	206
Phosphonoacetate carbophosphonate (AcPO)	−	+++	✓	209
Thiophosphonoacetate carbophosphonate (AcPS)	−	+++	✓	209
Phosphonoformate carbophosphonate (PF)	+	+++	✓	212
1,2,3-Triazolylphosphonate carbophosphonate (TP)	−	++	∅	214
Phosphotriester (PT)	−	++	∅	229–233
Diphosphate diester (di-PO)	+	+++	∅	243
Boranophosphate (BP)	−	++	✓ (for chimeric BP/PO-ODN)	273, 277–279, 282 and 284
Methylphosphonothioate (MPS)	−	+	∅	287
Phosphorodithioate (SPS)	−	+++	✓	292 and 293
Thiophosphoramidate (NPS)	+	∅	∅	313–318
Methanephosphonamidate (NMP)	−	+++	∅	319
Boranomethylphosphonate (BMP)	−	++	∅	321 and 322
Nonphosphorus derived internucleosidic linkages				
Triazole	−	+		347 and 348
Dialkyl sulfide (s)	—	+++	∅	353
Sulfamate (SUL)	−	+++	∅	354
Boronate (bn)	−− (*versus* full sequence PO-ODN) ++ (*versus* short PO-ODN)	++	✗	358 and 361
Piperazine (PI)	−−	+	∅	363
Guanidine (GUA)	−−	++	∅	367 and 375
Methylene(methylimino) (MMI)	±	Increased resistance to exonucleases	∅	378
Amide (AM)	−	+	∅	389
Urea (UR)	—	+	∅	399
*S*-Methylthiourea (MU)	−	+++	∅	414
5′-Carbamate (5′-*N*-CA)	−	+	∅	416 and 420
3′-Carbamate (3′-*N*-CA)	—	∅	∅	420

a Hybridization efficiency; − destabilization (∼ from 0 to −3 °C per modification); −− strong destabilization (>−3 °C per modification); + stabilization (∼ from 0 to +3 °C per modification); ++ strong stabilization (>+3 °C per modification); ± no substantial effect; ∅ not tested.

b Nuclease resistance; + moderately increased, ++ highly increased, +++ total resistance; ∅ not tested.

c RNase-H activation; ✓ RNase-H activation; ✗ no RNase-H activation; ∅ not tested.

## Abbreviations

AcPO: Phosphonoacetate
AcPS: Thiophosphonoacetate
AEP: Aminoethylphosphonate
AIDS: Acquired immunodeficiency syndrome
AMD: Age-related macular degeneration
AMP: Aminomethylphosphonate
AMPc: Cyclic adenosine monophosphate
AS: Antisense
9-BBN: 9-Borabicyclo[3.3.1]nonane
BH_3_·Cpy: Borane-2-chloropyridine
BMP: Boranomethylphosphonate
bn: Boronate
BNA: 2′-*O*,4′-*C*-Methylene
BOM: Benzyloxymethyl acetal
BP: Boranophosphonate
Bpc-B: 3′-Phosphonate, base-phosphorus-carbon-base
B-pcB: 5′-Phosphonate, base-phosphorus-carbon-base
BSPDE: Bovine spleen phosphodiesterase II
BSTFA: *N,O*-Bis(trimethylsilyl)trifluoroacetamide
Bzh: 5′-*O*-[Benzhydryloxy-bis(trimethylsilyloxy)-silyl
CA: Carbamate
Cas9: CRISPR associated protein 9
CDN: Cyclic dinucleotide
CIAP: Calf-intestinal alkaline phosphatase
CNE: Cyanoethyl
CMV: Cytomegalovirus
CPG: Controlled pore glass
CRISPR: Clustered regularly interspaced short palindromic repeats
CSPDE: Calf spleen phosphodiesterase
CuAAC: Copper(i)-catalyzed alkyne–azide cycloaddition
DBU: 1,8-Diazabicyclo[5.4.0]undec-7-ene
DCA: Dichloroacetic acid
DCC: Dicyclohexylcarbodiimide
DHMT: Deoxy-3′-*C*-(hydroxymethyl)thymidine
DIAD: Diisopropyl azodicarboxylate
DIC: *N*,*N*′-Diisopropylcarbodiimide
DIEA: *N*,*N*-Diisopropylethylamine
diPO: Diphosphate diester
DMAP: 4-Dimethylaminopyridine
DMSO: Dimethyl sulfoxide
DMTr: 4,4′-Dimethoxytrityl
DNA: Deoxyribonucleic acid
DODSi: Bis-(trimethylsiloxy)cyclododecyloxysilyl ether
ds: Double strand
DTT: dl-Dithiothreitol
EGFP: Enhanced green fluorescent protein
EMA: European Medicines Agency
EtP: Ethylphosphonate
EP: Ethynylphosphonate
ETT: 5-Ethylthio-1*H*-tetrazole
FBS: FBS
FCS: Fetal calf serum
FDA: Food and Drug Administration
FP: Phosphonoformate
GMPc: Cyclic guanosine monophosphate
GUA: Guanidine
HBTU: *N*,*N*,*N*′,*N*′-Tetramethyl-*O*-(1*H*-benzotriazol-1-yl)uronium hexafluorophosphate
HIV: Human immunodeficiency virus
HOBt: Hydroxybenzotriazole
HPLC: High-performance liquid chromatography
hTERT: Human telomerase reverse transcriptase
iBu: Isobutyryl
IBX: *O*-Iodoxybenzoic acid
LMP: Methyl phosphonate locked nucleic acid
LNA: Locked nucleic acid
Luc.: Firefly luciferase
Malat1: Metastasis associated lung adenocarcinoma transcript 1
MCbz: 4-Methoxybenzyloxycarbonyl
MEA: Methoxyethylamine
MMI: Methylene(methylimino)
MNTP: 1,3,2-Diazaphospholidinium hexafluorophosphate
MO: Morpholino phosphoramidate
MoE: 2-Methoxyethyl
MP: Methylphosphonate
MPS: Methylphosphonothioates
Ms: Mesyl
MST: Mesitylenesulfonyl tetrazolide
MU: *S*-Methylthiourea
NEP: 2-Chloro-2-oxo-5,5-dimethyl-1,3,2-dioxaphosphinane
NHS: *N*-Hydroxysuccinimide
NMM: *N*-Methylmorpholine
NMP: Methanephosphonamidates
NP: Phosphoramidate
NPS: Thiophosphoramidate
NTP: Deoxynucleoside triphosphate
OAP: Oxazaphospholidine
ODN: Oligodeoxyribonucleotide
ORN: Oligoribonucleotide
PCR: Polymerase chain reaction
PDE: Phosphodiesterase
PhP: Phenylphosphonate
PI: Piperazine
PMO: Morpholino diphosphorodiamidate
PNA: Peptide nucleic acid
PO: Phosphodiester
PS: Phosphorothioate
PSe: Phorphoroselonate
PSS: Trans-5-benzyl-1,2-dithiane-4-yl
P_S_T: Thiophosphotriester
PT: Phosphotriester
PyAOP: (7-Azabenzotriazol-1-yloxy)tripyrrolidinophosphonium hexafluorophosphate
PyrP: Pyridylphosphonate
RL.: *Renilla* luciferase
RNA: Ribonucleic acid
RNase-H: Ribonuclease-H
s: Dialkyl sulfide
SEM: 2-(Trimethylsilyl)ethoxymethyl
SeP: Selenophosphate
ss: Single strand
siRNA: Small interfering RNA
SP: Thiophosphate
SPAAC: Strain-promoted azide-alkyne cycloaddition
SPS: Phosphorodithioates
SUL: Sulfamate
SVPDE: Snake venom phosphodiesterase
TBAF: Tetra-*n*-butylammonium fluoride
TBAHS: Tetrabutylammonium hydrogen sulfate
TBDMS: *tert*-Butyldimethylsilyl
TBDPS: *tert*-Butyldiphenylsilyl
TBTA: Tris(benzyltriazolylmethyl)amine
TBTU: 2-(1*H*-Benzotriazole-1-yl)-1,1,3,3-tetramethylaminium tetrafluoroborate
TCA: Trichloroacetic acid
TEA: Triethylamine
TEMED: *N*,*N*,*N*′,*N*′-Tetramethylethylenediamine
TFA: Trifluoroacetic acid
THPTA: Tris(3-hydroxypropyltriazolylmethyl)amine
*T*_m_: Melting temperature
TMS: Trimethylsilyl
TMTr: 4,4′,4′′-Trimethoxytrityl
TP: 1,2,3-Triazolylphosphonate
TPS: Triisopropylbenzenesulfonyl
Tr: Trityl
TR: Triazole
Tris: Trishydroxymethylaminomethane
Tse: Trimethylsilylethyl
UR: Urea
VP: Vinylphosphonate

## Conflicts of interest

There are no conflicts to declare.

## Supplementary Material
